# Monodirectional Photocycle Drives Proton Translocation

**DOI:** 10.1021/jacs.3c06587

**Published:** 2023-09-15

**Authors:** Nol Duindam, Michelle van Dongen, Maxime A. Siegler, Sander J. Wezenberg

**Affiliations:** † Leiden Institute of Chemistry, Leiden University, Einsteinweg 55, Leiden 2333 CC, The Netherlands; ‡ Department of Chemistry, Johns Hopkins University, 3400 N. Charles St., Baltimore, Maryland 21218, United States

## Abstract

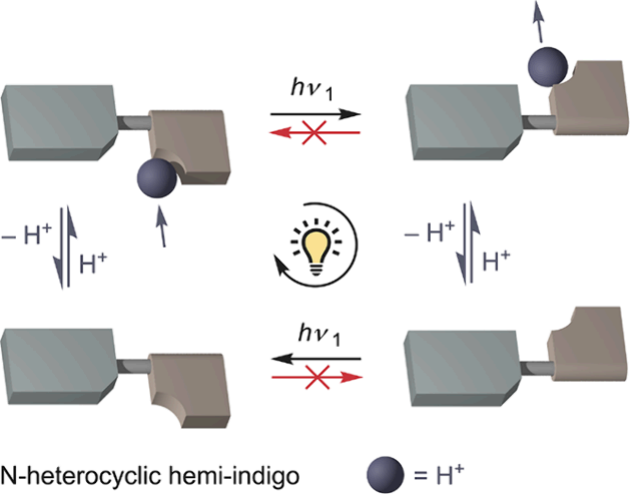

Photoisomerization of retinal is pivotal to ion translocation across the bacterial membrane and has served as an inspiration for the development of artificial molecular switches and machines. Light-driven synthetic systems in which a macrocyclic component transits along a nonsymmetric axle in a specific direction have been reported; however, unidirectional and repetitive translocation of protons has not been achieved. Herein, we describe a unique protonation-controlled isomerization behavior for hemi-indigo dyes bearing N-heterocycles, featuring intramolecular hydrogen bonds. Light-induced isomerization from the *Z* to *E* isomer is unlocked when protonated, while reverse *E* → *Z* photoisomerization occurs in the neutral state. As a consequence, associated protons are displaced in a preferred direction with respect to the photoswitchable scaffold. These results will prove to be critical in developing artificial systems in which concentration gradients can be effectively generated using (solar) light energy.

## Introduction

In bacteriorhodopsin, photochemical all-*trans* to 13-*cis* double-bond isomerization of retinal’s protonated Schiff base is at the basis of a proton translocation event.^1−3^ Release of protons to the extracellular medium and reprotonation from the cytoplasm creates a concentration gradient, which serves as an energy reservoir to produce ATP. Artificial systems that—to some extent—imitate such function^4−6^ may provide a new perspective on solar energy conversion and storage. Hence, synthetic tools to drive substrate translocation are highly sought after, for example, within the field of molecular machines.^7−11^ Indeed, pseudorotaxane structures in which a macrocyclic ring moves repeatedly along a nonsymmetric axle in a specific direction have been reported.^12−18^ Further, controlled displacement of a proton by photoswitchable systems has been shown.^19^ However, repetitive unidirectional translocation of protons has not been achieved to date, while it would be of particular interest and benefit for developing artificial light-driven molecular pumps.^17,18^

Taking inspiration from retinal isomerization, one could think of transporting protons by association with a small-molecule photoswitch. However, once under continuous irradiation a photostationary steady state (PSS) has been established, the rates at which the photoaddressable isomers interconvert into each other are equal. Although forward and backward excited state isomerization paths are different,^20,21^ an associated proton will then be taken in both switching directions, undoing repetitive displacement. We envisioned that if protonation can activate one of the photoswitching directions and suppress the other, repetitive unidirectional translocation becomes viable. In other words, the proton would carry the information that dictates in which direction photoisomerization occurs and, hence, the system operates according to an information ratchet mechanism.^9,22^ As a consequence, machine-like pumping behavior would emerge from a simple molecular photoswitch by harnessing the continuous interconversion between isomers under nonstop irradiation with one and the same wavelength (or a broad spectrum) of light.

We considered hemi-indigo to be suitable for the design of a proton information ratchet for several reasons. While it recently regained attention for its use as molecular photoswitch,^23^ its pyridyl derivative was found to not undergo *Z* → *E* photoisomerization.^24,25^ Conversely, *E → Z* photoisomerization of some structurally related indole-containing hemithioindigo derivatives was found to be suppressed, which was explained by formation of a strong intramolecular hydrogen bond.^26,27^ It has further been shown that incorporation of a pyrrolic ring can energetically favor the otherwise metastable *E* isomer of hemi(thio)indigos owing to this intramolecular hydrogen bond formation.^24−28^ Based on these findings, we hypothesized that protonation of N-heterocyclic hemi-indigos could enable photoisomerization of the *Z* isomer due to disruption of the intramolecular hydrogen bond. On the other hand, in the protonated state, an intramolecular hydrogen bond would be formed in the *E* isomer, which could potentially suppress the photochemical isomerization. In addition, we envisioned that this intramolecular hydrogen bond formation and disruption by (de)protonation could be used to induce reversible thermally activated double bond isomerization, which so far has only been achieved in hydrazones^29,30^ and indigo-derived imines.^31^

**Scheme 1:**
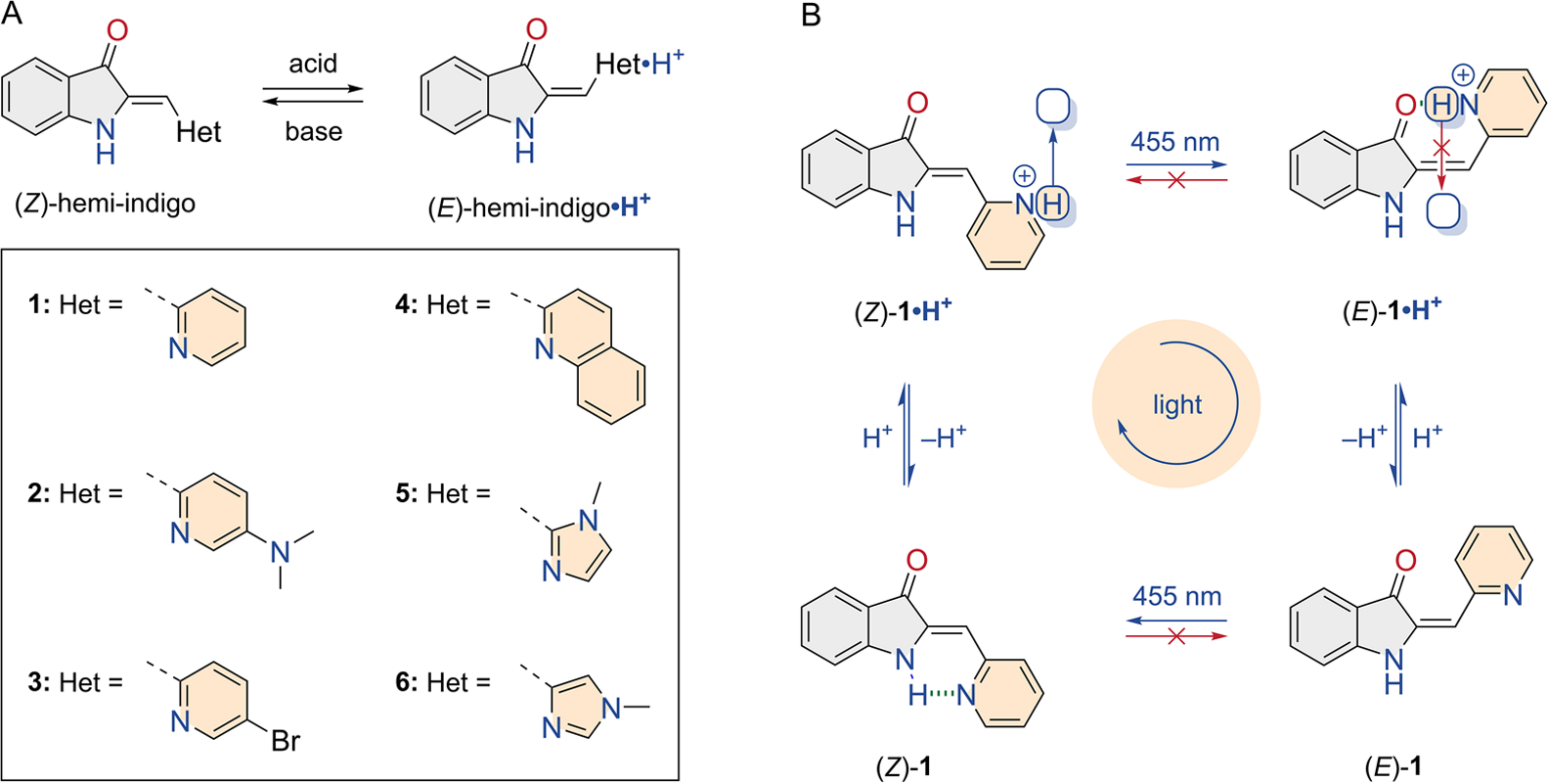
(A) Acid/Base-Controlled Thermally Activated Double Bond Isomerization in Hemi-indigos **1**–**6** and (B) Sequence-Specific Monocyclic Interconversion under Light Irradiation in the Presence of Acid Shown for Pyridyl-Derivative **1** The species are interconverted in the following order: (*Z*)-**1** → (*Z*)-**1·H**^**+**^ → (*E*)-**1·H**^**+**^ → (*E*)-**1** → (*Z*)-**1**. As the forward isomerization takes place in the protonated state and the backward isomerization occurs after deprotonation, protons are displaced effectively (from bottom to top with respect to hemi-indigo as indicated by the blue arrow) under continuous 455 nm irradiation.

Herein, it is demonstrated for six different N-heterocyclic hemi-indigos (Scheme 1A) that the forward *Z* → *E* photoisomerization takes place only when protonated, while the reverse *E* → *Z* photoisomerization occurs in the neutral state. One-way and virtually quantitative conversion is therefore attained in single and opposite directions for the neutral and protonated species. Since in the presence of acid, the neutral and protonated species are in equilibrium, they are interchanged photochemically in a specific order under irradiation with one and the same wavelength of light as is illustrated in Scheme 1B. We envision that associated protons are thereby displaced in one direction with respect to the photoswitchable hemi-indigo scaffold. In addition, under conditions different than those in the irradiation experiments, nearly quantitative and reversible conversion from the *Z* to *E* isomer and *vice versa* is induced by acid and base, respectively, and these hemi-indigos thus constitute a new class of pH-responsive switches.

## Results and Discussion

### Energy Minimization by DFT

**Figure 1:**
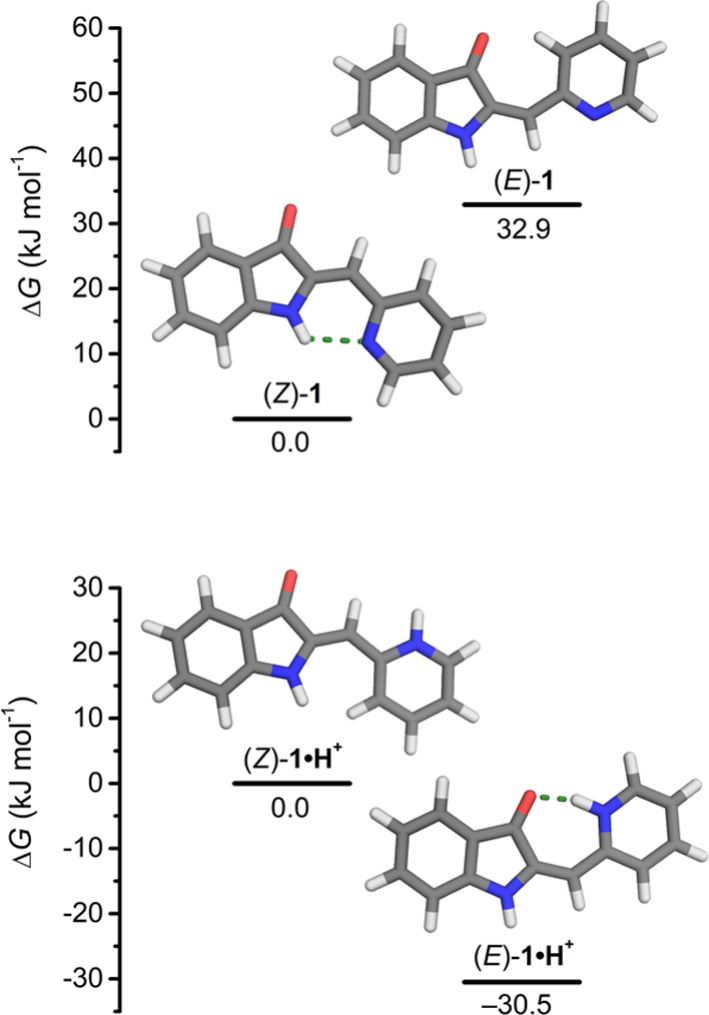
DFT-minimized geometries and plots of the relative Gibbs free energies; color codes: hydrogen bond = green; O = red; *N* = blue.

To gain insights into the stabilizing effect of intramolecular hydrogen bonding in the ground state, the geometries of possible isomers of hemi-indigo pyridyl derivative **1** were minimized by density functional theory (DFT, B3LYP/6-311++G(d,p) level of theory and an IEF-PCM CHCl_3_ solvent model, see the SI for details). For the *E* and *Z* isomers, in both their neutral and protonated states, two possible rotational isomers were considered (Scheme S2). The energetically most favored conformations are depicted in Figure 1 alongside the relative energies. As anticipated, in (*Z*)-**1**, a hydrogen bond is formed between the pyridyl nitrogen and the N–H atom of the 3-oxindole fragment [N(H)···N distance = 2.81 Å), which is not possible in (*E*)-**1**. Conversely, in the protonated state of the former isomer, the pyridinium nitrogen is rotated away from the N–H hydrogen bond donor and not involved in hydrogen bonding. Now instead, a stabilizing hydrogen bond can be formed in the *E* isomer, i.e., between the pyridinium proton and the carbonyl oxygen atom [N(H)···O distance = 2.64 Å]. Owing mainly to this intramolecular hydrogen bond formation, there is a substantial thermodynamic preference for the *Z* over the *E* isomer in the neutral state (Δ*G* = 32.9 kJ mol^–1^), whereas in the protonated state, the *E* isomer is considerably lower in energy (Δ*G* = −30.5 kJ mol^–1^).

### Synthesis and Crystallographic Analysis

**Scheme 2:**
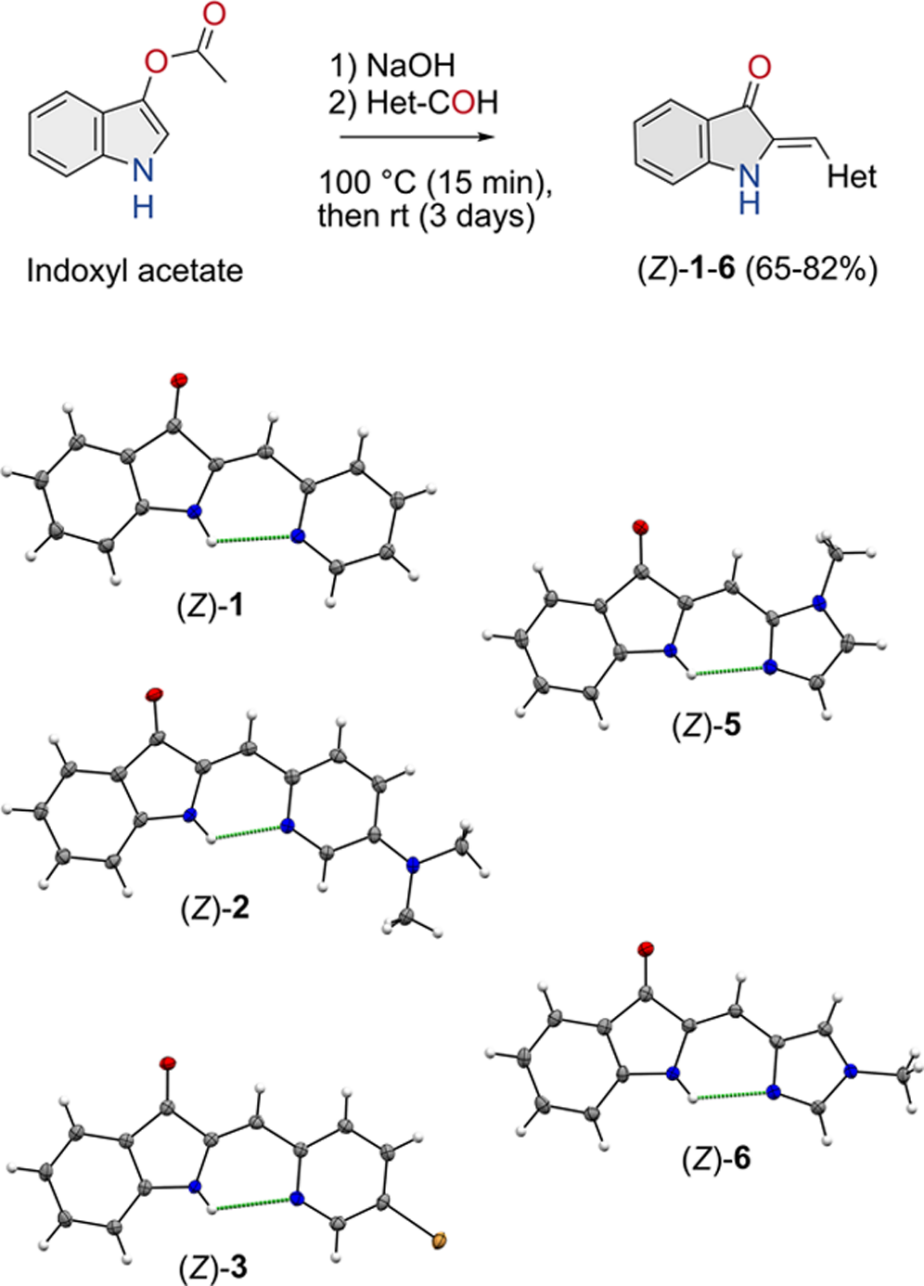
Synthetic Step toward Hemi-indigos **1**–**6** and below the Displacement Ellipsoid Plots (50% Probability Level, 110(2) K) of the Products for which Crystal Structures Were Obtained

We then synthesized hemi-indigo **1** as well as derivatives **2**–**6**, which contain different N-heterocycles, through a condensation reaction of commercially available indoxyl acetate with the respective aldehydes (Scheme 2).^23,32^ The imidazole rings in compounds **5** and **6** were methylated to avoid tautomerization. The desired products were obtained in good yields (65–82%) as the thermodynamically most stable *Z* isomers (*Z*/*E* ratio > 99%). Importantly, the ^1^H NMR spectra recorded in CDCl_3_ revealed downfield-shifted NH signals for all compounds (δ = 10.47–9.14 ppm) with respect to parent hemi-indigo containing a phenyl instead of the N-heterocyclic ring (**7**, δ = 6.82 ppm, Figure S13), which indicates involvement of the NH proton in hydrogen bonding with the nearest nitrogen atom of the N-heterocycles.

All products, except quinolyl derivative **4**, were additionally characterized by single crystal X-ray crystallography (see Scheme 2 and the SI for details). The solid-state structures are (as expected) of the energetically most stable *Z* isomers. The structural similarity between these *Z* isomers is high, i.e., the torsional angles between 3-oxindole and N-heterocycle moieties are all below 3**°** and the N(H)···N bond distances are found between 2.72 and 2.86 Å, which is within the hydrogen bond range (Table S6). The presence of a stabilizing intramolecular hydrogen bond is thus supported in all cases. Important to note is that for (*Z*)-**1**, this hydrogen bond distance (2.82 Å) is similar to that in the DFT-calculated structure (2.81 Å).

### Acid-Induced Thermal Isomerization

**Figure 2:**
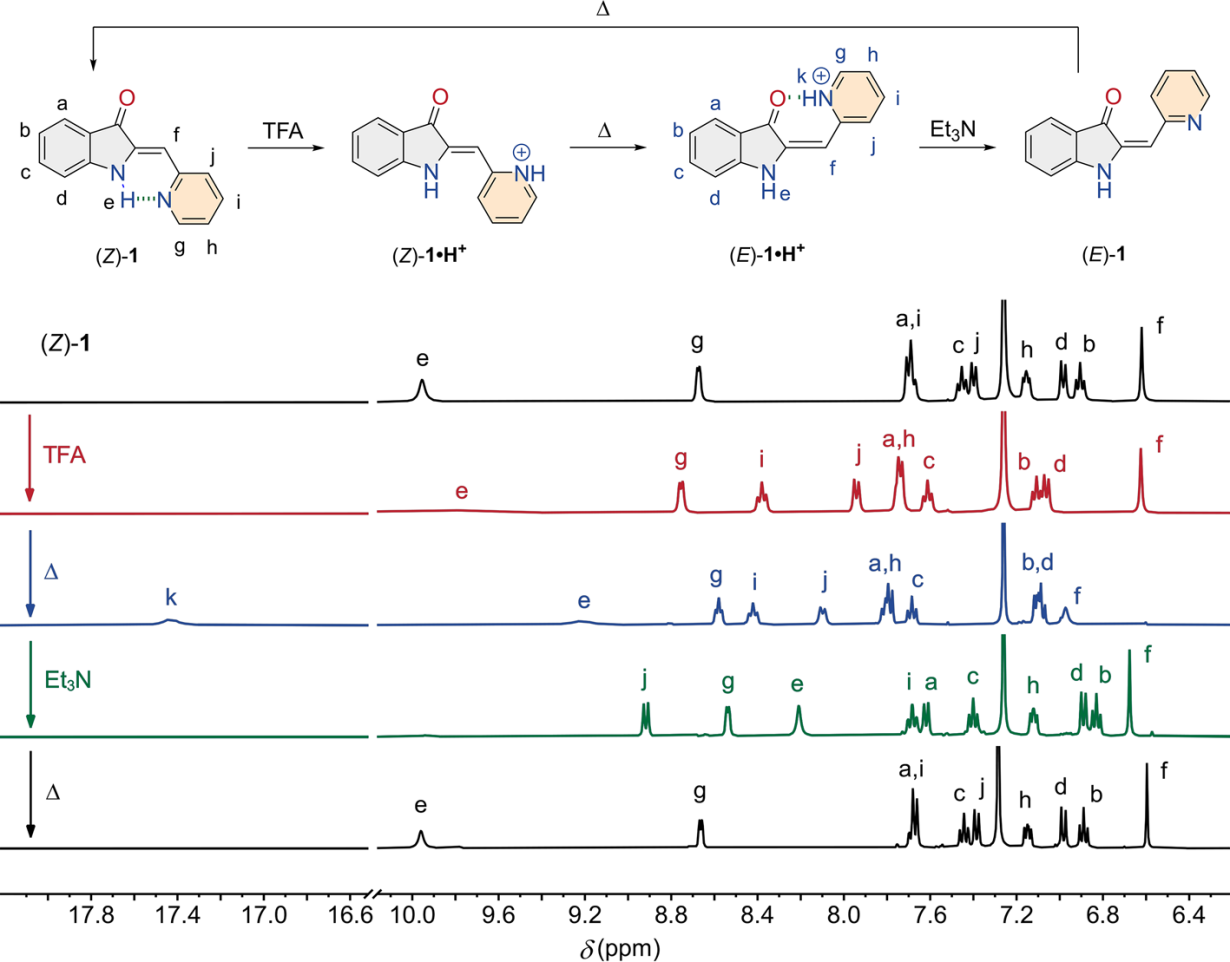
Selected regions in the ^1^H NMR spectrum (400 MHz) of (*Z*)-**1** (5.7 mM in CDCl_3_) before and after addition of TFA (32 equiv) to give (*Z*)-**1·H**^**+**^ followed by flame-sealing of the NMR tube and thermal equilibration at rt for 3 days to afford (*E*)-**1·H**^**+**^, opening of the NMR tube with a diamond glass file and addition of Et_3_N (48 equiv) to give (*E*)-**1**, and flame-sealing of the NMR tube and thermal equilibration at rt for 9 days to reobtain (*Z*)-**1**.

Initially, we studied whether thermal isomerization to the *E* isomer could occur upon protonation using ^1^H NMR and UV–vis spectroscopy. Incremental addition of trifluoroacetic acid (TFA, from 0 to 4 equiv) to (*Z*)-**1** in CDCl_3_ resulted in clear downfield shifting of all pyridyl ^1^H NMR signals (H_j_, H_i_, H_h_, and H_g_), indicative of protonation (Figure 2 and the SI for details). Concomitantly, the olefinic proton signal (H_f_) shifted upfield, whereas for the aromatic 3-oxindole protons (H_a-d_), only minor chemical shift changes were observed, and the signal belonging to the NH-proton (H_e_) shifted and broadened. At first, saturation seemed to be reached at around 4 equiv, but when adding more TFA (from 4 to 40 equiv), some of the chemical shift changes reversed, most notably for the pyridyl *ortho*-proton (H_g_) and the olefinic proton (H_f_), pointing at a secondary process. We hypothesized that it could be related to the solvation of an initially formed pyridinium–trifluoroacetate ion pair.^33^ Indeed, when a ^1^H NMR titration experiment was carried out with pyridine instead of (*Z*)-**1**, a similar trend was observed (Figure S22). We therefore presume that the extent of ion-pairing between (*Z*)-**1·H**^**+**^ and the trifluoroacetate counteranion is reduced when more TFA is present, accounting for the reverse in chemical shift changes beyond the addition of 4 equiv in the titration.

When a solution containing excess TFA (32 equiv) was then left to equilibrate, the ^1^H NMR signals for the protonated species (*Z*)-**1·H**^**+**^ disappeared and a new set of signals emerged (Figure 2 and Figure S25). A NOE-diff. experiment revealed a through-space interaction between the olefinic proton (H_f_) and the 3-oxindole NH-proton (H_e_) (Figure S37), supporting formation of the *E* isomer. Furthermore, the NOESY spectrum of (*E*)-**1·H**^**+**^ indicated that the olefinic proton (H_f_) is in close proximity to an N-heterocyclic proton (H_j_), while no coupling was observed with the pyridinium proton (H_k_) (Figure S38). Very likely, hydrogen bonding between the pyridinium and carbonyl group leads to a fixed orientation of the N-heterocycle, as was predicted by DFT calculations. Remarkably, within a day, nearly quantitative isomerization to the *E* isomer was observed, i.e., a mixture enriched to 95% in (*E*)-**1·H**^**+**^ was obtained at thermal equilibrium. When the amount of TFA was decreased, however, the *E*/*Z* ratio decreased (Figures S23 and S24), even while protonation of **1** was established. A possible explanation was sought in pyridinium–trifluoroacetate ion-pair formation, which can compete with the stabilizing intramolecular hydrogen bond formation. This ion-pair is proposed to be solvated in the presence of larger amounts of TFA,^33,34^ explaining the difference in conversion to the *E* isomer. Further, increasing the amount of TFA accelerated isomerization (Figures S39–S41), hinting at an acid-catalyzed process. A similar observation was made previously for other conjugated olefins, including stilbene derivatives^35−37^ and diaryl-hemiindigos.^38^

Interestingly, the *Z* isomer could be regained by the basification of the solution. When Et_3_N was added (48 equiv) to an equilibrated sample of **1** in the presence of TFA (32 equiv), most pyridyl proton signals now shifted back upfield (H_g_, H_h_, H_i_, Figure 2 and Figure S26), indicative of deprotonation. Over the course of several days, (*E*)-**1** fully isomerized back to (*Z*)-**1**, hence completing the thermal acid/base-controlled switching as outlined in Scheme 1A.

For hemi-indigos **2**–**6**, the thermal isomerization behavior was comparable (Table S7). The dimethylamino- and bromo-substituted pyridyl derivatives **2** and **3**, as well as the quinolyl derivative **4**, showed identically high conversion to the *E* isomer when protonated (>95%), while differences in isomerization rates among the different compounds were small. For imidazole-based derivatives **5** and **6**, conversion to the *E* isomer was lower (85 and 88%, respectively), and while the former compound exhibited the slowest isomerization in the series, this process was the fastest for the latter. After neutralization of the solution by Et_3_N addition, all compounds displayed quantitative isomerization back to their *Z* isomer, and in this case, large variations were observed in the *E* → *Z* isomerization rate (Table S7). It is worth noting that for structurally related hemithioindigo, Hammett analysis indicated that electron-donating *para*-substituents can lower the thermal isomerization barrier, most likely because of the increase in donor–acceptor character.^39^ To summarize, by varying the N-heterocyclic ring the thermal isomerization rates can be tuned.

**Figure 3:**
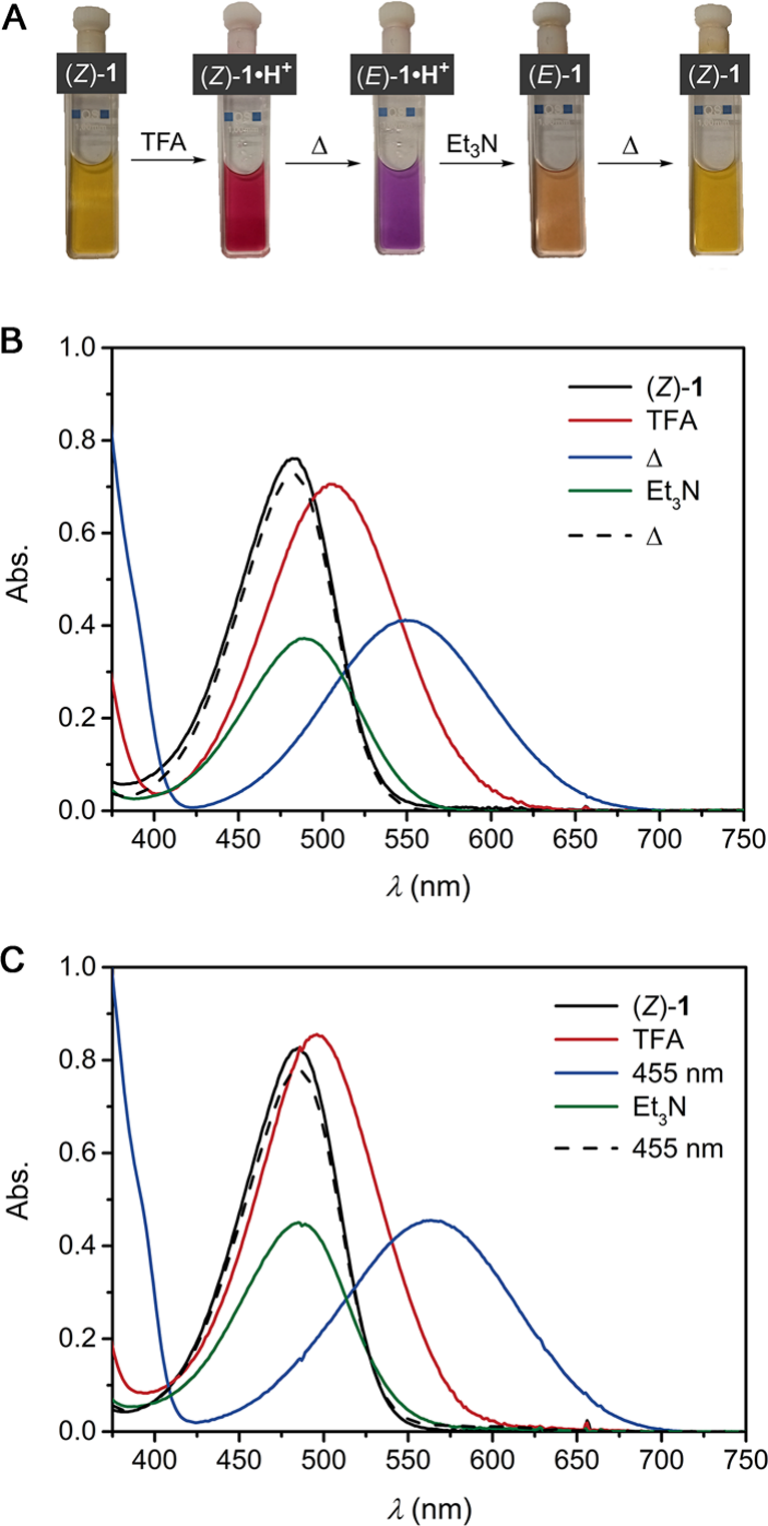
(A) Naked-eye color changes corresponding to the UV–vis thermal equilibration experiments. (B) UV–vis spectral changes starting with a solution of (*Z*)-**1** (0.76 mM in degassed CHCl_3_, 1 mm quartz cuvette) before and after addition of excess TFA (4.3 × 10^2^ equiv) followed by equilibration at rt for 15 h, treatment with Et_3_N (1.6 equiv with respect to TFA), and equilibration at rt for 24 h. Please note that direct addition of TFA and Et_3_N to the same solution of (*Z*)-**1** also results in a slight decrease in molar absorptivity, as is observed here after the full switching cycle has been completed (Figure S53). (C) UV–vis spectra starting with (*Z*)-**1** (0.076 mM in degassed CHCl_3_, 1 cm quartz cuvette) where irradiation with 365, 385, 455, 465, and 525 nm for 2–10 min did not cause any spectral changes, yet after addition of TFA (5.5 μL, 4.7 × 10^2^ equiv) and irradiation with 455 nm, the absorption shifted bathochromically. Subsequent irradiation with 365, 385, 455, 465, 525, 591, 630, and 660 nm for 2–10 min did not cause any spectral changes, yet after addition of Et_3_N (15 μL, 7.0 × 10^2^ equiv) and irradiation with 455 nm, the original UV–vis absorption spectrum of (*Z*)-**1** was regained.

Protonation and thermal *E*/*Z* isomerization were accompanied by distinct color changes and were additionally monitored by UV–vis spectroscopy (Figure 3A,B and Figures S53–S64). All compounds absorbed in the visible region having absorption maxima between 478 and 508 nm. In the case of pyridyl derivative (*Z*)-**1**, addition of TFA was accompanied by a large bathochromic shift (λ_max_ = 482 to 504 nm). When the solution was allowed to equilibrate, the absorption shifted to a longer wavelength (λ_max_ = 549 nm) and decreased in intensity. This red-shifted absorption is in agreement with time-dependent DFT calculations for (*Z*)-**1·H**^**+**^ (λ_max_ = 505 nm) and (*E*)-**1·H**^**+**^ (λ_max_ = 554 nm) and, thus, supportive of *E* → *Z* isomerization (Figure S115). Subsequent addition of excess Et_3_N to basify the solution led to a hypsochromic shift (λ_max_ = 489 nm), and over time, the original UV–vis spectrum was recovered, demonstrating reversibility of the protonation-induced isomerization.

For hemi-indigos **2**–**4**, similar shifts were observed upon TFA addition (Figures S53–S60), whereas the imidazole-based derivatives **5** and **6** mainly showed a decrease in absorptivity with only a minimal change in absorption maximum (Figures S61–S64). Nevertheless, in all cases, *Z* → *E* isomerization was accompanied by a large bathochromic shift, and clear isosbestic points were observed.

### Monodirectional Photoisomerization Cycle

After having identified the UV–vis absorbance and ^1^H NMR signatures of all of the neutral and protonated isomers, we set out to investigate the photoisomerization behavior. It is important to note that UV–vis irradiation studies were performed at lower temperatures (≤0 °C) and higher dilution than the isomerization experiments described above to suppress the thermally activated process. In line with reported data,^24,25^ exposure of a solution of (*Z*)-**1** to various wavelengths of light did not cause any UV–vis spectral changes (Figure S65), which confirms that photochemical isomerization is inhibited. To our delight, after the addition of TFA, irradiation at 455 nm afforded a bathochromically shifted absorbance profile similar to the one observed for the thermally generated (*E*)-**1·H**^**+**^ species (Figure 3C), thus revealing that photoisomerization can occur in the protonated state. Likewise, in the ^1^H NMR spectrum, the same set of signals that was observed upon acid-induced thermal isomerization appeared after irradiation of a solution of the *Z* isomer in the presence of TFA, further supporting that the *E* isomer can be generated photochemically upon protonation (Figure S84).

Strikingly, when a sample of (*E*)-**1·H**^**+**^ (generated using 455 nm light) was subsequently exposed to other wavelengths, including those at which only this protonated isomer and not the (*Z*)-**1·H**^**+**^ species absorbed (>625 nm), no spectral changes were noted. This result suggests that backward photoisomerization is inhibited in the protonated state, potentially due to excited state intramolecular proton transfer (ESIPT), as has been demonstrated for indigo,^40^ and was similarly observed for (*Z*)-**1**^24,25^ and the *E* isomer of structurally related indole-derived hemithioindigo.^27^ Yet, addition of excess Et_3_N, which afforded the absorption spectrum characteristic for (*E*)-**1** (Figure 3C), allowed full isomerization back to the original (*Z*)-**1** upon irradiation at 455 nm. Forward and backward isomerization reactions were thus achieved in respective protonated and neutral forms by using light of the same wavelength, where thermal isomerization was negligible under the experimental conditions used.

The same acid/base-controlled photoresponsivity was observed for hemi-indigos **2**–**6** (Figures S68–S83). Interestingly, for dimethylamino-substituted derivative **2**, the entire cycle could be performed using 525 nm instead of 455 nm light owing to its red-shifted absorption (λ_max_ = 511 nm, Table S9 and Figures S68–S70). For (*Z*)-**3**, a larger amount of TFA was required for successful photoconversion (Figures S71–S73) and, besides, generation of (*E*)-**6**·**H**^+^ was limited by poor photostability of (*Z*)-**6·H**^**+**^ (Figures S80–S83). Nevertheless, when (*E*)-**6·H**^+^ was first accessed *via* thermal isomerization, subsequent photoisomerization from (*E*)-**6** to (*Z*)-**6** proceeded smoothly under 455 nm irradiation after Et_3_N addition.

The quantum yields for 455 nm-induced *Z* → *E* isomerization in the protonated state and *E* → *Z* isomerization in the neutral state were determined using potassium ferrioxalate as an actinometer. By monitoring the UV–vis absorbance changes upon irradiation of concentrated solutions of the *Z* isomer in the presence of excess TFA, the rates of formation of the protonated *E* isomers were determined. Using the photon flux, the quantum yields were then calculated, which ranged around 1% for compounds **1**–**4** and was significantly lower for **5** (see Table 1 and the SI for details). The rates of formation of the neutral *Z* isomers were determined by first generating the *E* isomers (by thermal equilibration) in the presence of excess TFA, which was followed by neutralization with Et_3_N and 455 nm irradiation. Here, the variation in quantum yield between the different N-heterocyclic hemi-indigos was larger, with the highest values (≥3.5%) found for **2** and **5** and that of **1** and **3** being about 10 times lower.

**Table 1:** Photoisomerization Quantum Yields of Hemi-indigos **1**–**5**^a^^b^

compound^c^	Φ_*Z*·H^+^→*E*·H^+^_ (%)	Φ_*E*→Z_ (%)
**1**	0.76 ± 0.011	0.34 ± 0.003
**2**	1.23 ± 0.014	3.9 ± 0.035^d^
**3**	0.85 ± 0.003	0.24 ± 0.010
**4**	0.88 ± 0.003	n.d.^e^
**5**	0.20 ± 0.001	3.5 ± 0.099^d^

a At 455 nm irradiation in CHCl_3_ at 273 K, unless otherwise noted.

b Standard deviations are from duplicate measurements.

c Not determined for compound **6** because of poor photostability.

d Determined at 263 K.

e Not determined because of rapid thermal *E* → *Z* isomerization of compound **4**.

These results confirm that *Z* → *E* isomerization is enabled in protonated form, while the reverse isomerization process takes place in neutral form. To the best of our knowledge, such a feature has not been observed in photoswitchable systems before. It is also fundamentally different from earlier reported examples of acid-gated photoresponsivity,^37,41−43^ which allowed both switching directions in at least one of the states. In our case, only a single (and opposite for neutral and protonated forms) direction of photoswitching is suppressed, affording quantitative conversion by default. It leads to a unique monodirectional cycle of interconversion between species under continuous illumination once a protonation equilibrium is established, thereby displacing associated protons in a specific direction.

## Conclusions

In summary, unprecedented photo- and thermal isomerization behavior was discovered for N-heterocyclic hemi-indigos. Disruption and (re)formation of intramolecular hydrogen bonding interactions were found to be of major importance. It must be recognized that beside hydrazone,^30^ these compounds represent the second family of switches that undergo reversible pH-activated double bond isomerization. More striking though is the observed protonation-controlled one-way photoisomerization, giving rise to an exceptional monodirectional interconversion cycle. Unidirectional translocation of associated protons (with respect to the hemi-indigo scaffold) takes place during this cycle. Current efforts in our lab focus on immobilization of these N-heterocyclic hemi-indigos in porous materials and membranes, in addition to time-resolved spectroscopic studies. Furthermore, we envision that the selective inhibition of either forward or backward photoisomerization paths by binding of a substrate^44^ can be applied as a general approach for directional transport and pumping of that substrate. It is a tantalizing prospect, as it would give the ability to effectively generate concentration gradients in artificial compartmentalized systems by using broad-spectrum (solar) light as the energy source.

## Supporting Information Available

Experimental procedures, characterization of new compounds, ^1^H NMR and UV–vis studies, (TD)DFT calculations and X-ray analysis (CCDC 2234394–2234398) (PDF)

## References

[ref1] Luecke H.; Schobert B.; Richter H.-T.; Cartailler J.-P.; Lanyi J. K. Structural changes in bacteriorhodopsin during ion transport at 2 angstrom resolution. Science 1999, 286, 255–260. 10.1126/science.286.5438.255. 10514362

[ref2] Edman K.; Nollert P.; Royant A.; Belrhali H.; Pebay-Peyroula E.; Hajdu J.; Neutze R.; Landau E. M. High-resolution X-ray structure of an early intermediate in the bacteriorhodopsin photocycle. Nature 1999, 401, 822–826. 10.1038/44623. 10548112

[ref3] Subramaniam S.; Henderson R. Molecular mechanism of vectorial proton translocation by bacteriorhodopsin. Nature 2000, 406, 653–657. 10.1038/35020614. 10949309

[ref4] Steinberg-Yfrach G.; Liddell P. A.; Hung S.-C.; Moore A. L.; Gust D.; Moore T. A. Conversion of light energy to proton potential in liposomes by artificial photosynthetic reaction centres. Nature 1997, 385, 239–241. 10.1038/385239a0.

[ref5] Bhosale S.; Sisson A. L.; Talukdar P.; Fürstenberg A.; Banerji N.; Vauthey E.; Bollot G.; Mareda J.; Röger C.; Würthner F.; Sakai N.; Matile S.; et al. Photoproduction of proton gradients with π-stacked fluorophore scaffolds in lipid bilayers. Science 2006, 313, 84–86. 10.1126/science.1126524. 16825567

[ref6] Xie X.; Crespo G. A.; Mistlberger G.; Bakker E. Photocurrent generation based on a light-driven proton pump in an artificial liquid membrane. Nat. Chem. 2014, 6, 202–207. 10.1038/nchem.1858. 24557134

[ref7] Balzani V.; Credi A.; Raymo F. M.; Stoddart J. F. Artificial molecular machines. Angew. Chem., Int. Ed. 2000, 39, 3348–3391. 10.1002/1521-3773(20001002)39:19<3348::AID-ANIE3348>3.0.CO;2-X. 11091368

[ref8] Browne W. R.; Feringa B. L. Making molecular machines work. Nat. Nanotechnol. 2006, 1, 25–35. 10.1038/nnano.2006.45. 18654138

[ref9] Erbas-Cakmak S.; Leigh D. A.; McTernan C. T.; Nussbaumer A. L. Artificial molecular machines. Chem. Rev. 2015, 115, 10081–10206. 10.1021/acs.chemrev.5b00146. 26346838 PMC4585175

[ref10] Watson M. A.; Cockroft S. L. Man-made molecular machines: membrane bound. Chem. Soc. Rev. 2016, 45, 6118–6129. 10.1039/C5CS00874C. 26932423

[ref11] Aprahamian I. The future of molecular machines. ACS Cent. Sci. 2020, 6, 347–358. 10.1021/acscentsci.0c00064. 32232135 PMC7099591

[ref12] Li H.; Cheng C.; McGonigal P. R.; Fahrenbach A. C.; Frasconi M.; Liu W.-G.; Zhu Z.; Zhao Y.; Ke C.; Lei J.; Young R. M.; Dyar S. M.; Co D. T.; Yang Y.-W.; Botros Y. Y.; Goddard W. A.; Wasielewski M. R.; Astumian R. D.; Stoddart J. F. Relative unidirectional translation in an artificial molecular assembly fueled by light. J. Am. Chem. Soc. 2013, 135, 18609–18620. 10.1021/ja4094204. 24171644

[ref13] Ragazzon G.; Baroncini M.; Silvi S.; Venturi M.; Credi A. Light-Powered autonomous and directional molecular motion of a dissipative self-assembling system. Nat. Nanotechnol. 2015, 10, 70–75. 10.1038/nnano.2014.260. 25420035

[ref14] Canton M.; Groppi J.; Casimiro L.; Corra S.; Baroncini M.; Silvi S.; Credi A. Second-generation light-fueled supramolecular pump. J. Am. Chem. Soc. 2021, 143, 10890–10894. 10.1021/jacs.1c06027. 34282901 PMC8323096

[ref15] Cheng C.; McGonigal P. R.; Schneebeli S. T.; Li H.; Vermeulen N. A.; Ke C.; Stoddart J. F. An artificial molecular pump. Nat. Nanotechnol. 2015, 10, 547–553. 10.1038/nnano.2015.96. 25984834

[ref16] Amano S.; Fielden S. D. P.; Leigh D. A. A catalysis-driven artificial molecular pump. Nature 2021, 594, 529–534. 10.1038/s41586-021-03575-3. 34163057

[ref17] Qiu Y.; Feng Y.; Guo Q.-H.; Astumian R. D.; Stoddart J. F. Pumps through the ages. Chem. 2020, 6, 1952–1977. 10.1016/j.chempr.2020.07.009.

[ref18] Feng Y.; Ovalle M.; Seale J. S. W.; Lee C. K.; Kim D. J.; Astumian R. D.; Stoddart J. F. Molecular pumps and motors. J. Am. Chem. Soc. 2021, 143, 5569–5591. 10.1021/jacs.0c13388. 33830744

[ref19] Nakashima K.; Georgiev A.; Yordanov D.; Matsushima Y.; Hirashima S.-I.; Miura T.; Antonov L. Solvent-triggered long-range proton transport in 7-hydroxyquinoline using a sulfonamide transporter group. J. Org. Chem. 2022, 87, 6794–6806. 10.1021/acs.joc.2c00494. 35512011

[ref20] Pezzato C.; Cheng C.; Stoddart J. F.; Astumian R. D. Mastering the non-equilibrium assembly and operation of molecular machines. Chem. Soc. Rev. 2017, 46, 5491–5507. 10.1039/C7CS00068E. 28338143

[ref21] Aprahamian I.; Goldup S. M. Non-equilibrium steady states in catalysis, molecular motors, and supramolecular materials: why networks and language matter. J. Am. Chem. Soc. 2023, 145, 14169–14183. 10.1021/jacs.2c12665. 37343130 PMC10326876

[ref22] Serreli V.; Lee C.-F.; Kay E. R.; Leigh D. A. A molecular information ratchet. Nature 2007, 445, 523–527. 10.1038/nature05452. 17268466

[ref23] Petermayer C.; Thumser S.; Kink F.; Mayer P.; Dube H. Hemiindigo: highly bistable photoswitching at the biooptical window. J. Am. Chem. Soc. 2017, 139, 15060–15067. 10.1021/jacs.7b07531. 28944664

[ref24] Ikegami M.; Arai T. Photoisomerization and fluorescence properties of hemiindigo compounds having intramolecular hydrogen bonding. Bull. Chem. Soc. Jpn. 2003, 76, 1783–1792. 10.1246/bcsj.76.1783.

[ref25] Ikegami M.; Suzuki T.; Kaneko Y.; Arai T. Photochromism of hydrogen bonded compounds. Mol. Cryst. Liq. Cryst. Sci. Technol., Sect. A 2000, 345, 113–118. 10.1080/10587250008023904.

[ref26] Josef V.; Hampel F.; Dube H. Heterocyclic hemithioindigos: highly advantageous properties as molecular photoswitches. Angew. Chem., Int. Ed. 2022, 61, e202210855 10.1002/anie.202210855. PMC9826360 36040861

[ref27] Krell-Jørgensen M.; Zulfikri H.; Bonnevie M. G.; Bro F. S.; Dohn A. O.; Laraia L. Redshifted and thermally bistable one-way quantitative hemithioindigo-derived photoswitches enabled by isomer-specific excited state intramolecular proton transfer. Chem. Commun. 2023, 59, 563–566. 10.1039/D2CC05548A. 36537010

[ref28] Zweig J. E.; Newhouse T. R. Isomer-specific hydrogen bonding as a design principle for bidirectionally quantitative and redshifted hemithioindigo photoswitches. J. Am. Chem. Soc. 2017, 139, 10956–10959. 10.1021/jacs.7b04448. 28749144

[ref29] Chaur M. N.; Collado D.; Lehn J.-M. Configurational and constitutional information storage: multiple dynamics in systems based on pyridyl and acyl hydrazones. Chem. - Eur. J. 2011, 17, 248–258. 10.1002/chem.201002308. 21207621

[ref30] Landge S. M.; Aprahamian I. A pH activated configurational rotary switch: controlling the *E*/*Z* isomerization in hydrazones. J. Am. Chem. Soc. 2009, 131, 18269–18271. 10.1021/ja909149z. 19968272

[ref31] Nicholls-Allison E. C.; Nawn G.; Patrick B. O.; Hicks R. G. Protoisomerization of indigo di- and monoimines. Chem. Commun. 2015, 51, 12482–12485. 10.1039/C5CC04492H. 26146012

[ref32] Burger U.; Bringhen A. O. Cyclization studies With *N-Mannich* bases of 2-substituted indoles. Helv. Chim. Acta 1989, 72, 93–100. 10.1002/hlca.19890720112.

[ref33] Berg E. R.; Green D. D.; Moliva A. D. C.; Bjerke B. T.; Gealy M. W.; Ulness D. J. Ion-pair interaction in pyridinium carboxylate solutions. J. Phys. Chem. A 2008, 112, 833–838. 10.1021/jp076260l. 18189374

[ref34] Dolain C.; Maurizot V.; Huc I. Protonation-induced transition between two distinct helical conformations of a synthetic oligomer via a linear intermediate. Angew. Chem., Int. Ed. 2003, 42, 2738–2740. 10.1002/anie.200351080. 12820254

[ref35] Allen A. D.; Rosenbaum M.; Seto N. O. L.; Tidwell T. T. Addition of trifluoroacetic acid to substituted styrenes. J. Org. Chem. 1982, 47, 4234–4239. 10.1021/jo00143a011.

[ref36] Hao T.; Yang Y.; Liang W.; Fan C.; Wang X.; Wu W.; Chen X.; Fu H.; Chen H.; Yang C. Trace mild acid-catalysed *Z*→*E* isomerization of norbornene-fused stilbene derivatives: intelligent chiral molecular photoswitches with controllable self-recovery. Chem. Sci. 2021, 12, 2614–2622. 10.1039/D0SC05213B. PMC8179340 34164029

[ref37] Villarón D.; Duindam N.; Wezenberg S. J. Push-pull stiff-stilbene: proton-gated visible-light photoswitching and acid-catalyzed isomerization. Chem. - Eur. J. 2021, 27, 17346–17350. 10.1002/chem.202103052. 34605565 PMC9298359

[ref38] Sacherer M.; Hampel F.; Dube H. Diaryl-hemiindigos as visible light, pH, and heat responsive four-state switches and application in photochromic transparent polymers. Nat. Commun. 2023, 14, 4382 10.1038/s41467-023-39944-x. 37474507 PMC10359318

[ref39] Wiedbrauk S.; Dube H. Hemithioindigo—an emerging photoswitch. Tetrahedron Lett. 2015, 56, 4266–4274. 10.1016/j.tetlet.2015.05.022.

[ref40] Pina J.; Sarmento D.; Accoto M.; Gentili P. L.; Vaccaro L.; Galvão A.; Seixas de Melo J. S. Excited-state proton transfer in indigo. J. Phys. Chem. B 2017, 121, 2308–2318. 10.1021/acs.jpcb.6b11020. 28221047

[ref41] Yumoto K.; Irie M.; Matsuda K. Control of the photoreactivity of diarylethene derivatives by quaternarization of the pyridylethynyl group. Org. Lett. 2008, 10, 2051–2054. 10.1021/ol8005216. 18426221

[ref42] Hou I. C.-Y.; Berger F.; Narita A.; Müllen K.; Hecht S. Proton-gated ring-closure of a negative photochromic azulene-based diarylethene. Angew. Chem., Int. Ed. 2020, 59, 18532–18536. 10.1002/anie.202007989. PMC7589205 33439528

[ref43] Medved’ M.; Hoorens M. W. H.; Di Donato M.; Laurent A. D.; Fan J.; Taddei M.; Hilbers M.; Feringa B. L.; Buma W. J.; Szymanski W. Tailoring the optical and dynamic properties of iminothioindoxyl photoswitches through acidochromism. Chem. Sci. 2021, 12, 4588–4598. 10.1039/D0SC07000A. 34163724 PMC8179557

[ref44] Wezenberg S. J. Photoswitchable molecular tweezers: isomerization to control substrate binding, and what about *vice versa*?. Chem. Commun. 2022, 58, 11045–11058. 10.1039/D2CC04329G. PMC9531670 36106956

