# Supplementary material for: Monodirectional Photocycle Drives Proton Translocation
Source: J Am Chem Soc. 2023 Sep 15;145(38):21020–6. doi: 10.1021/jacs.3c06587 (PMC10540201; doi:10.1021/jacs.3c06587)
Supplement: Supplementary file 1 — ja3c06587_si_001.pdf [file ja3c06587_si_001.pdf]

# SUPPORTING INFORMATION

# Monodirectional Photocycle Drives Proton Translocation

Nol Duindam,[a] Michelle van Dongen,[a] Maxime A. Siegler,[b] and Sander J. Wezenberg[a*]

[a] *Leiden Institute of Chemistry, Leiden University,*

*Einsteinweg 55, 2333 CC Leiden, The Netherlands*

[b] *Department of Chemistry, Johns Hopkins University,*

*3400 N. Charles St., Baltimore, MD, 21218, United States*

Email: s.j.wezenberg@lic.leidenuniv.nl

## Table of Contents

1. Experimental section ........ S2

2. $^{1}$H and $^{13}$C NMR spectra of compounds ........ S11

3. Single crystal X-ray crystallography ........ S25

4. $^{1}$H NMR titrations with TFA ........ S38

5. $^{1}$H NMR studies of acid/base controlled isomerization ........ S40

6. Fitting of $^{1}$H NMR isomerization data to first-order decay kinetics ........ S57

7. UV-Vis studies of acid/base controlled isomerization ........ S66

8. UV-Vis photoisomerization studies ........ S72

9. $^{1}$H NMR photoisomerization studies ........ S87

10. Quantum yield determination ........ S89

11. Computational details ........ S103

12. References ........ S121

# 1. Experimental section

## General methods and materials

Toluene was dried using a Pure Solve 400 solvent purification system from Innovative Technology. $CDCl_3$ was purchased from Eurisotop, which was filtered over basic $Al_2O_3$ (to remove DCl). The degassing of the solvents was carried out by purging with $N_2$ for 15 min, unless noted otherwise. Aldehydes **8**[1] and **9**[2] were synthesized as describe elsewhere. All other chemicals were commercially available and were used without further purification. Flash chromatography (FC) was performed using silica gel ($SiO_2$) purchased from Screening Devices BV (60 M, 0.04-0.063 mm). Thin-layer chromatography (TLC) was carried out on aluminum sheets coated with silica 60 F254. Compounds were visualized with UV light (254 nm or 365 nm). $^{1}H$ and $^{13}C$ NMR spectra were recorded on Bruker AV-I-400, Bruker AV-IV 400, Bruker AV-IV- 500 Ultra Shield, and Bruker AV-III-600 instruments at 294 K unless indicated otherwise. Chemical shifts ($\delta$) are denoted in parts per million (ppm) relative to residual protiated solvent ($CDCl_3$: for $^{1}H$ detection, $\delta$ = 7.26 ppm; for $^{13}C$ detection, $\delta$ = 77.16 ppm). The splitting pattern of peaks is designated as follows: s (singlet), d (doublet), t (triplet), q (quartet), p (quintet), h (septet), m (multiplet), br (broad). High-resolution mass spectrometry (ESI-MS) was performed on a Thermo Scientific Q Exactive HF spectrometer with ESI ionization. IR spectra were recorded on a Perkin Elmer Spectrum Two FT-IR spectrometer. The intensity of bands ($\nu$ = $cm^{-1}$) is assigned as follows: s (strong), m (medium), w (weak), very w (very weak), br (broad), and sh (shoulder). Melting points were determined with a Büchi M560 apparatus. UV-Vis spectra were recorded on an Agilent Cary 8454 spectrometer equipped with a stirrer (250 rpm) and a temperature control unit (293 K unless noted otherwise) using 1 cm or 1 mm quartz cuvettes. A Newport 20CGA–360 KI162 Longpass filter was placed in front of the light source of the spectrophotometer to absorb all incident light below 360 nm. Irradiation of UV-Vis and NMR samples was carried out using LEDs purchased from Thorlabs, i.e. mounted fiber-coupled LEDs M365F1 (3.0 mW), M385FP1 (18 mW), M405F1 (3.0 mW), M455F3 LEDs (17 mW), unmounted epoxy-encased LED465E (20 mW), LED525E (2.5 mW), LED591E (2 mW), LED630E (7.2 mW), and unmounted LED with glass lens LED660L (13 mW).

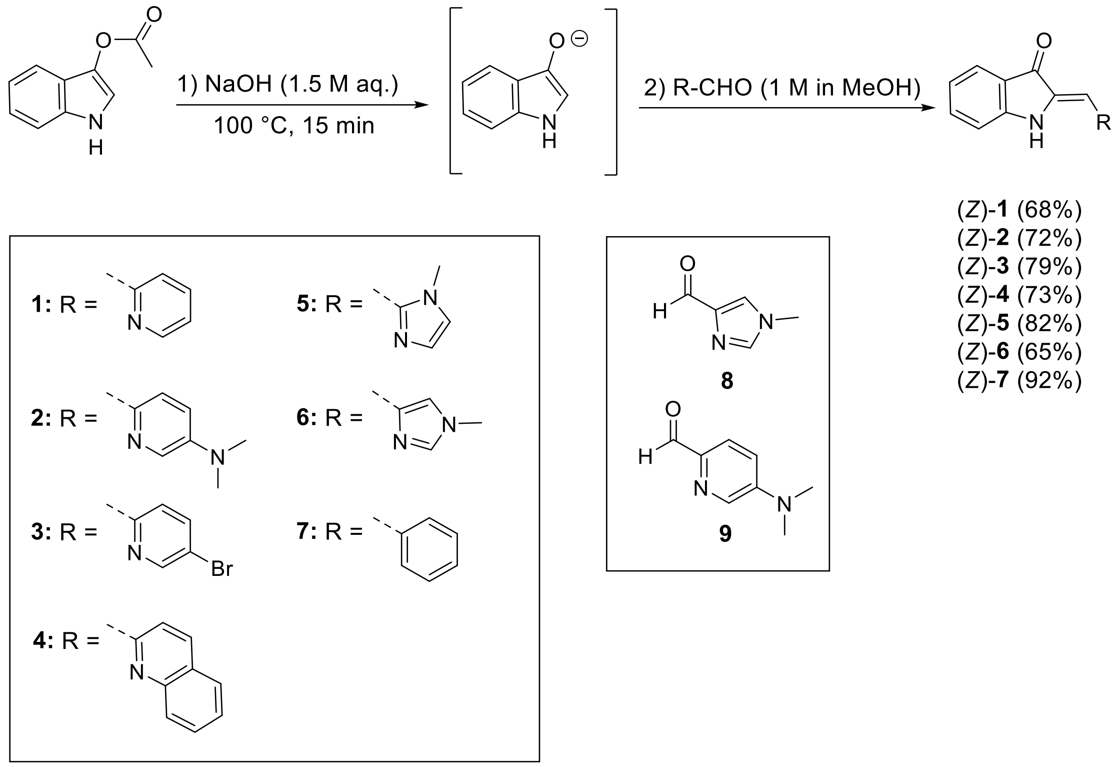


**Scheme S1.** Synthetic route toward hemi-indigos **1-7**. A similar procedure to access structurally related hemi-indigo derivatives was described by the group of Burger,[3] and more recently applied by the group of Dube.[4] Non-commercial, known aldehydes **8**[1] and **9**[2] are also depicted.

### General procedure for the synthesis of hemi-indigo dyes 1-7

Based on previously reported literature procedures,[3,4] 1*H*-indol-3-yl acetate (50 mg, 0.29 mmol) was suspended in 1.6 mL degassed 1.5 M aqueous NaOH solution (purged with $N_2$ for 30 min or subjected to 3 freeze-pump-thaw cycles) under Ar atmosphere. The mixture was heated to 100 °C for 15 min, after which the resulting greenish solution was cooled to 0 °C and the respective aldehyde (0.29 mmol) in MeOH (0.3 mL) was added. The reaction mixture was stirred at room temperature for 3–5 days at rt, diluted with 5 mL water, and extracted with EtOAc (3 × 15 mL). The combined organic layers were dried over $Na_2SO_4$ and concentrated. Purification by flash chromatography (FC) yielded the desired hemi-indigo product.

**(*Z*)-2-(pyridin-2-ylmethylene)indolin-3-one [(*Z*)-1]**

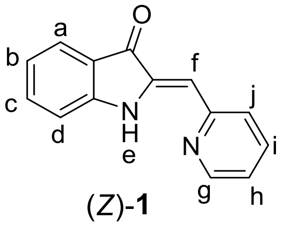


(*Z*)-**1**

1*H*-indol-3-yl acetate (50 mg, 0.29 mmol) and picolinaldehyde (21 μL, 0.29 mmol) were reacted as described in the general method for 3 days. Purification by FC ($SiO_2$; EtOAc/ pentane 1:18) yielded (*Z*)-**1** as red solid (43 mg, 68%); $R_f$ = 0.17 ($SiO_2$; EtOAc/pentane 1:18); m.p. 149.0–151.3 °C ([5]: 153–154 °C); $^1$H NMR (500 MHz, $CDCl_3$, assignments based on 2D COSY and NOESY spectra) $\delta$ = 9.95 (s, 1H; H**e**), 8.67 (d, *J* = 4.9 Hz, 1H; H**g**), 7.71–7.66 (m, 2H; H**a** and H**i**), 7.45 (ddd, *J* = 8.1, 7.2, 1.3 Hz, 1H; H**c**), 7.39 (m, 1H; H**j**), 7.14 (ddd, *J* = 7.5, 4.9, 1.2 Hz, 1H; H**h**), 6.97 (dt, *J* = 8.0, 0.8 Hz, 1H; H**d**), 6.90 (td, *J* = 7.4, 0.8 Hz, 1H; H**b**), 6.61 (s, 1H; H**f**); $^{13}$C NMR (126 MHz, $CDCl_3$) $\delta$ = 187.9, 156.1, 153.3, 149.4, 138.4, 136.7, 136.6, 126.3, 125.2, 121.6, 120.9, 120.2, 111.7, 105.3; IR (ATR) $\nu$ = 3353-3328 (br. w), 2998-2850 (w), 1690 (m), 1629 (w, sh), 1598 (m), 1584 (m), 1482 (m),1460 (m), 1437 (m), 1380 (m), 1358 (w, sh), 1275 (m), 1261 (w, sh), 887 (w), 850 (w), 763 (s, sh), 747 (very s); HRMS (ESI) *m/z:* 223.0864 ($[M+H]^+$, calcd for $C_{14}H_{11}N_2O^+$: 223.0866). The spectroscopic data is in agreement with the literature.[5]

**(*Z*)-2-{[5-(dimethylamino)pyridin-2-yl]methylene}indolin-3-one [(*Z*)-2]**

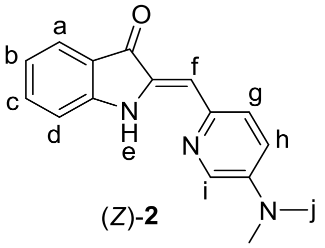

(*Z*)-**2**

1*H*-indol-3-yl acetate (50 mg, 0.29 mmol) was reacted with 5-(dimethylamino)picolinaldehyde (45 mg, 0.29 mmol) as described in the general procedure for 5 days, with the exception that $CH_2Cl_2$ was used in the extraction. Purification by FC ($SiO_2$; $CH_2Cl_2$ to EtOAc/$CH_2Cl_2$ 1:9) yielded (*Z*)-**2** as dark-red crystalline solid (54 mg, 72%); $R_f$ = 0.54 ($SiO_2$; EtOAc/$CH_2Cl_2$ 1:9); m.p. 258.9–261.2 °C; $^1$H NMR (500 MHz, $CDCl_3$, assignments based on 2D COSY and NOESY spectra) $\delta$ = 9.76 (s, 1H; H**e**), 8.23 (d, *J* = 3.1 Hz, 1H; H**i),** 7.70 (d, *J* = 7.7 Hz, 1H; H**a**), 7.41 (ddd, *J* = 8.2, 7.2, 1.3 Hz, 1H; H**c**), 7.28 (d, *J* = 8.7 Hz, 1H; H**g**), 6.98 – 6.91 (m, 2H; H**d** and H**h),** 6.86 (m, 1H; H**b**), 6.66 (s, 1H; H**f**), 3.06 (s, 6H; H**j);** $^{13}$C NMR; (126 MHz, $CDCl_3$) $\delta$ = 187.3, 152.8, 144.5, 143.5, 135.9, 135.8, 134.7, 127.3, 124.9, 121.3, 119.4, 118.4, 111.5, 107.7, 40.1; IR (ATR) $\nu$ = 3294 (br. w), 2992 (w), 2912 (w), 1676 (m), 1623 (m), 1602 (m), 1593 (m), 1565 (m), 1545 (m), 1486 (m), 1466 (w), 1376 (m), 1306 (m), 1280 (s), 1264 (m), 1227 (m), 1148 (m), 1124 (m), 1099 (m), 962 (w), 870 (w), 861 (w), 829 (w), 767.(s, sh), 751 (s), 714 (s), 631 (m); HRMS (ESI) *m/z:* 266.1287 ([M+H]$^+$, calcd for $C_{16}H_{16}N_3O^+$: 266.1288).

**(*Z*)-2-[(5-bromopyridin-2-yl)methylene]indolin-3-one [(*Z*)-3]**

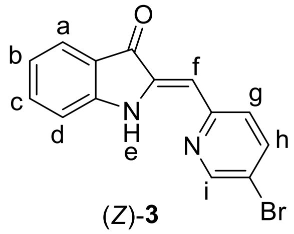


(*Z*)-**3**

1*H*-indol-3yl acetate (50 mg, 0.29 mmol) was reacted with 5-bromopicolinaldehyde (55 mg, 0.29 mmol) as described in the general procedure for 5 days. Purification by FC ($SiO_2$; EtOAc/Pet. Ether 0:1 to 2:8) yielded (*Z*)-**3** as dark red solid (68 mg, 79%); $R_f$ = 0.19 ($SiO_2$; EtOAc/Pet. Ether 1:9); m.p. 228.9–230.1 °C; $^1$H NMR (500 MHz, $CDCl_3$, assignments based on 2D COSY and NOESY spectra) $\delta$ = 9.65 (s, 1H; H**e**), 8.69 (d, *J* = 2.4 Hz, 1H; H**i**), 7.78 (dd, *J* = 8.4, 2.4 Hz, 1H; H**h**), 7.68 (d, *J* = 7.6, 1H; H**a**), 7.45 (ddd, *J* = 8.5, 7.3, 1.3 Hz, 1H; H**c**), 7.25 (d, *J* = 7.6 Hz, 1H; H**g**), 6.97 (dt, *J* = 8.1, 0.6 Hz, 1H; H**d**), 6.91 (td, *J* = 7.6, 0.8 Hz, 1H; H**b**), 6.52 (s, 1H; H**f**); $^{13}$C NMR (126 MHz, $CDCl_3$) $\delta$ = 187.7, 154.5, 153.1, 150.5, 139.3, 138.5, 136.7, 127.1, 125.3, 120.8, 120.5, 118.3, 111.7, 103.9; IR (ATR) $\nu$ = 3394 (w), 2919 (br. m), 2860 (w), 1693 (m), 1632 (m), 1608 (m), 1591 (sh), 1568 (w), 1486 (m), 1462 (m), 1401 (m), 1355 (m), 1317 (w, sh), 1306 (m), 1290 (m, sh), 1220 (s), 1196 (m, sh), 1162 (w), 1145 (m), 1130 (m), 1088 (br. m), 1009 (m), 966 (w), 873 (m), 861 (m), 851 (m, sh), 829 (m) 757 (w), 712 (m); HRMS (ESI) *m/z:* 300.9971 ($[M+H]^+$, calcd for $C_{14}H_{10}BrN_2O^+$: 300.9971).

**(*Z*)-2-(quinolin-2-ylmethylene)indolin-3-one [(*Z*)-4]**

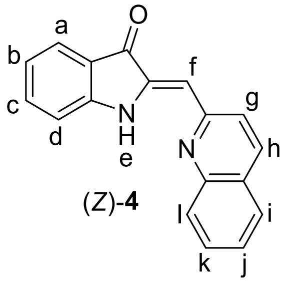

(*Z*)-**4**

1*H*-indol-3yl acetate (50 mg, 0.29 mmol) was reacted with quinoline-2-carbaldehyde (45 mg, 0.29 mmol) as described in the general procedure for 3 days. Purification by FC ($SiO_2$; EtOAc/Pet. Ether 1:9) yielded (*Z*)-**4** as bright red solid (56 mg, 73%); $R_f$ = 0.16 ($SiO_2$; EtOAc/Pet. Ether 1:9); m.p. 155.1–155.7 °C; $^1$H NMR; (500 MHz, $CDCl_3$, assignments based on 2D COSY and NOESY spectra) $\delta$ = 10.47 (s, 1H; H**e**), 8.11–8.03 (m, 2H; H**l** and H**h**), 7.77 (dd, $J$ = 8.2, 1.4 Hz, 1H; H**i**), 7.74–7.68 (m, 2H; H**a** and H**k**), 7.49 (m, 2H; H**c** and H**j**), 7.44 (d, $J$ = 8.5 Hz, 1H; H**g**), 7.02 (dt, $J$ = 8.0, 0.8 Hz, 1H; H**d**), 6.93 (td, $J$ = 7.4, 0.8 Hz, 1H; H**b**), 6.70 (s, 1H; H**f**); $^{13}$C NMR; (126 MHz, $CDCl_3$) $\delta$ = 188.0, 156.4, 153.3, 148.1, 139.3, 136.7, 136.4, 130.1, 128.9, 127.8, 126.7 (2), 125.3, 124.2, 120.9, 120.5, 111.8, 104.7; IR (ATR) $\nu$ = 3326 (br. w), 3058 (w), 2927 (w), 2857 (w), 1702 (m, sh), 1691 (m), 1640 (m), 1622 (m), 1590 (s), 1487 (m), 1470 (br. m), 1402 (m), 1374 (m), 1314 (m), 1291 (w), 1219 (m), 1204 (m, sh), 1137 (m), 1119 (w), 1097 (w), 849 (m), 820 (m), 754 (w), 740 (m), 709 (m), 617 (w); HRMS (ESI) *m/z:* 273.1020 ($[M+H]^+$, calcd for $C_{18}H_{13}N_2O^+$: 273.1022).

**(*Z*)-2-[(1-methyl-1*H*-imidazol-2-yl)methylene]indolin-3-one [(*Z*)-5]**

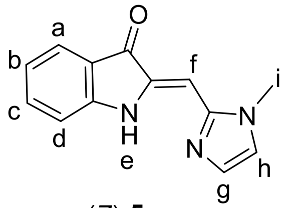


(*Z*)-**5**

1*H*-indol-3-yl acetate (50 mg, 0.29 mmol) and 1-methyl-1*H*-imidazole-2-carbaldehyde (31 mg, 0.29 mmol) were reacted as described in the general procedure for 3 days. Purification by FC ($SiO_2$; EtOAc/pentane 2:3) yielded (*Z*)-**5** as orange solid (53 mg, 82%); $R_f$ = 0.32 ($SiO_2$; EtOAc/pentane 2:3); m.p. 201.1–202.3 °C; $^1$H NMR; (500 MHz, $CDCl_3$, assignments based on 2D COSY and NOESY spectra) $\delta$ = 9.59 (s, 1H; H**e**), 7.68 (d, *J* = 7.6 Hz, 1H; H**a**), 7.43 (ddd, *J* = 8.1, 7.2, 1.3 Hz, 1H; H**c**), 7.23 (d, *J* = 1.1 Hz, 1H; H**g**), 6.96 (dt, *J* = 8.1, 0.8 Hz, 1H; H**d**), 6.94 (d, *J* = 1.2 Hz, 1H; H**h**), 6.89 (ddd, *J* = 7.8, 7.3, 0.8 Hz, 1H; H**b**), 6.51 (d, *J* = 0.5 Hz, 1H; H**f**), 3.75 (s, 3H; **i**); $^{13}$C NMR (126 MHz, $CDCl_3$) $\delta$ = 187.1, 153.6, 146.0, 137.4, 136.4, 130.1, 125.1, 122.3, 121.3, 120.1, 111.8, 92.2, 33.1; IR (ATR) $\nu$ = 3325 (m), 3132 (w), 3115 (w), 2929 (br. m), 2858 (m), 1687 (m), 1627 (m), 1586 (s), 1476 (m), 1445 (m), 1350 (m), 1312 (m), 1292 (m, sh), 1281 (s), 1263(m, sh), 1223 (m), 1192 (m), 1174 (m), 1145 (m), 1119 (m), 1097 (m), 1081 (m), 1046 (m), 968 (w), 927 (w), 890 (w), 831 (w), 792 (m), 752 (s), 722 (m), 709 (m), 677 (w), 624 (w); HRMS (ESI) *m/z:* 226.0972 ($[M+H]^+$, calcd for $C_{13}H_{12}N_3O^+$: 226.0975).

**(*Z*)-2-[(1-methyl-1*H*-imidazol-4-yl)methylene]indolin-3-one [(*Z*)-6]**

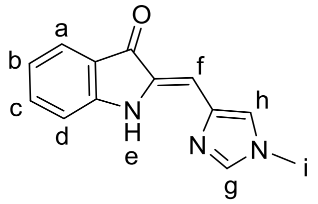


(*Z*)-**6**

1*H*-indol-3-yl acetate (50 mg, 0.29 mmol) was reacted with aldehyde **8** (40 mg, 0.36 mmol) as described in the general procedure for 3 days. Purification by FC ($SiO_2$; EtOAc/pentane 2:3 to EtOAc/pentane 4:1) yielded (*Z*)-**6** as yellow solid (42 mg, 65%); $R_f$ = 0.15 ($SiO_2$; EtOAc/ pentane 3:2); m.p. 166.4–166.7 °C; $^1$H NMR (500 MHz, $CDCl_3$, assignments based on 2D COSY and NOESY spectra) $\delta$ = 9.14 (s, 1H; H**e**), 7.68 (d, *J* = 7.7 Hz, 1H; H**a**), 7.51 (s, 1H; H**g**), 7.40 (ddd, *J* = 8.3, 7.2, 1.3 Hz, 1H; H**c**), 7.12 (d, *J* = 0.9 Hz, 1H; H**h**), 6.94 (dt, *J* = 8.1, 0.9 Hz, 1H; H**d**), 6.84 (ddd, *J* = 7.8, 7.2, 0.8 Hz, 1H; H**b**), 6.65 (s, 1H; H**f**), 3.72 (s, 3H; H**i**); $^{13}$C NMR (126 MHz, $CDCl_3$) $\delta$ = 186.8, 152.7, 139.5, 138.7, 135.8, 135.4, 124.8, 122.8, 121.4, 119.2, 111.5, 101.6, 33.8; IR (ATR) $\nu$ = 3352 (m), 3129 (w), 2997 (m), 2921 (m), 1687 (m), 1637 (m), 1606 (m), 1588 (m, sh), 1547 (m), 1484 (m), 1465 (m), 1377 (m), 1334 (m), 1312 (m), 1280 (m), 1264 (m), 1241 (m), 1129 (m), 1098 (m), 988 (w), 824 (w), 768.40 (s, sh) 755 (s), 753.64 (s), 740 (s, sh), 704 (m), 614 (m); HRMS (ESI) *m/z:* 226.0972 ($[M+H]^+$, calcd for $C_{13}H_{12}N_3O^+$: 226.0975).

**(*Z*)-2-benzylideneindolin-3-one [(*Z*)-7]**

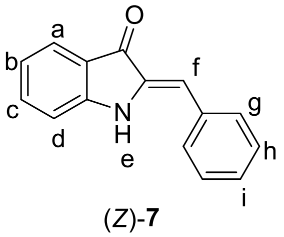


(*Z*)-**7**

1*H*-indol-3-yl acetate (50 mg, 0.29 mmol) was reacted with benzaldehyde (29 µL, 0.29 mmol) as described in the general procedure for 3 days. Purification by FC ($SiO_2$; EtOAc/Pet. Ether 1:5) yielded **7** as a red solid (58 mg, 92%); $^1$H NMR (500 MHz, $CDCl_3$, assignments based on 2D COSY and NOESY spectra) $\delta$ = 7.76 (d, $J$ = 7.7 Hz, 1H, H**a**), 7.56 (d, $J$ = 7.4 Hz, 2H, **g**), 7.52 – 7.42 (m, 3H, H**c** and H**h**), 7.38–7.30 (m, 1H, H**i**), 7.02–6.95 (m, 1H, H**b** and H**d**), 6.88 (s, 1H, H**f**), 6.82 (s, 1H, H**e**); $^{13}$C NMR (126 MHz, $CDCl_3$) $\delta$ = 186.7, 153.3, 136.3, 135.5, 134.9, 129.6, 129.4, 128.7, 125.2, 122.0, 120.9, 112.1, 111.7. Spectral data is in accordance with the literature,[6] except for the chemical shift of H**e** ($\delta$ = 6.82 ppm, [6]: 7.05 ppm), which presumably is dependent on concentration (5.4 mM in our case) and water content.

## 2. $^{1}$H and $^{13}$C NMR spectra of compounds

### (*Z*)-2-(pyridin-2-ylmethylene)indolin-3-one [(*Z*)- 1]

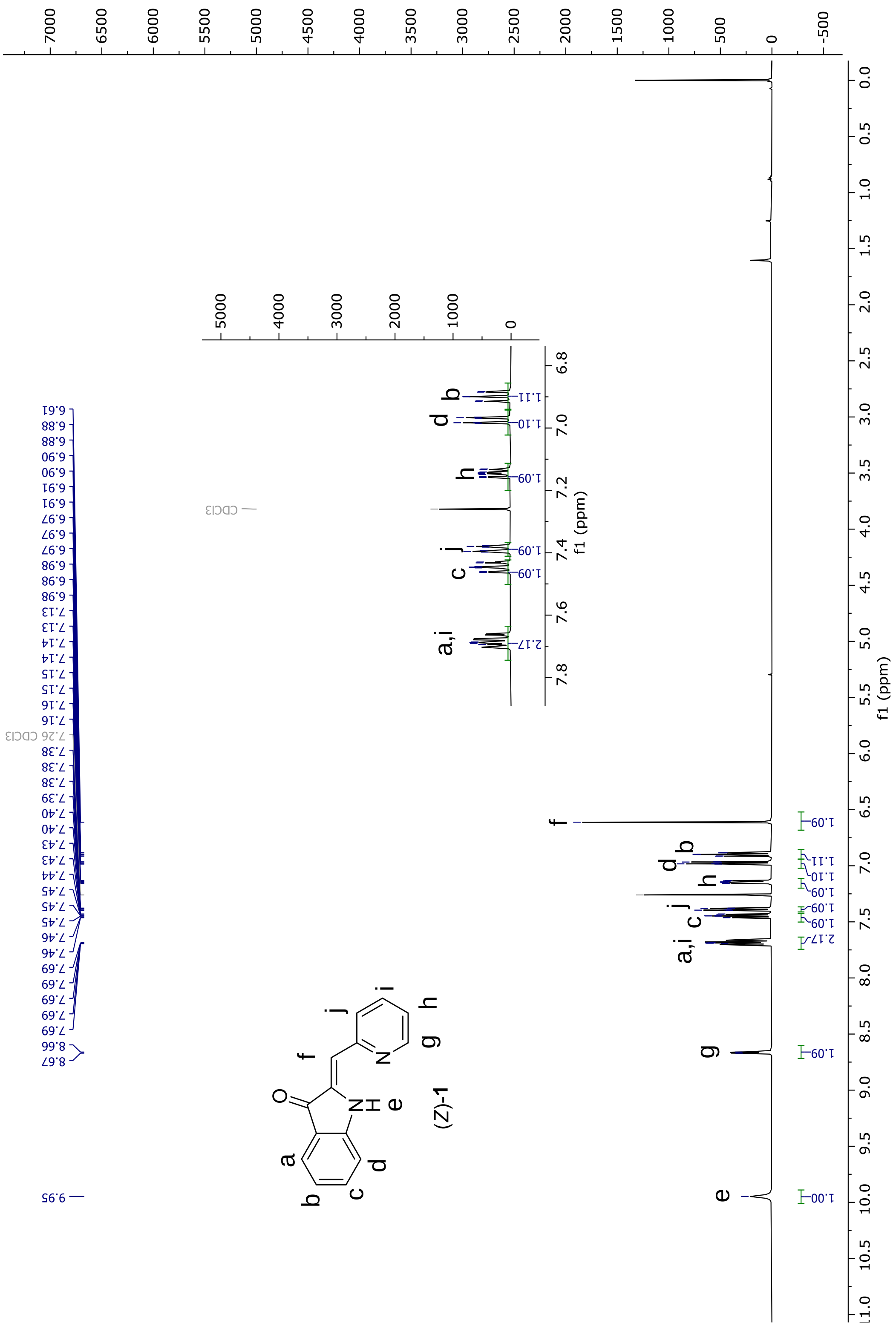


**Figure S1.** $^{1}$H NMR spectrum (500 MHz, $CDCl_3$) recorded at 294 K of (*Z*)-**1**.

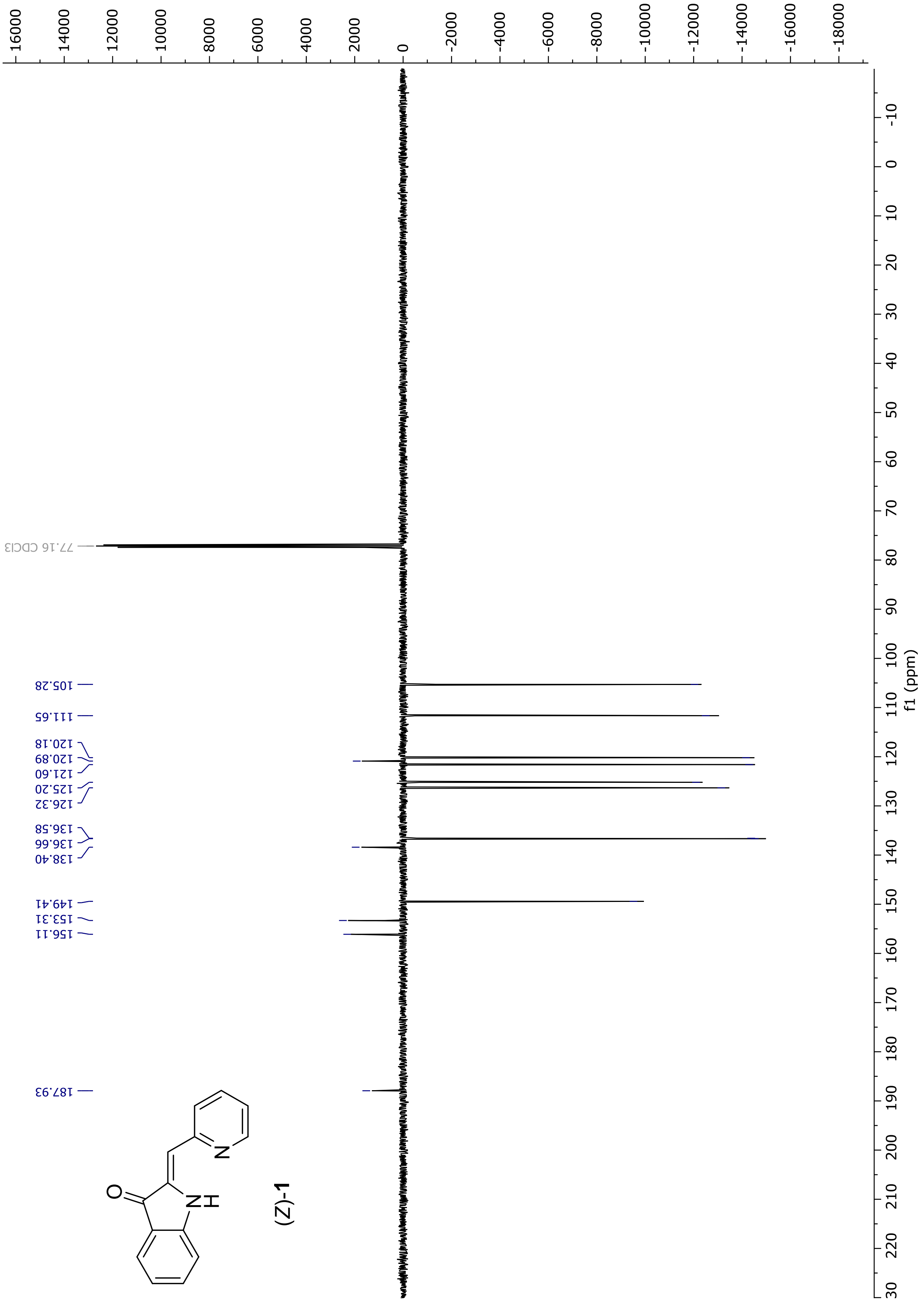


**Figure S2.** $^{13}$C NMR spectrum (APT, 126 MHz, $CDCl_3$) recorded at 294 K of (*Z*)-**1**.

**(*Z*)-2-{[5-(dimethylamino)pyridin-2-yl]methylene}indolin-3-one [(*Z*)-2]**

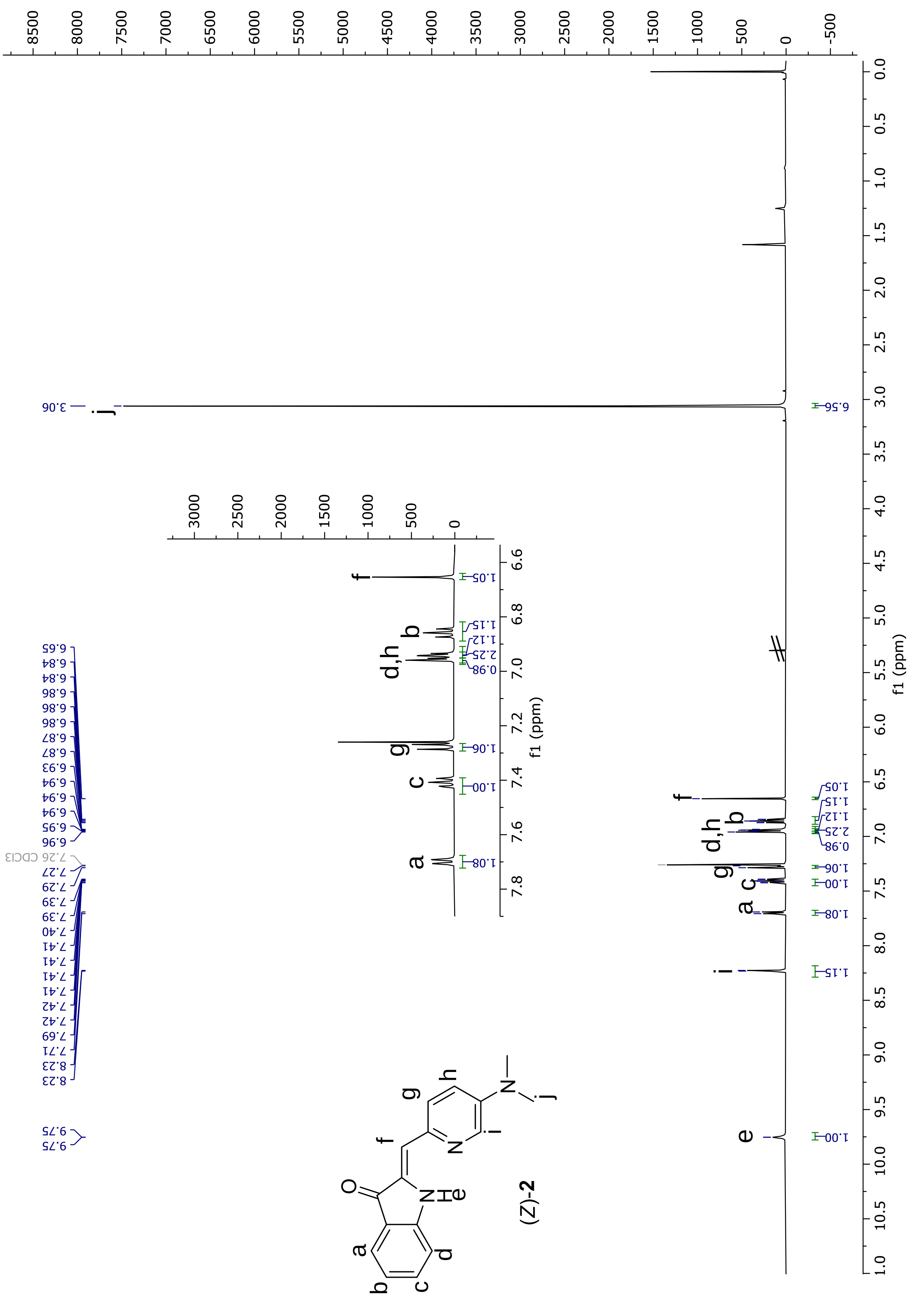


**Figure S3.** $^{1}$H NMR spectrum (500 MHz, $CDCl_3$) recorded at 294 K of (*Z*)-**2**.

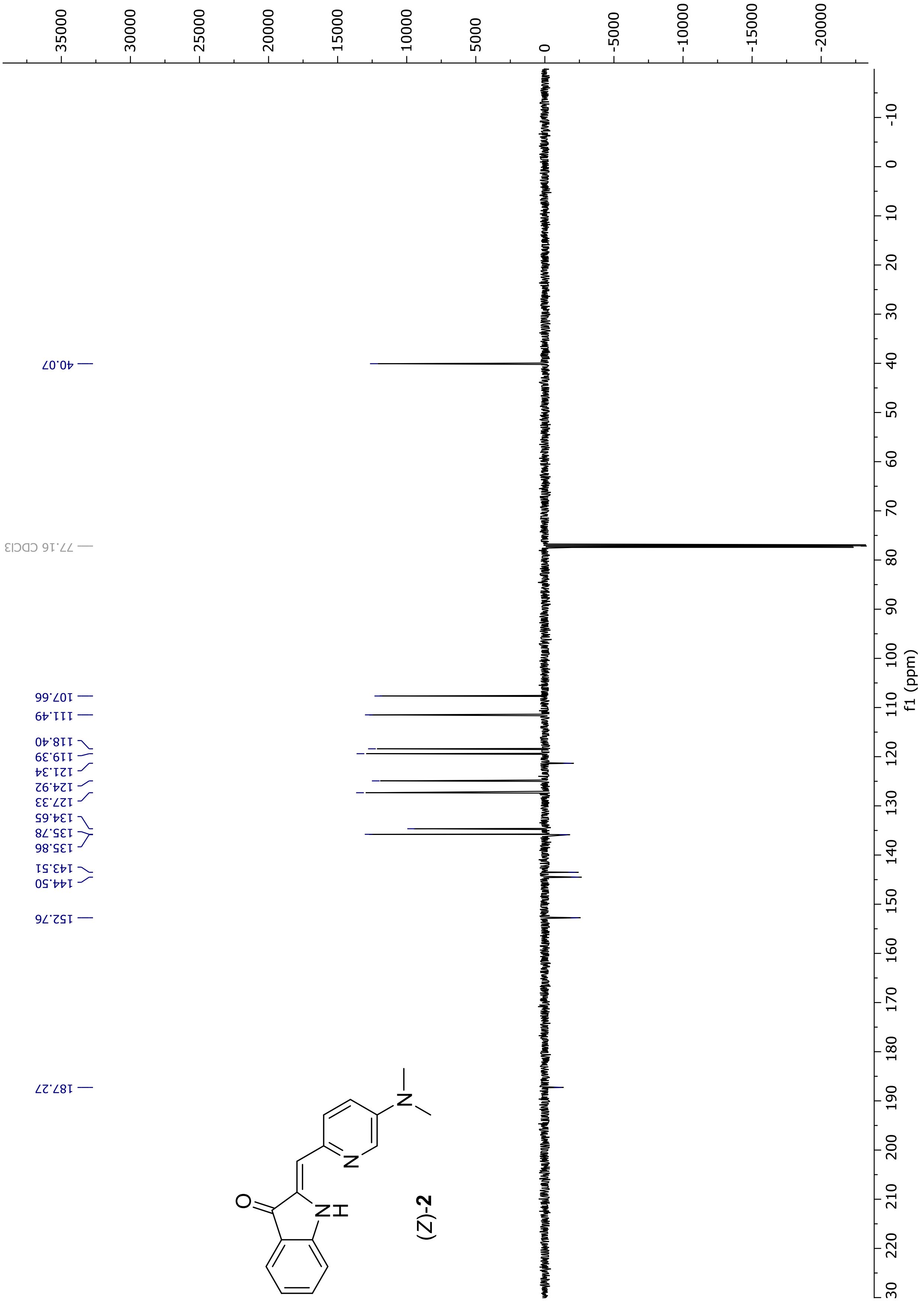


**Figure S4.** $^{13}$C NMR spectrum (APT, 126 MHz, $CDCl_3$) recorded at 294 K of (*Z*)-**2**.

**(*Z*)-2-[(5-bromopyridin-2-yl)methylene]indolin-3-one [(*Z*)-3]**

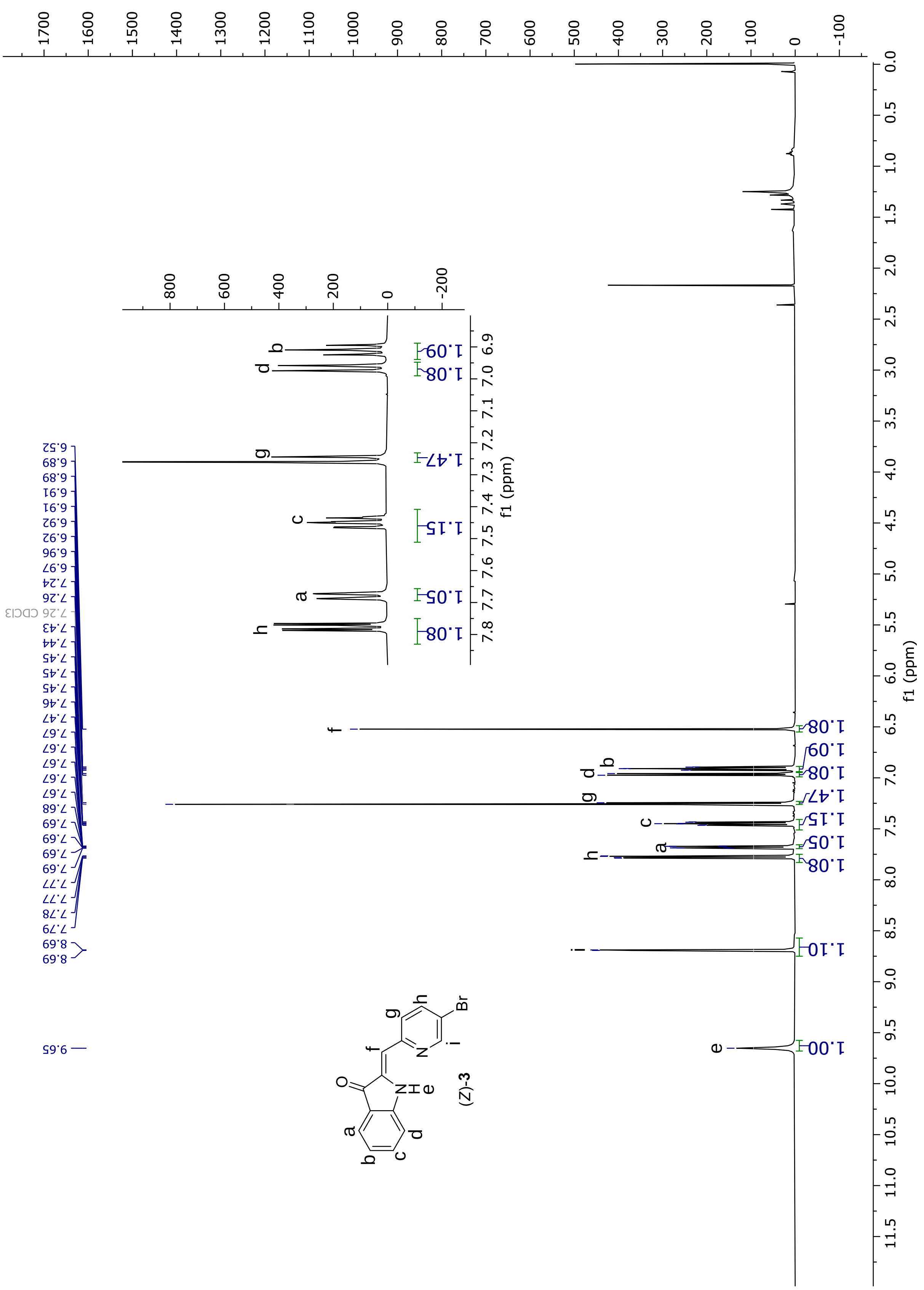


**Figure S5.** $^{1}$H NMR spectrum (500 MHz, $CDCl_3$) recorded at 294 K of (*Z*)-**3**.

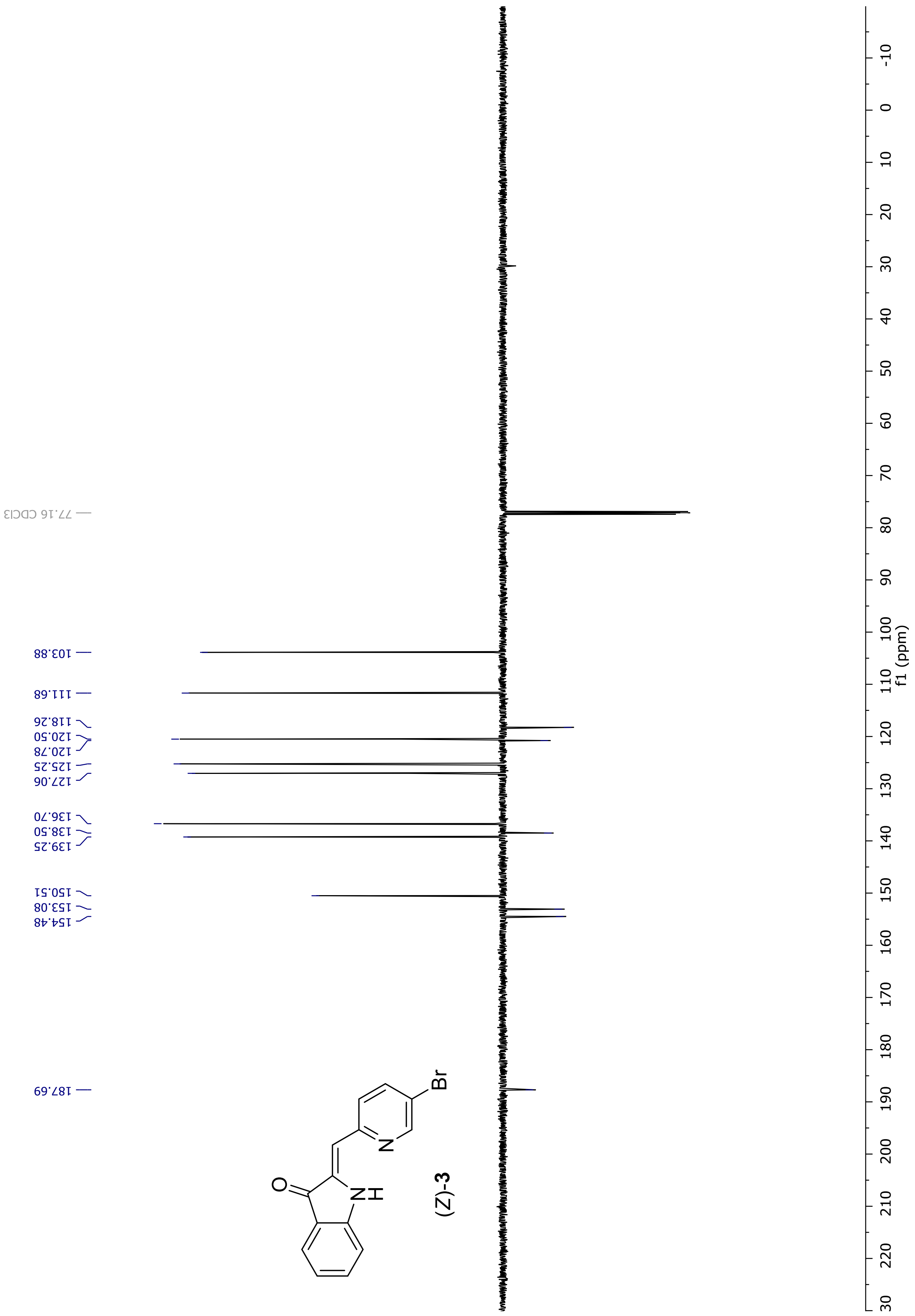


**Figure S6.** $^{13}$C NMR spectrum (APT, 126 MHz, $CDCl_3$) recorded at 294 K of (*Z*)-**3**.

**(*Z*)-2-(quinolin-2-ylmethylene)indolin-3-one [(*Z*)-4]**

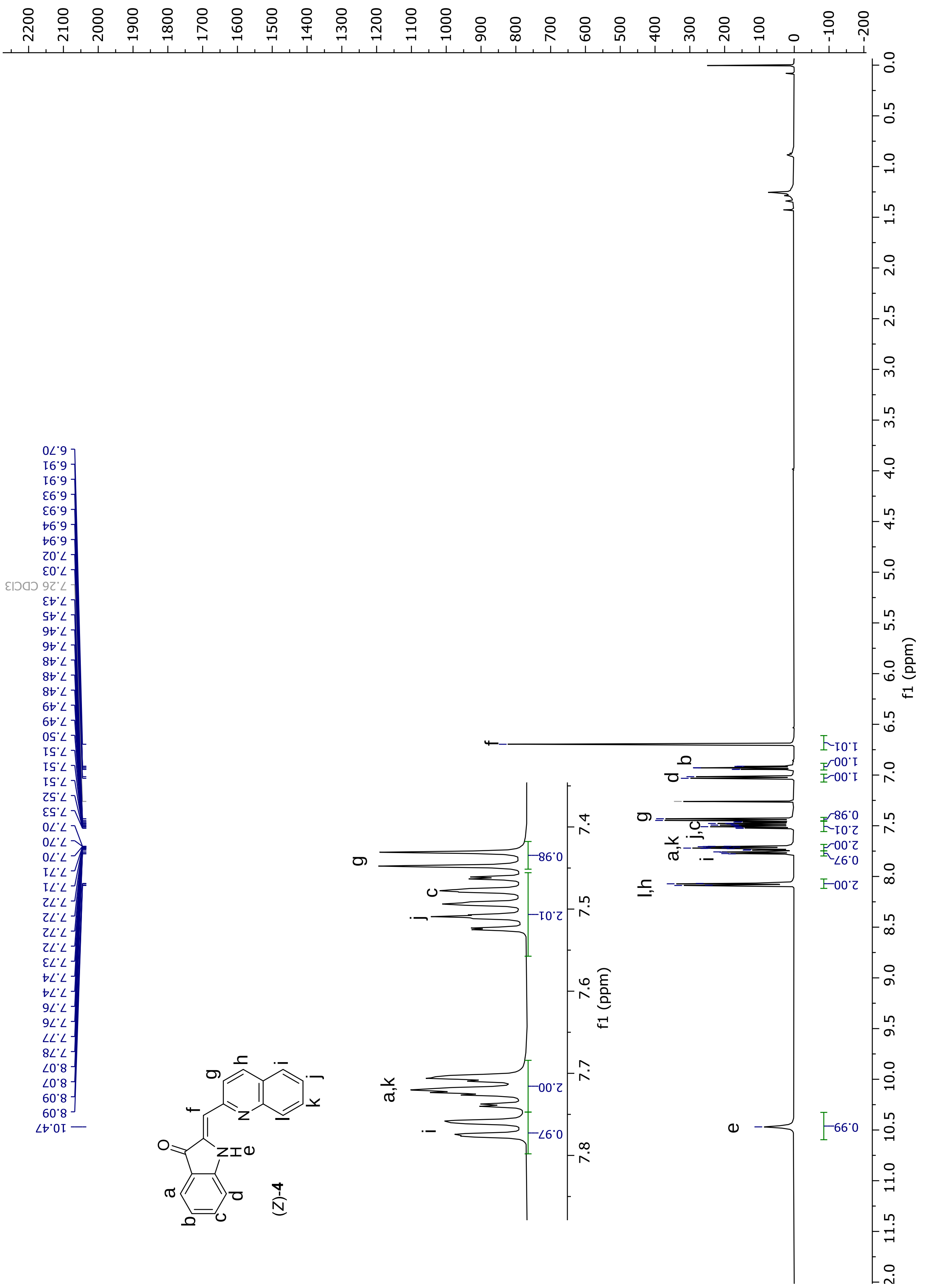

**Figure S7.** $^{1}$H NMR spectrum (500 MHz, $CDCl_3$) recorded at 294 K of (Z)-**4**.

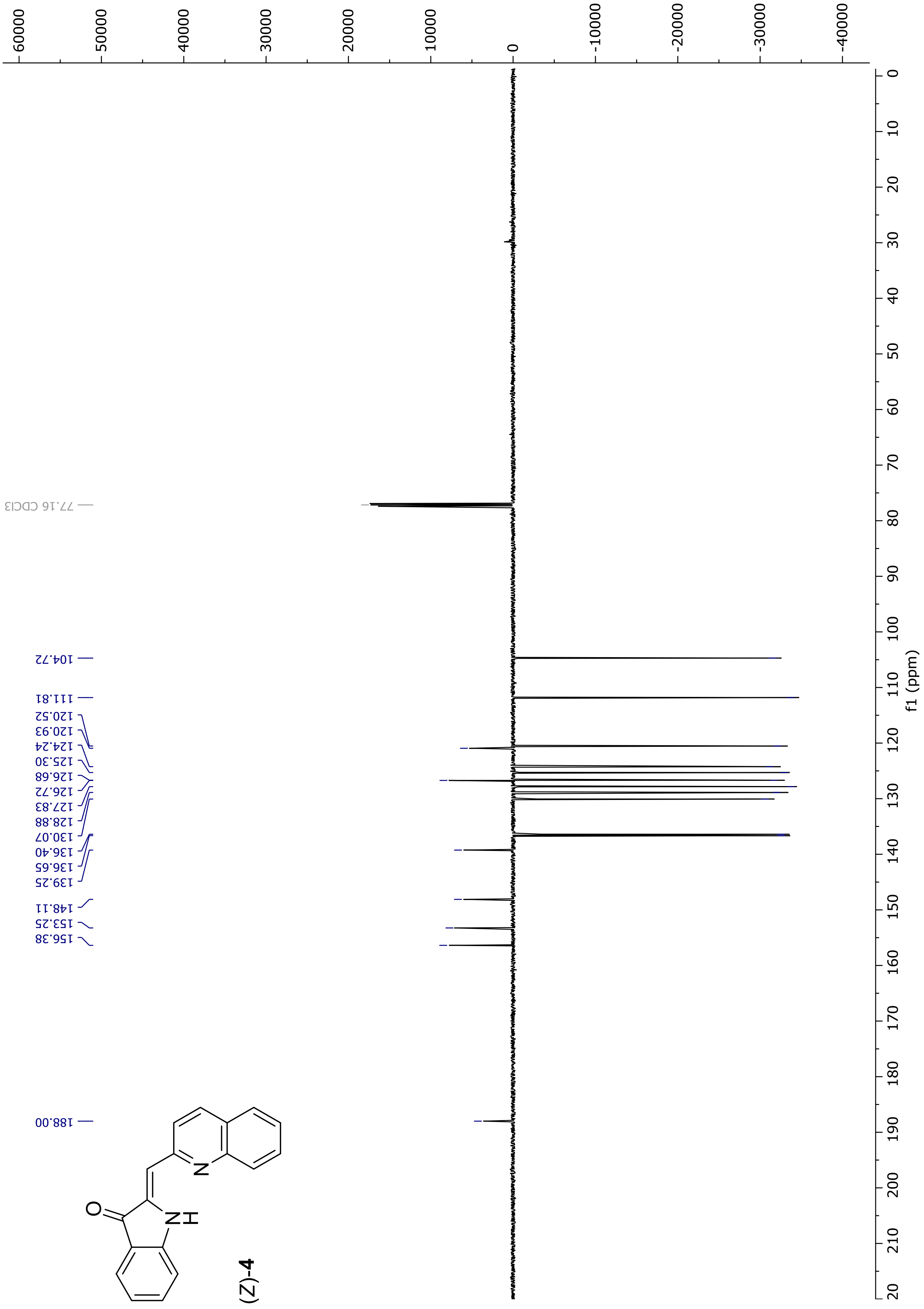


**Figure S8.** $^{13}C$ NMR spectrum (APT, 126 MHz, $CDCl_3$) recorded at 294 K of (*Z*)-**4**.

**(*Z*)-2-[(1-methyl-1*H*-imidazol-2-yl)methylene]indolin-3-one [(*Z*)-5]**

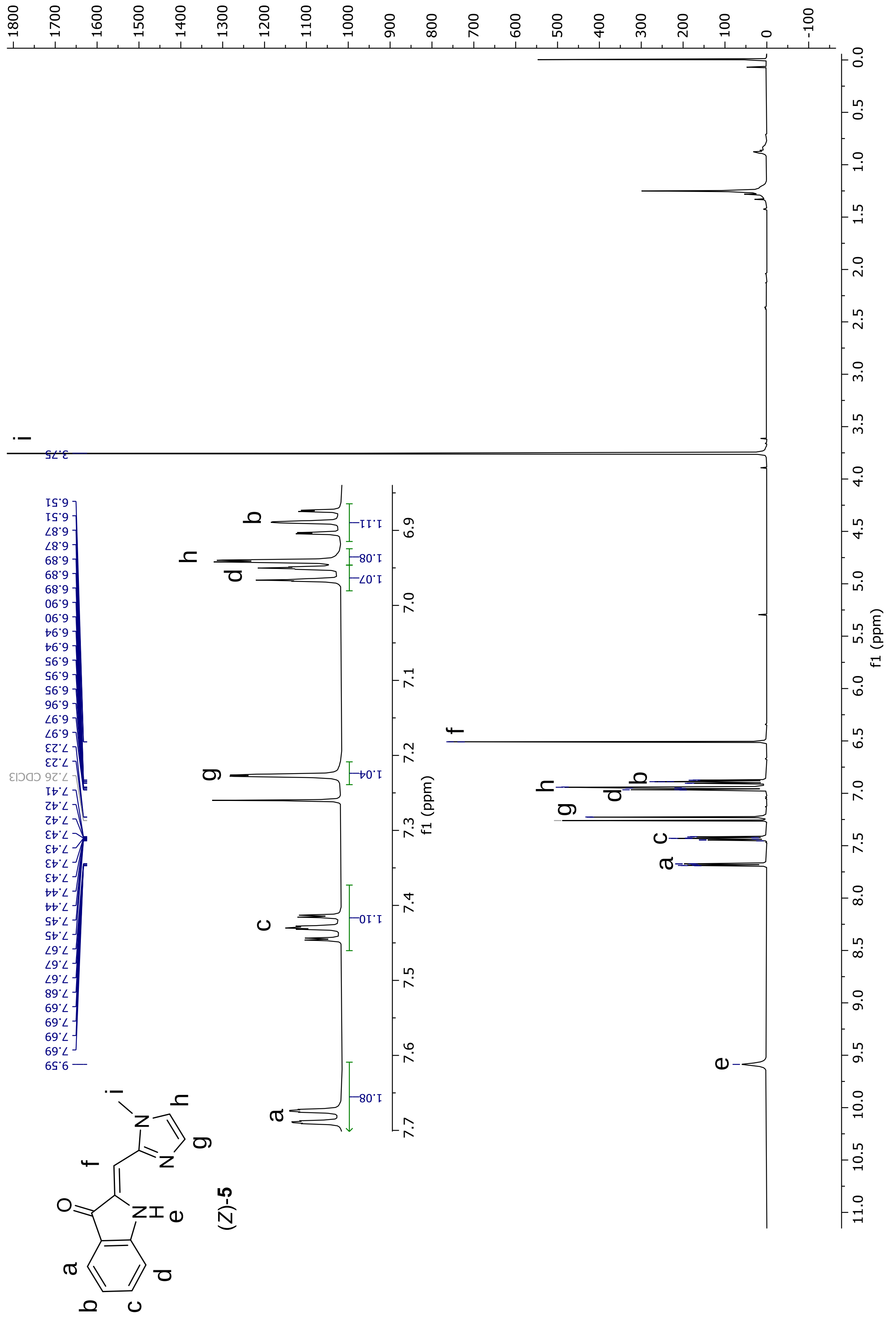


**Figure S9.** $^{1}$H NMR spectrum (500 MHz, $CDCl_3$) recorded at 294 K of (*Z*)-**5**.

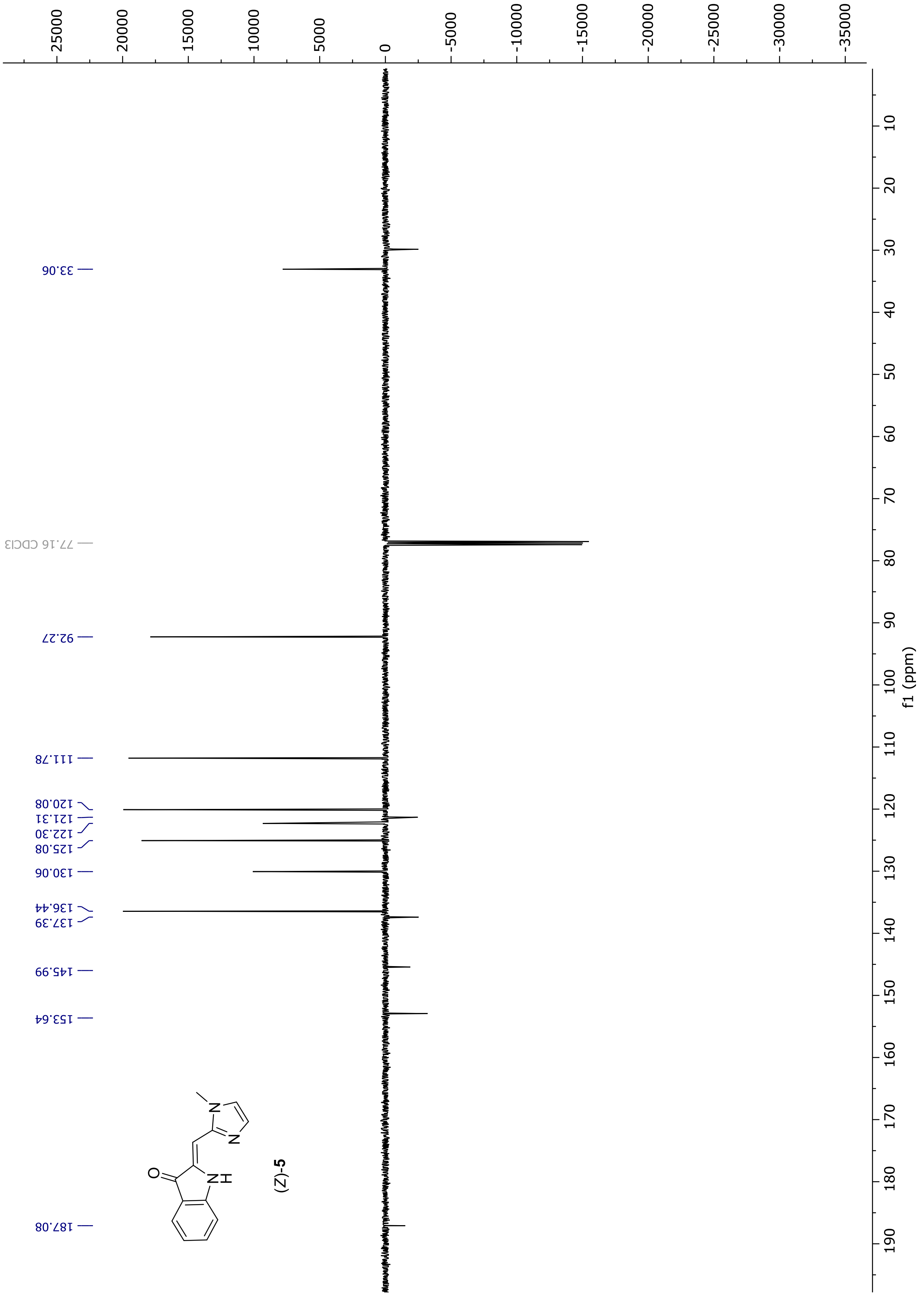


**Figure S10.** $^{13}$C NMR spectrum (APT, 126 MHz, $CDCl_3$) recorded at 294 K of (*Z*)-**5**.

**(*Z*)-2-[(1-methyl-1*H*-imidazol-4-yl)methylene]indolin-3-one [(*Z*)-6]**

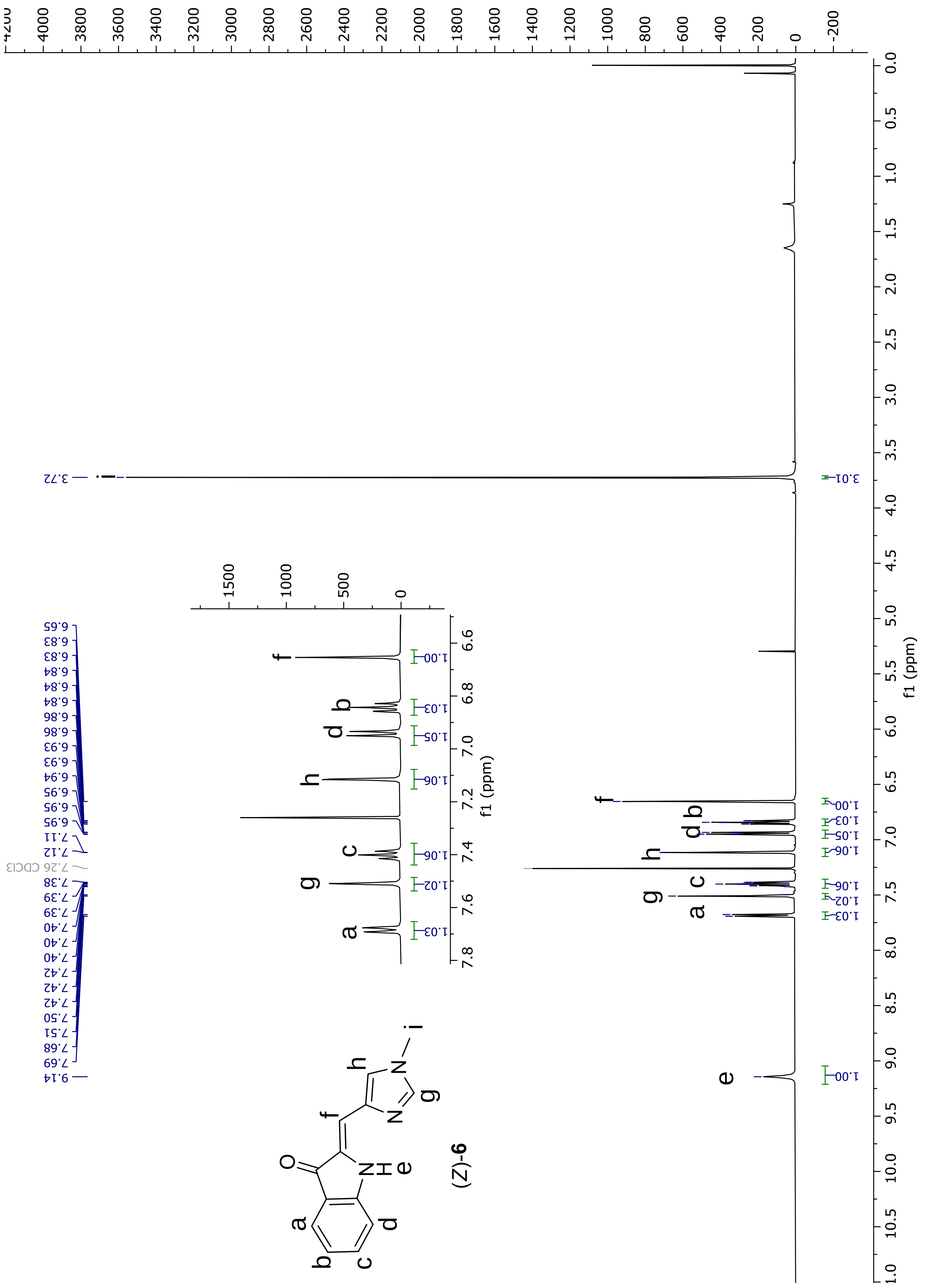


**Figure S11.** $^{1}$H NMR spectrum (500 MHz, $CDCl_3$) recorded at 294 K of (*Z*)-**6**.

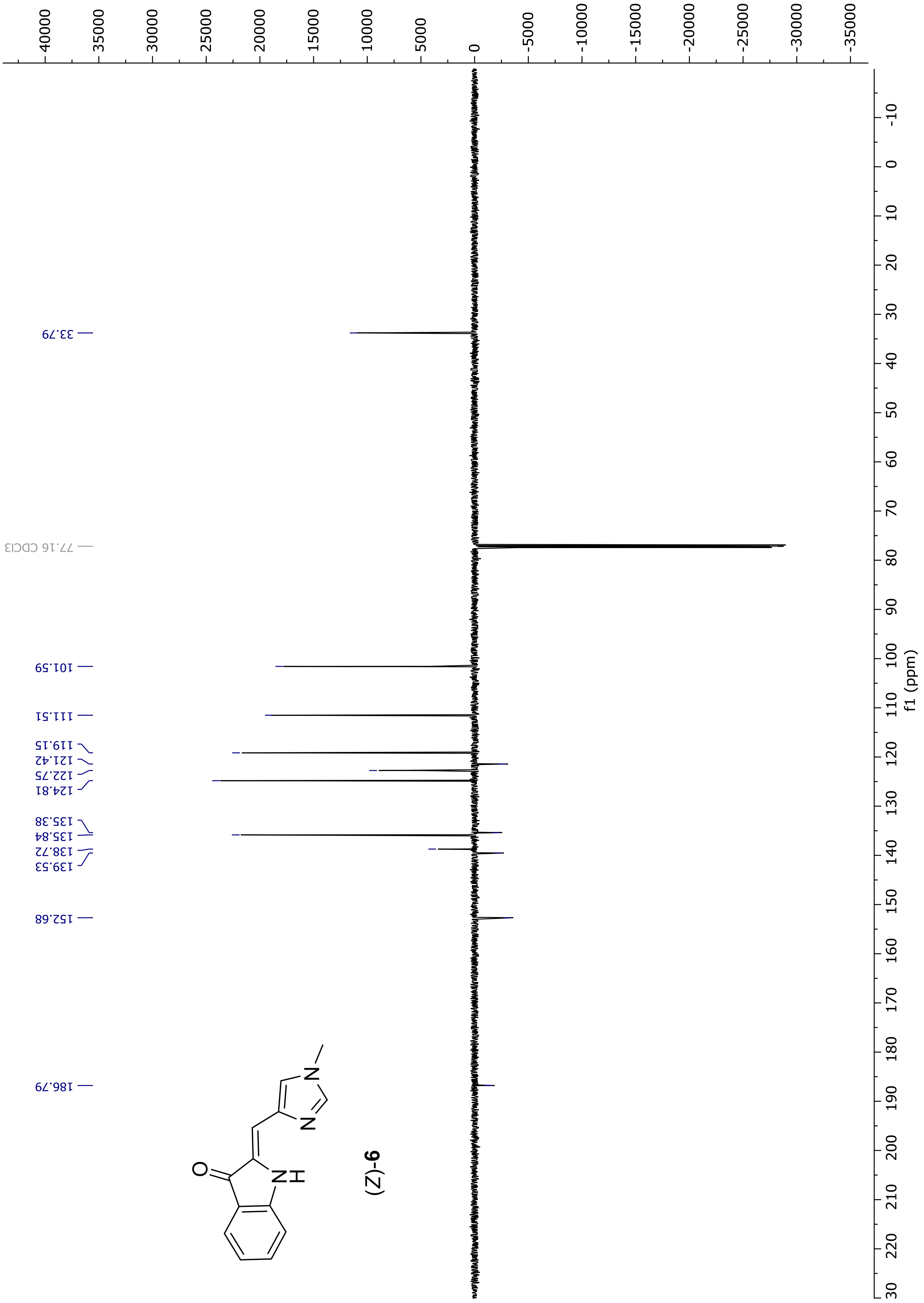


**Figure S12.** $^{13}$C NMR spectrum (APT, 126 MHz, $CDCl_3$) recorded at 294 K of (*Z*)-**6**.

**(*Z*)-2-benzylideneindolin-3-one [(*Z*)-7]**

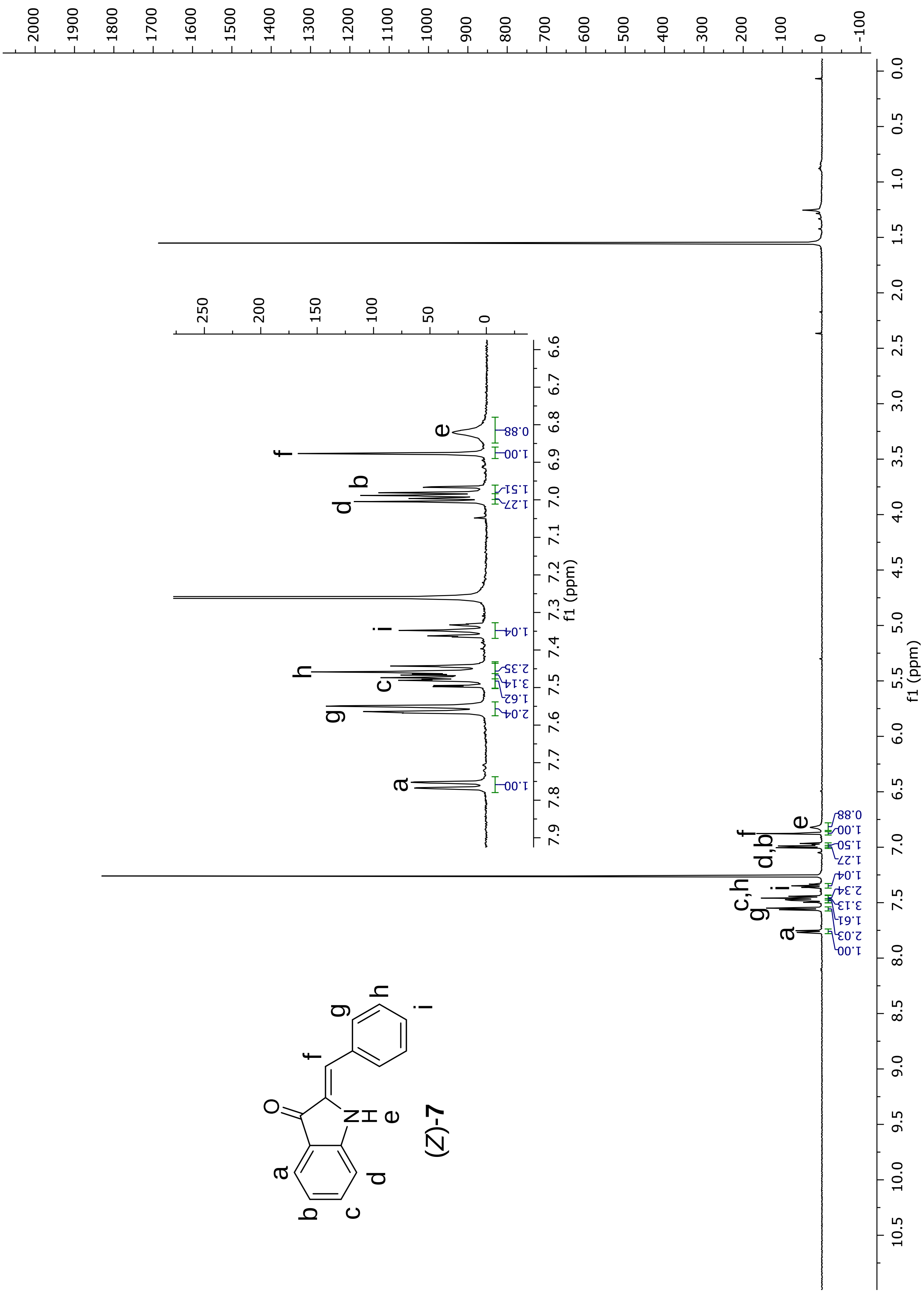


**Figure S13.** $^{1}$H NMR spectrum (500 MHz, $CDCl_3$) recorded at 294 K of (*Z*)-**7** (5.4 mM).

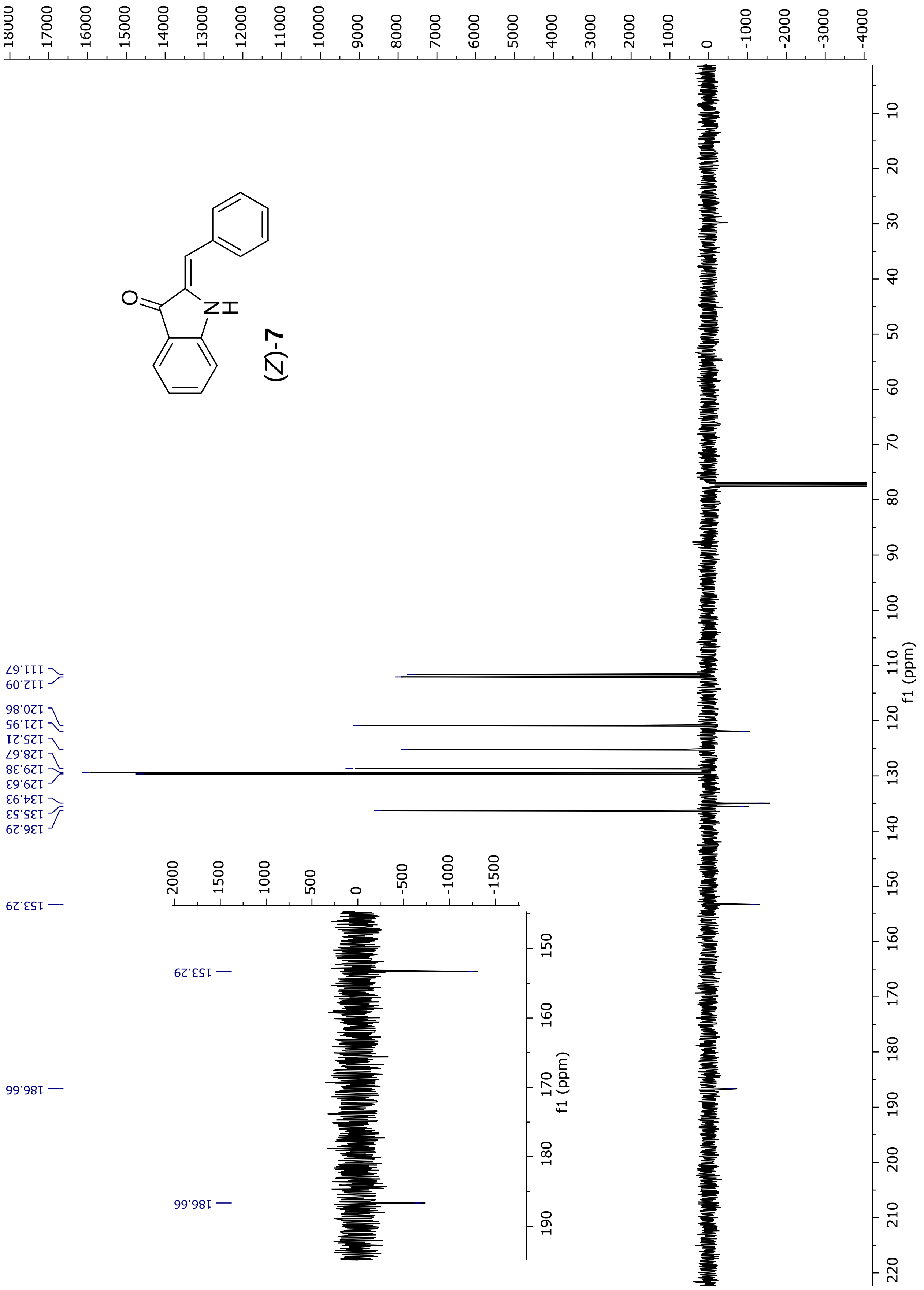


**Figure S14.** $^{13}$C NMR spectrum (APT, 126 MHz, $CDCl_3$) recorded at 294 K of (*Z*)-**7**.

## 3. Single crystal X-ray crystallography

Computer programs used: *CrysAlis PRO* 1.171.39.29c (Rigaku OD, 2017), *SHELXS2018*/3 (Sheldrick, 2018), *SHELXL2018*/3 (Sheldrick, 2018), *SHELXTL* v6.10 (Sheldrick, 2008).[7]

### Crystal structure of (*Z*)-1

Single crystals were grown from a solution in MeCN.

All reflection intensities were measured at 110(2) K using a SuperNova diffractometer (equipped with Atlas detector) with Mo *K*α radiation ($\lambda$ = 0.71073 Å) under the program CrysAlisPro (Version CrysAlisPro 1.171.39.29c, Rigaku OD, 2017). The same program was used to refine the cell dimensions and for data reduction. The structure was solved with the program SHELXS-2018/3 (Sheldrick, 2018) and was refined on $F^2$ with SHELXL-2018/3 (Sheldrick, 2018). Numerical absorption correction based on gaussian integration over a multifaceted crystal model was applied using CrysAlisPro. The temperature of the data collection was controlled using the system Cryojet (manufactured by Oxford Instruments). The H atoms were placed at calculated positions (unless otherwise specified) using the instruction AFIX 43 with the isotropic displacement parameters having values 1.2 $U_{eq}$ of the attached C atoms. The H atom attached to N7 was found from difference Fourier map, and its coordinates and isotropic temperature factor were refined freely.

The structure is ordered.

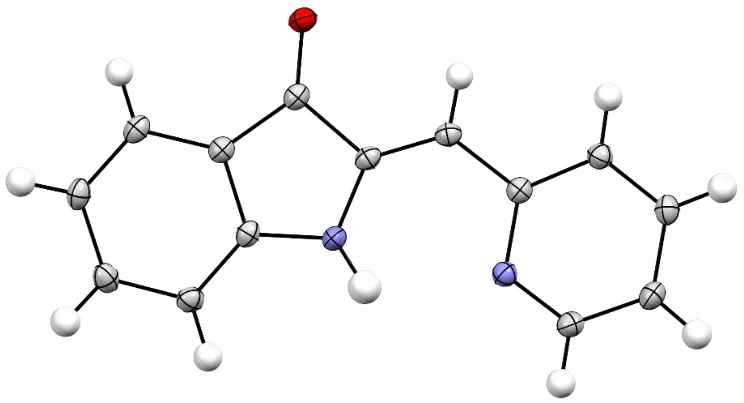

**Figure S15.** Displacement ellipsoid plot (50% probability level) of (*Z*)-**1** at 110(2) K.

**Table S1.** Crystallographic data for the structure of (*Z*)-**1**.

| | (*Z*)-**1** |
|---|---|
| Crystal data | |
| Chemical formula | $C_{14}H_{10}N_2O$ |
| $M_r$ | 222.24 |
| Crystal system, space group | Monoclinic, $P2_1/n$ |
| Temperature (K) | 110 |
| *a*, *b*, *c* (Å) | 7.0582 (2), 14.0070 (4), 10.7964 (4) |
| β (°) | 101.108 (3) |
| *V* (Å$^3$) | 1047.38 (6) |
| *Z* | 4 |
| Radiation type | Mo *K*α |
| μ (mm$^{-1}$) | 0.09 |
| Crystal size (mm) | 0.48 × 0.37 × 0.34 |
| | |
| Data collection | |
| Diffractometer | SuperNova, Dual, Cu at zero, Atlas |
| Absorption correction | Gaussian<br>*CrysAlis PRO* 1.171.41.93a (Rigaku Oxford Diffraction, 2020) Numerical absorption correction based on gaussian integration over a multifaceted crystal model Empirical absorption correction using spherical harmonics, implemented in SCALE3 ABSPACK scaling algorithm. |
| $T_{min}$, $T_{max}$ | 0.241, 1.000 |
| No. of measured, independent and observed [$I > 2\sigma(I)$] reflections | 19717, 2396, 2187 |
| $R_{int}$ | 0.030 |
| ((sin θ/λ)$_{max}$ (Å$^{-1}$) | 0.650 |
| | |
| Refinement | |
| $R[F^2 > 2\sigma(F^2)]$, $wR(F^2)$, $S$ | 0.037, 0.100, 1.05 |
| No. of reflections | 2396 |
| No. of parameters | 159 |
| H-atom treatment | H atoms treated by a mixture of independent and constrained refinement |
| $\Delta\rho_{max}$, $\Delta\rho_{min}$ (e Å$^{-3}$) | 0.37, -0.20 |

**Crystal structure of (*Z*)-2**

Single crystals were grown from a solution in a mixture of $CH_2Cl_2$/MeCN.

All reflection intensities were measured at 110(2) K using a SuperNova diffractometer (equipped with Atlas detector) with Mo *K*α radiation ($\lambda$ = 0.71073 Å) under the program CrysAlisPro (Version CrysAlisPro 1.171.39.29c, Rigaku OD, 2017). The same program was used to refine the cell dimensions and for data reduction. The structure was solved with the program SHELXS-2018/3 (Sheldrick, 2018) and was refined on $F^2$ with SHELXL-2018/3 (Sheldrick, 2018). Numerical absorption correction based on gaussian integration over a multifaceted crystal model was applied using CrysAlisPro. The temperature of the data collection was controlled using the system Cryojet (manufactured by Oxford Instruments). The H atoms were placed at calculated positions (unless otherwise specified) using the instructions AFIX 43 or AFIX 137 with isotropic displacement parameters having values 1.2 or 1.5 $U_{eq}$ of the attached C atoms. The H atom attached to N7 was found from difference Fourier map, and its coordinates and isotropic temperature factor were refined freely.

The structure is ordered.

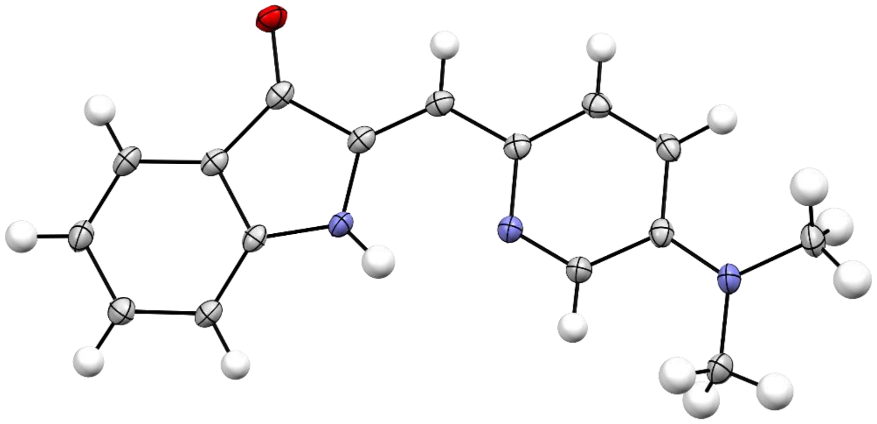

**Figure S16.** Displacement ellipsoid plot (50% probability level) of (*Z*)-**2** at 110(2) K.

**Table S2.** Crystallographic data for the structure of (*Z*)-**2**.

| | (*Z*)-**2** |
|---|---|
| Crystal data | |
| Chemical formula | $C_{16}H_{15}N_3O$ |
| $M_r$ | 265.31 |
| Crystal system, space group | Monoclinic, $P2_1/n$ |
| Temperature (K) | 110 |
| *a*, *b*, *c* (Å) | 10.1732 (5), 12.9706 (5), 10.5863 (5) |
| β (°) | 111.973 (6) |
| *V* (Å$^3$) | 1295.42 (11) |
| *Z* | 4 |
| Radiation type | Mo *K*α |
| μ (mm$^{-1}$) | 0.09 |
| Crystal size (mm) | 0.23 × 0.20 × 0.06 |
| | |
| Data collection | |
| Diffractometer | SuperNova, Dual, Cu at zero, Atlas |
| Absorption correction | Gaussian<br>*CrysAlis PRO* 1.171.41.93a (Rigaku Oxford Diffraction, 2020) Numerical absorption correction based on gaussian integration over a multifaceted crystal model Empirical absorption correction using spherical harmonics, implemented in SCALE3 ABSPACK scaling algorithm. |
| $T_{min}$, $T_{max}$ | 0.666, 1.000 |
| No. of measured, independent and observed [$I > 2\sigma(I)$] reflections | 18366, 2980, 2502 |
| $R_{int}$ | 0.031 |

| | |
|---|---|
| $(\sin \theta/\lambda)_{max}$ (Å$^{-1}$) | 0.650 |
| | |
| Refinement | |
| $R[F^2 > 2\sigma(F^2)]$, $wR(F^2)$, $S$ | 0.041, 0.108, 1.05 |
| No. of reflections | 2980 |
| No. of parameters | 187 |
| H-atom treatment | H atoms treated by a mixture of independent and constrained refinement |
| $\Delta\rho_{max}$, $\Delta\rho_{min}$ (e Å$^{-3}$) | 0.29, -0.24 |

**Crystal structure of (*Z*)-3**

Single crystals were grown from a solution in $CHCl_3$.

All reflection intensities were measured at 110(2) K using a SuperNova diffractometer (equipped with Atlas detector) with Mo *Kα* radiation ($\lambda$ = 0.71073 Å) under the program CrysAlisPro (Version CrysAlisPro 1.171.39.29c, Rigaku OD, 2017). The same program was used to refine the cell dimensions and for data reduction. The structure was solved with the program SHELXS-2018/3 (Sheldrick, 2018) and was refined on $F^2$ with SHELXL-2018/3 (Sheldrick, 2018). Numerical absorption correction based on gaussian integration over a multifaceted crystal model was applied using CrysAlisPro. The temperature of the data collection was controlled using the system Cryojet (manufactured by Oxford Instruments). The H atoms were placed at calculated positions (unless otherwise specified) using the instructions AFIX 43 with isotropic displacement parameters having values 1.2 $U_{eq}$ of the attached C atoms. The H atom attached to N7 was found from difference Fourier map, and its coordinates were refined pseudofreely using the DFIX instruction in order to keep the N–H bond distance within an acceptable range.

The structure is ordered.

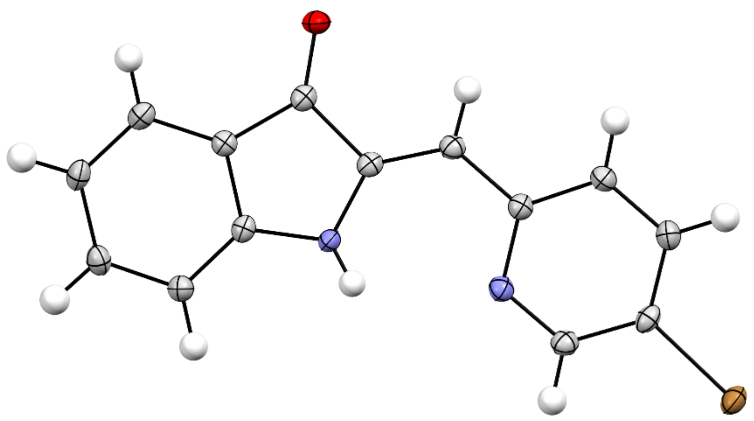

**Figure S17.** Displacement ellipsoid plot (50% probability level) of (*Z*)-**3** at 110(2) K.

**Table S3.** Crystallographic data for the structure of (*Z*)-**3**.

| | (*Z*)-**3** |
|---|---|
| Crystal data | |
| Chemical formula | $C_{14}H_9BrN_2O$ |
| $M_r$ | 301.14 |
| Crystal system, space group | Monoclinic, $P2_1/n$ |
| Temperature (K) | 110 |
| *a*, *b*, *c* (Å) | 6.4263 (2), 10.0678 (3), 18.4821 (6) |
| β (°) | 99.928 (3) |
| *V* (Å$^3$) | 1177.86 (6) |
| *Z* | 4 |
| Radiation type | Mo *K*α |
| μ (mm$^{-1}$) | 3.48 |
| Crystal size (mm) | 0.35 × 0.25 × 0.24 |
| | |
| Data collection | |
| Diffractometer | SuperNova, Dual, Cu at zero, Atlas |
| Absorption correction | Gaussian *CrysAlis PRO* 1.171.41.93a (Rigaku Oxford Diffraction, 2020) Numerical absorption correction based on gaussian integration over a multifaceted crystal model Empirical absorption correction using spherical harmonics, implemented in SCALE3 ABSPACK scaling algorithm. |
| $T_{min}$, $T_{max}$ | 0.474, 0.908 |
| No. of measured, independent and observed [$I > 2\sigma(I)$] reflections | 21070, 2710, 2506 |
| $R_{int}$ | 0.031 |

| | |
|---|---|
| $(\sin \theta/\lambda)_{max}$ (Å$^{-1}$) | 0.650 |
| | |
| Refinement | |
| $R[F^2 > 2\sigma(F^2)]$, $wR(F^2)$, $S$ | 0.021, 0.054, 1.05 |
| No. of reflections | 2710 |
| No. of parameters | 166 |
| No. of restraints | 1 |
| H-atom treatment | H atoms treated by a mixture of independent and constrained refinement |
| $\Delta\rho_{max}$, $\Delta\rho_{min}$ (e Å$^{-3}$) | 0.38, -0.31 |

## Crystal structure of (*Z*)-5

Single crystals were grown by slow diffusion of pentane into a solution in THF.

All reflection intensities were measured at 110(2) K using a SuperNova diffractometer (equipped with Atlas detector) with Mo *K*α radiation (λ = 0.71073 Å) under the program CrysAlisPro (Version CrysAlisPro 1.171.42.49, Rigaku OD, 2022). The same program was used to refine the cell dimensions and for data reduction. The structure was solved with the program SHELXS-2018/3 (Sheldrick, 2018) and was refined on $F^2$ with SHELXL-2018/3 (Sheldrick, 2018). Numerical absorption correction based on gaussian integration over a multifaceted crystal model was performed using CrysAlisPro. The temperature of the data collection was controlled using the system Cryojet (manufactured by Oxford Instruments). The H atoms were placed at calculated positions (unless otherwise specified) using the instructions AFIX 43 or AFIX 137 with isotropic displacement parameters having values 1.2 or 1.5 $U_{eq}$ of the attached C atoms. The H atom attached to N7 was found from difference Fourier map, and its coordinates and isotropic temperature factor were refined freely.

The structure is ordered. The crystal that was mounted on the diffractometer was found to be a composite of two crystal components related by a 4.38° rotation along [-0.57 -0.48 -0.67]. The BASF scale factor refines to 0.07(3).

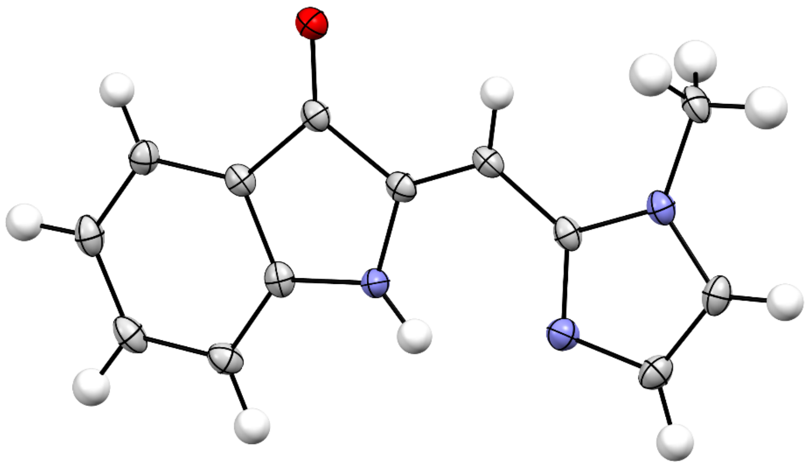

**Figure S18.** Displacement ellipsoid plot (50% probability level) of (*Z*)-**5** at 110(2) K.

**Table S4.** Crystallographic data for the structure of (*Z*)-**5**.

| | (*Z*)-**5** |
|---|---|
| Crystal data | |
| Chemical formula | $C_{13}H_{11}N_3O$ |
| $M_r$ | 225.25 |
| Crystal system, space group | Monoclinic, $P2_1/c$ |
| Temperature (K) | 110 |
| *a*, *b*, *c* (Å) | 12.6840 (8), 7.9316 (3), 12.0346 (8) |
| β (°) | 116.692 (8) |
| *V* (Å$^3$) | 1081.71 (13) |
| *Z* | 4 |
| Radiation type | Mo *K*α |
| μ (mm$^{-1}$) | 0.09 |
| Crystal size (mm) | 0.18 × 0.14 × 0.10 |
| | |
| Data collection | |
| Diffractometer | SuperNova, Dual, Cu at zero, Atlas |
| Absorption correction | Gaussian<br>*CrysAlis PRO* 1.171.42.49 (Rigaku Oxford Diffraction, 2022) Numerical absorption correction based on gaussian integration over a multifaceted crystal model Empirical absorption correction using spherical harmonics, implemented in SCALE3 ABSPACK scaling algorithm. |
| $T_{min}$, $T_{max}$ | 0.900, 1.000 |
| No. of measured, independent and observed [$I > 2\sigma(I)$] reflections | 12731, 3586, 2227 |
| $R_{int}$ | 0.042 |
| $(\sin \theta/\lambda)_{max}$ (Å$^{-1}$) | 0.650 |
| | |
| Refinement | |
| $R[F^2 > 2\sigma(F^2)]$, $wR(F^2)$, $S$ | 0.039, 0.088, 0.83 |
| No. of reflections | 3586 |
| No. of parameters | 160 |
| H-atom treatment | H atoms treated by a mixture of independent and constrained refinement |
| $\Delta\rho_{max}$, $\Delta\rho_{min}$ (e Å$^{-3}$) | 0.26, -0.21 |

**Crystal structure of (*Z*)-6**

Single crystals were grown from a solution in $Et_2O$.

All reflection intensities were measured at 110(2) K using a SuperNova diffractometer (equipped with Atlas detector) with Mo *K*α radiation (λ = 0.71073 Å) under the program CrysAlisPro (Version CrysAlisPro 1.171.42.49, Rigaku OD, 2022). The same program was used to refine the cell dimensions and for data reduction. The structure was solved with the program SHELXS-2018/3 (Sheldrick, 2018) and was refined on $F^2$ with SHELXL-2018/3 (Sheldrick, 2018). Numerical absorption correction based on gaussian integration over a multifaceted crystal model was performed using CrysAlisPro. The temperature of the data collection was controlled using the system Cryojet (manufactured by Oxford Instruments). The H atoms were placed at calculated positions (unless otherwise specified) using the instructions AFIX 43 or AFIX 137 with isotropic displacement parameters having values 1.2 or 1.5 $U_{eq}$ of the attached C atoms. The H atom attached to N7 was found from difference Fourier map, and its coordinates and isotropic temperature factor were refined freely.

The structure is ordered.

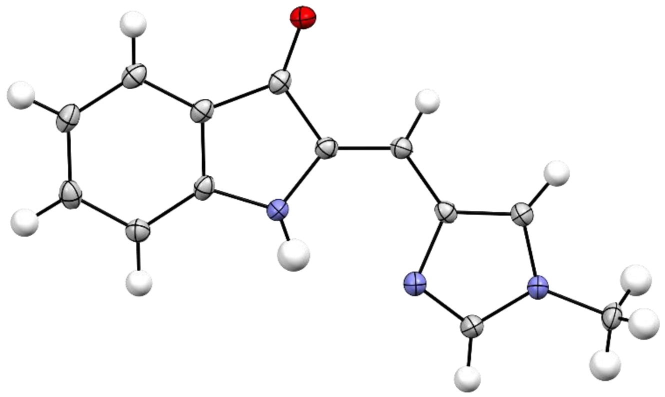

**Figure S19.** Displacement ellipsoid plot (50% probability level) of (*Z*)-**6** at 110(2) K.

**Table S5.** Crystallographic data for the structure of (*Z*)-**6**.

| | (*Z*)-**6** |
|---|---|
| Crystal data | |
| Chemical formula | $C_{13}H_{11}N_3O$ |
| $M_r$ | 225.25 |
| Crystal system, space group | Monoclinic, *I*2/*a* |
| Temperature (K) | 110 |
| *a*, *b*, *c* (Å) | 20.5498 (14), 3.8316 (3), 27.2403 (18) |
| β (°) | 103.238 (7) |
| *V* (Å$^3$) | 2087.9 (3) |
| *Z* | 8 |
| Radiation type | Mo *K*α |
| μ (mm$^{-1}$) | 0.10 |
| Crystal size (mm) | 0.14 × 0.07 × 0.06 |
| | |
| Data collection | |
| Diffractometer | SuperNova, Dual, Cu at zero, Atlas |
| Absorption correction | Gaussian<br>*CrysAlis PRO* 1.171.42.49 (Rigaku Oxford Diffraction, 2022) Numerical absorption correction based on gaussian integration over a multifaceted crystal model Empirical absorption correction using spherical harmonics, implemented in SCALE3 ABSPACK scaling algorithm. |
| $T_{min}$, $T_{max}$ | 0.855, 1.000 |
| No. of measured, independent and observed [$I > 2\sigma(I)$] reflections | 11387, 2046, 1632 |
| $R_{int}$ | 0.056 |
| $(\sin \theta/\lambda)_{max}$ (Å$^{-1}$) | 0.617 |
| | |
| Refinement | |
| $R[F^2 > 2\sigma(F^2)]$, $wR(F^2)$, $S$ | 0.045, 0.113, 1.08 |
| No. of reflections | 2046 |
| No. of parameters | 159 |
| H-atom treatment | H atoms treated by a mixture of independent and constrained refinement |
| $\Delta\rho_{max}$, $\Delta\rho_{min}$ (e Å$^{-3}$) | 0.19, -0.25 |

**Table S6.** Central double bond length, dihedral angle, and shortest nitrogen-nitrogen distance in the solid-state structures of (*Z*)-**1**-**3** and (*Z*)-**5**-**6** including estimated standard deviations.

| Hemi-indigo | N–C=C–$C_{Ar}$ dihedral [°] | C=C bond length [Å] | N(H)···N distance [Å] |
|---|---|---|---|
| (*Z*)-**1** | -1.7(2)[a] | 1.3453(16)[a] | 2.8212(14)[a] |
| (*Z*)-**2** | -2.3(2) | 1.3545(18) | 2.7211(15) |
| (*Z*)-**3** | 1.7(3) | 1.346(2) | 2.8568(19) |
| (*Z*)-**5** | 0.4(3) | 1.350(2) | 2.8008(19) |
| (*Z*)-**6** | -0.6(3) | 1.344(3) | 2.830(2) |

[a] For comparison: in the DFT-optimized structure of (*Z*)-**1** this dihedral angle is 0.0°, and the C=C bond length and N-N distance are 1.353 Å and 2.81 Å, respectively.

# 4. $^{1}$H NMR titrations with TFA

## Addition of TFA to (*Z*)-1

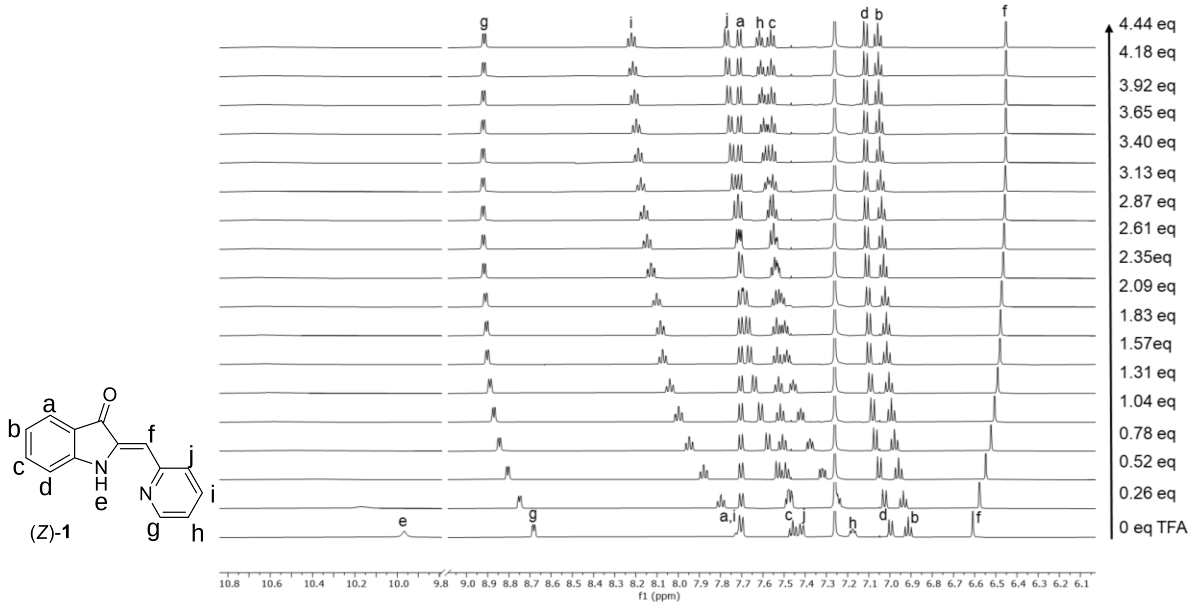


**Figure S20.** $^{1}$H NMR (500 MHz, $CDCl_3$) spectral changes (from bottom to top) starting with (*Z*)-**1** (5.6 mM in $CDCl_3$) upon stepwise addition of a solution of TFA (0.18 M in $CDCl_3$).

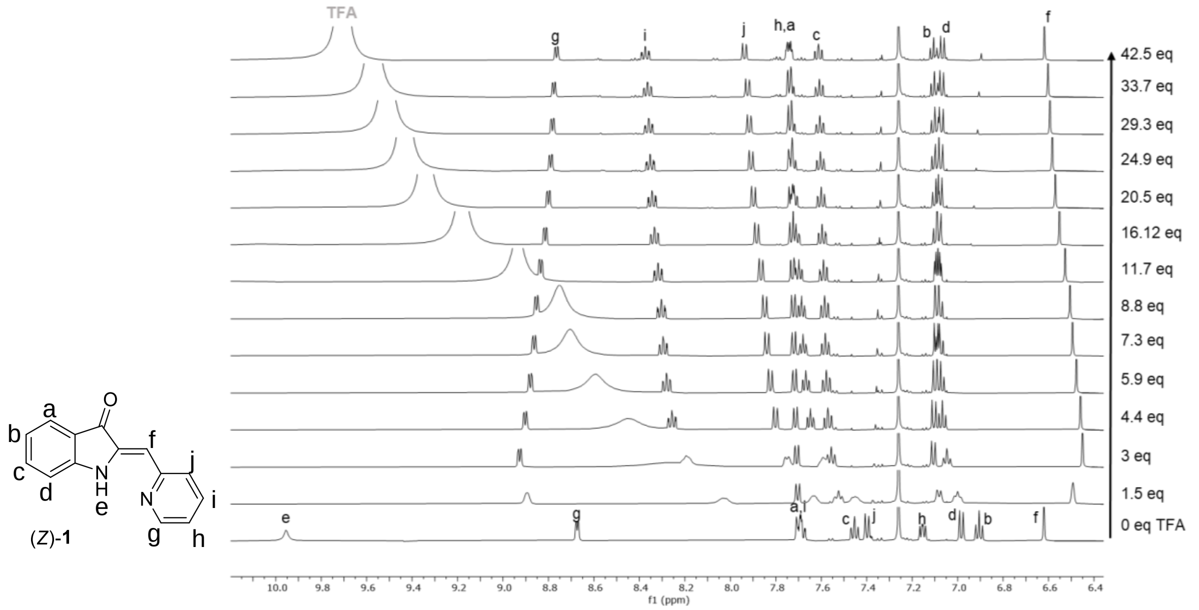


**Figure S21.** $^{1}$H NMR (500 MHz, $CDCl_3$) spectral changes (from bottom to top) starting with (*Z*)-**1** (6.7 mM in $CDCl_3$) upon stepwise addition of TFA (1.18 M in $CDCl_3$). Note the inversion in chemical shift changes for signals **g**, **f**, and **d** indicating a secondary process.

## Addition of TFA to pyridine

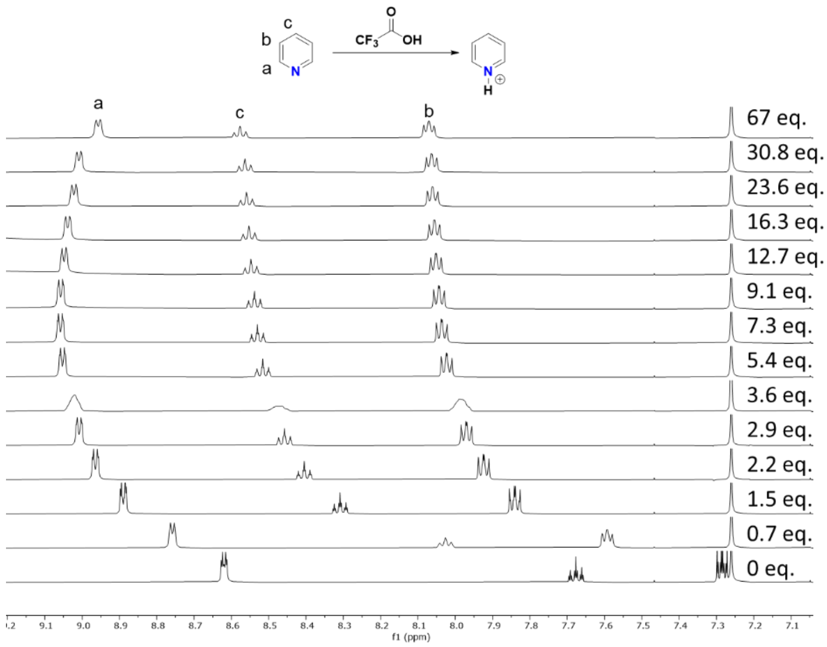


**Figure S22.** $^1$H NMR (500 MHz) spectral changes (from bottom to top) of pyridine (6 mM, $CDCl_3$) upon stepwise addition of a solution of 1.3 M TFA (from 0 equiv. to 30.8 equiv.) and neat TFA (final addition from 30.8 equiv. to 67 equiv.) displaying a similar inversion in chemical shift (H**a**) changes as in Figure **S21**.

## 5. $^{1}$H NMR studies of acid/base controlled isomerization

### Acid-induced *Z*→*E* isomerization

The desired amount of TFA was added to solutions of hemi-indigos **1-6** (5.7-5.8 mM in $CDCl_3$, 0.60 mL), and directly after addition a $^{1}$H NMR spectrum was recorded (defined as $t = 0$). Partial isomerization was sometimes observed already at this point due to the exothermic protonation event and the inevitable mixing time prior to data acquisition. The generation of (*E*)·$\mathbf{H^+}$ was monitored over time by $^{1}$H NMR spectroscopy and in case thermal equilibration took longer than 12 h, the experiment was performed in a flame-sealed NMR tube to avoid TFA evaporation (as noted in the figure captions). Flame-sealing of the NMR tubes was performed after measuring the first spectrum. $CDCl_3$ was filtered over basic $Al_2O_3$ and dried over 4 Å molecular sieves. $^{1}$H NMR signal assignments are based on 2D COSY and/or NOESY spectra.

### Subsequent *E*→Z back isomerization

Thermally equilibrated samples of hemi-indigos **1-6** (in the presence of 32 equiv. TFA) were freshly prepared as described above, and subsequently neutralized with $Et_3N$ (1.5 equiv. with respect to TFA). Isomerization was followed over time by $^{1}$H NMR spectroscopy. $CDCl_3$ was filtered over basic $Al_2O_3$ and dried over 4 Å molecular sieves. All experiments were performed with the same batch of solvent, TFA and $Et_3N$. Samples were sonicated before each measurement to ensure homogeneity.

**Note:** We observed that residual TFA may catalyze the backwards isomerization process, and that the amount of $Et_3N$ used to deprotonate the switch thus affects the observed rate.

## Acid-induced *Z*→*E* isomerization of 1

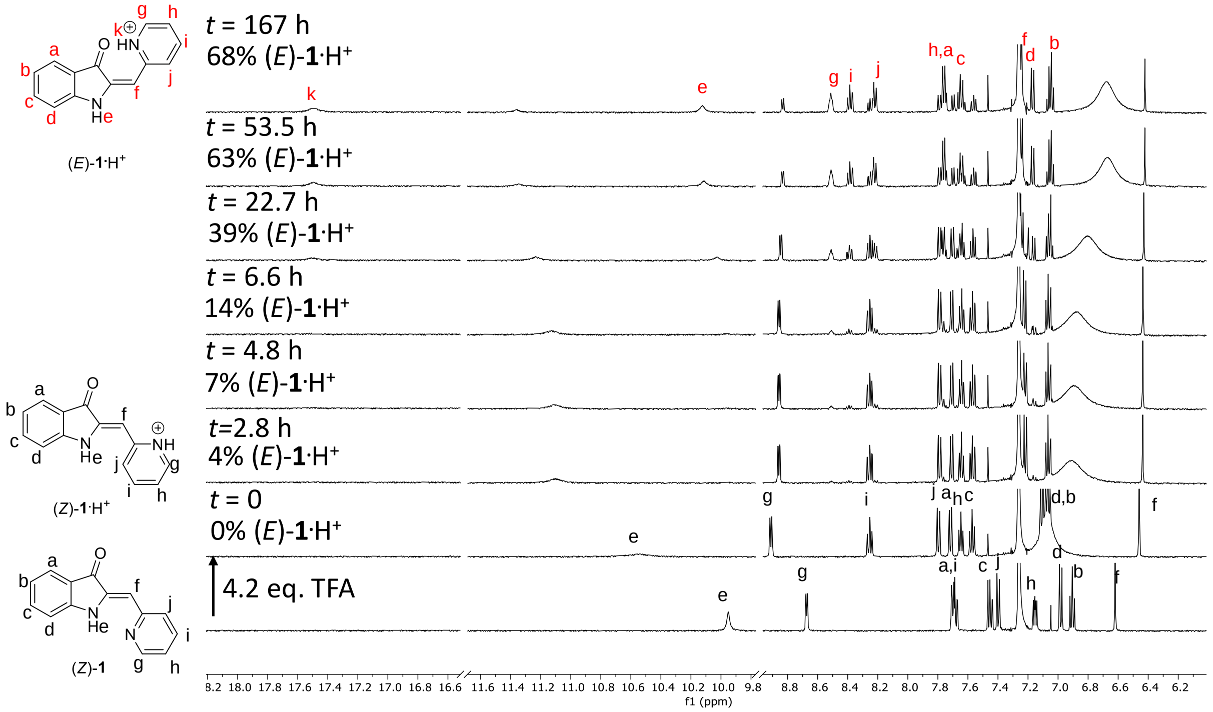


**Figure S23.** $^{1}$H NMR spectral changes (500 MHz, $CDCl_3$) of (*Z*)-**1** (5.7 mM) followed over time (from bottom to top) in a flame-sealed NMR tube after addition of 4.2 equiv. of neat TFA. The isomer ratio was determined by integration of the aromatic H**g** signals.

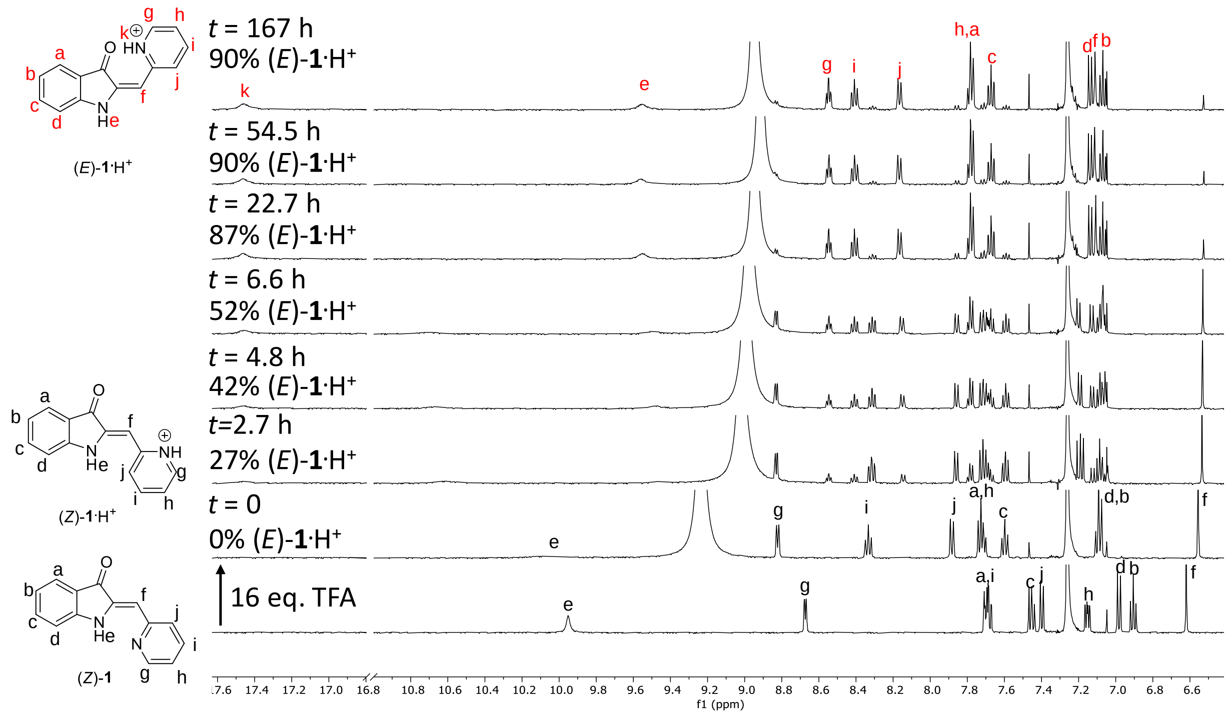


**Figure S24**. $^{1}$H NMR spectral changes (500 MHz, $CDCl_3$) of (*Z*)-**1** (5.7 mM) followed over time (from bottom to top) in a flame-sealed NMR tube after addition of 16 equiv. of neat TFA. The isomer ratio was determined by integration of the aromatic H**i** signals.

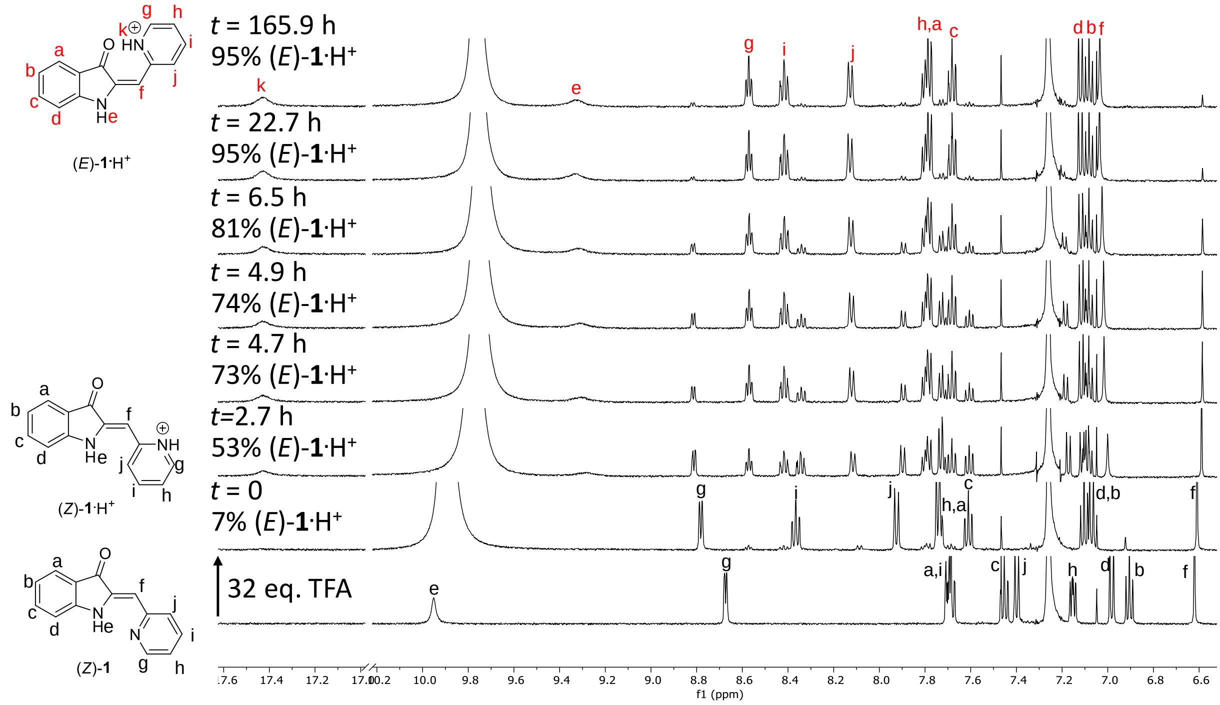


**Figure S25.** $^1$H NMR spectral changes (500 MHz, $CDCl_3$) of (Z)-**1** (5.7 mM) followed over time (from bottom to top) in a flame-sealed NMR tube after addition of 32 equiv. of neat TFA. The isomer ratio was determined by integration of the aromatic H**g** signals.

## Subsequent *E*→*Z* back isomerization of 1

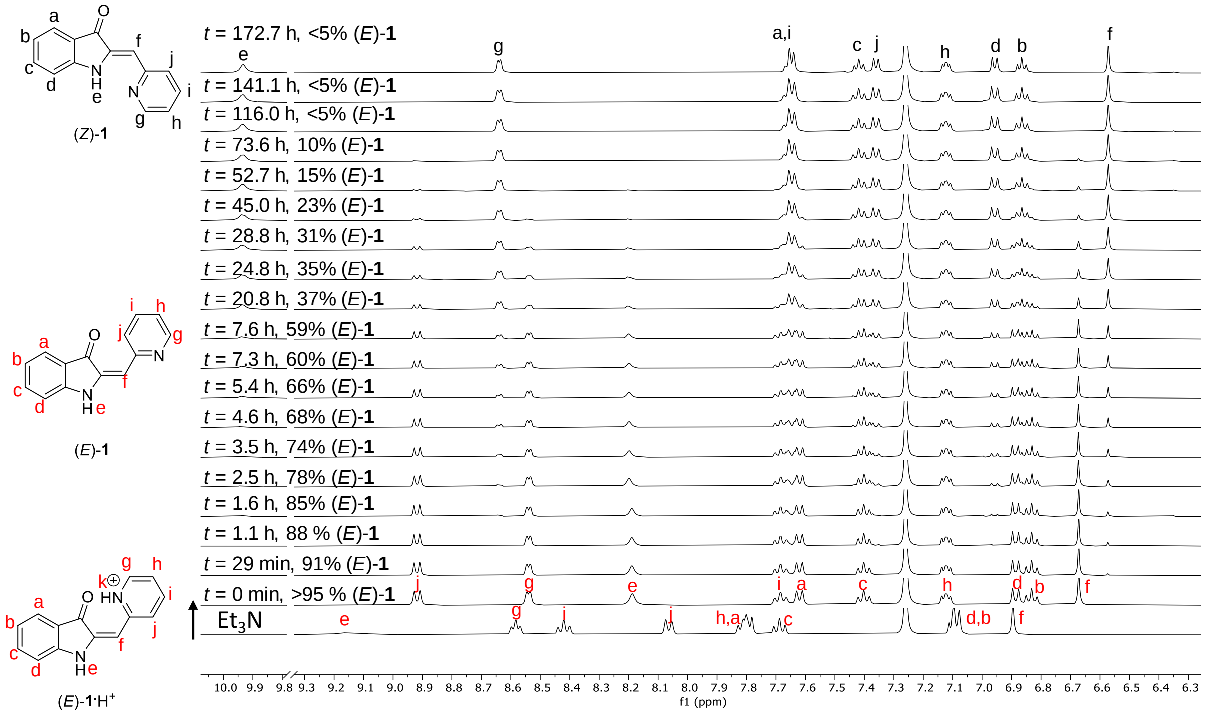


**Figure S26.** $^1$H NMR spectral changes (400 and 500 MHz, $CDCl_3$) of (*E*)-**1·H**$^+$ (5.7 mM) equilibrated in presence of 32 equiv. of TFA followed over time (from bottom to top) after addition of 48 equiv. of $Et_3N$. The isomer ratio was determined by integration of the alkene H**f** signals. Minor precipitation was observed during overnight measurements (between t = 7.6 h and 20.8 h) and sonication was therefore applied to the sample to ensure homogeneity. Note that upon addition of base most pyridyl proton signals shift upfield, indicative of deprotonation. The signal of pyridyl proton H**j**, however, was found to shift downfield. Upon deprotonation, the pyridyl nitrogen will likely rotate away from the carbonyl group, placing this pyridyl proton in close proximity to the anisotropic carbonyl deshielding cone.

## Acid-induced *Z*→*E* isomerization of 2

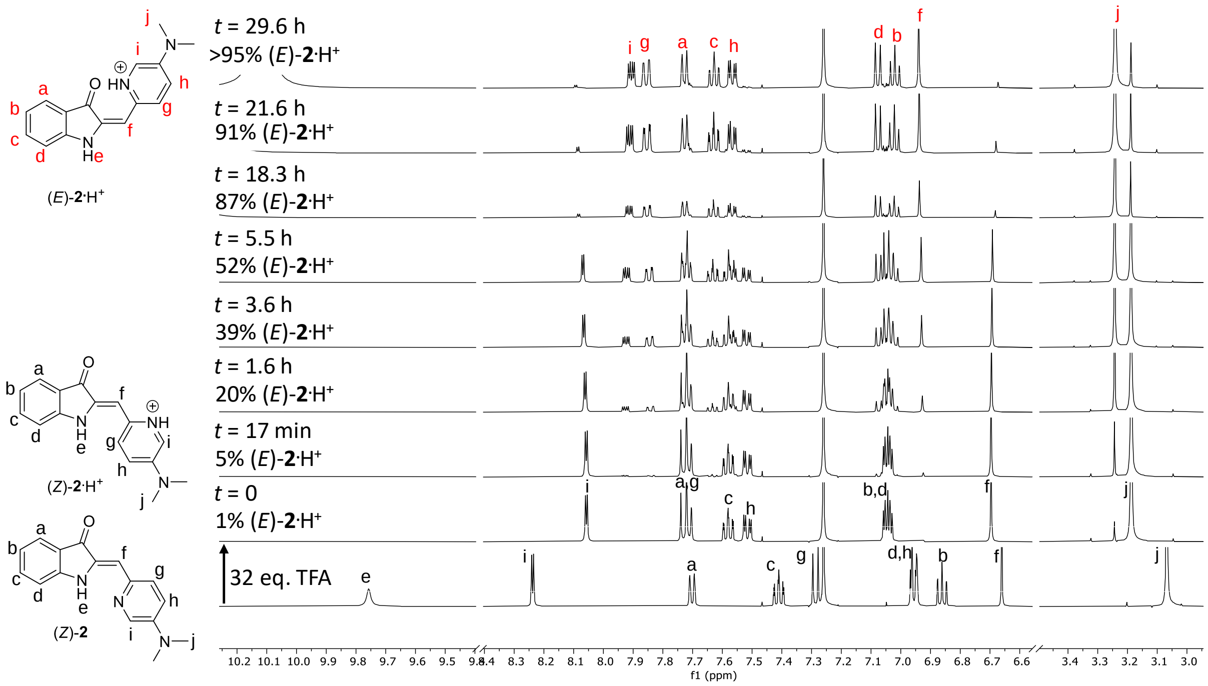


**Figure S27.** $^1$H NMR spectral changes (500 MHz, $CDCl_3$) of (*Z*)-**2** (5.8 mM) followed over time (from bottom to top) after addition of 32 equiv. TFA. The isomer ratio was determined by integration of the alkene H**f** signals.

## Subsequent *E*→*Z* back isomerization of 2

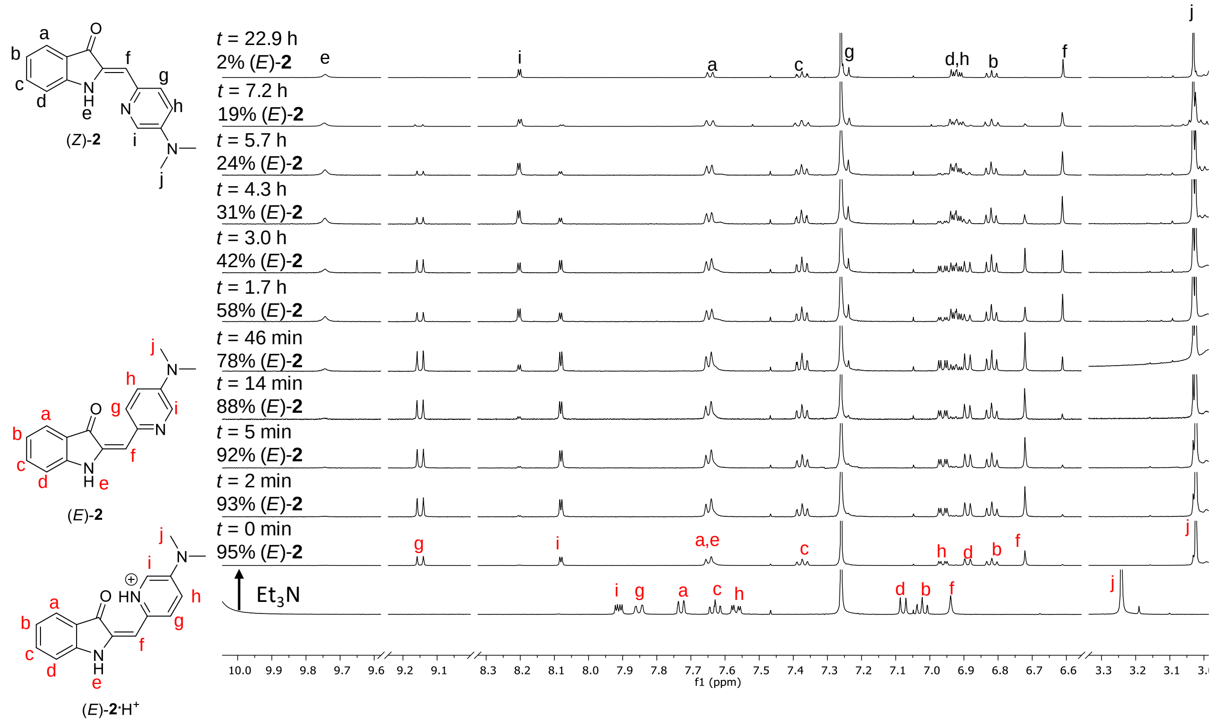


**Figure S28.** $^{1}$H NMR spectral changes (400 MHz, $CDCl_3$) of (*E*)-**2·H**$^{+}$ (5.7 mM) equilibrated in presence of 32 equiv. TFA followed over time (from bottom to top) after addition of 48 equiv. $Et_3N$. The isomer ratio was determined by integration of the alkene H**f** signals.

## Acid-induced *Z*→*E* isomerization of 3

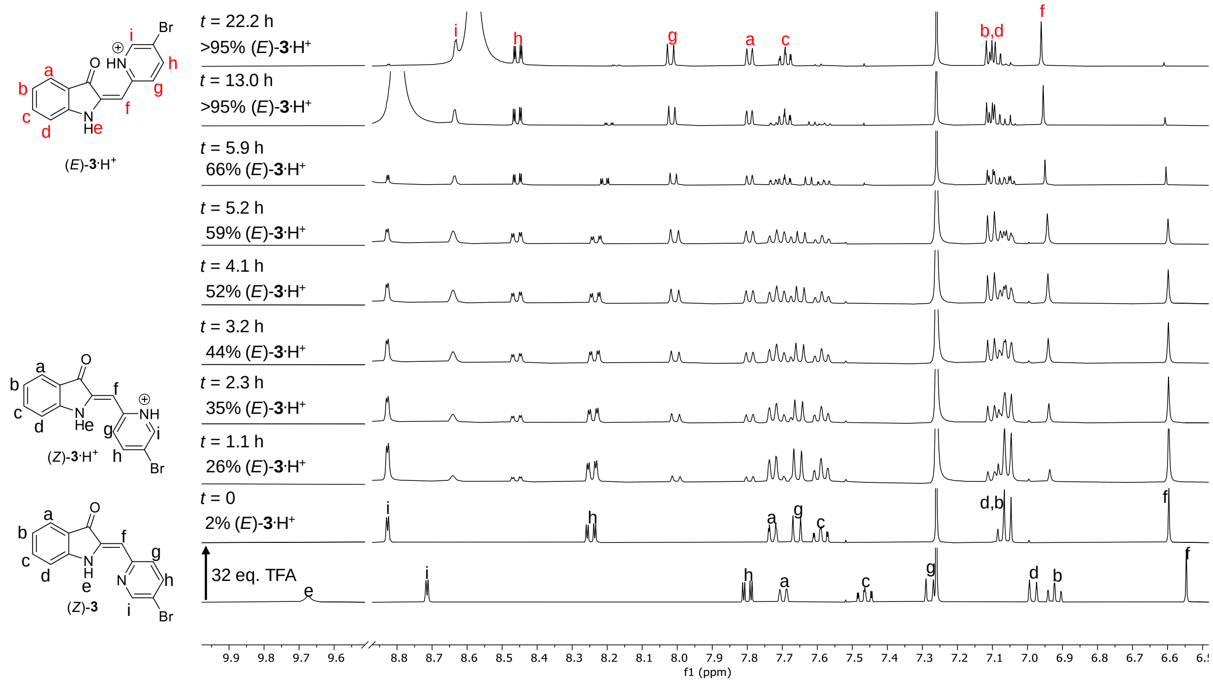


**Figure S29.** $^{1}$H NMR spectral changes (500 MHz, $CDCl_3$) followed over time (from bottom to top) of (*Z*)-**3** (5.7 mM) after addition of 32 equiv. TFA. The isomer ratio was determined by integration of the alkene H**f** signals.

## Subsequent $E \rightarrow Z$ back isomerization of 3

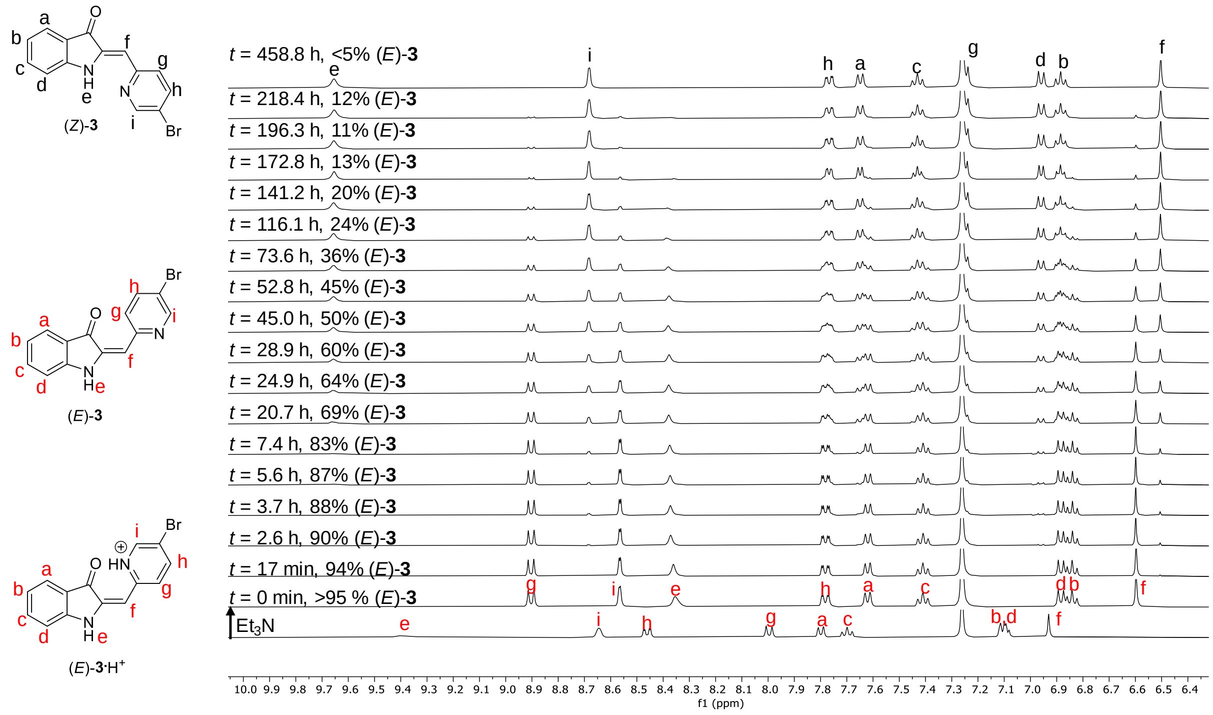


**Figure S30.** $^{1}$H NMR spectral changes (500 and 400 MHz, $CDCl_3$) of (*E*)-**3·H**$^{+}$ (5.7 mM) equilibrated in presence of 32 equiv. TFA followed over time (from bottom to top) after addition of 48 equiv. $Et_3N$. The isomer ratio was determined by integration of the alkene H**f** signals.

## Acid-induced *Z*→*E* isomerization of 4

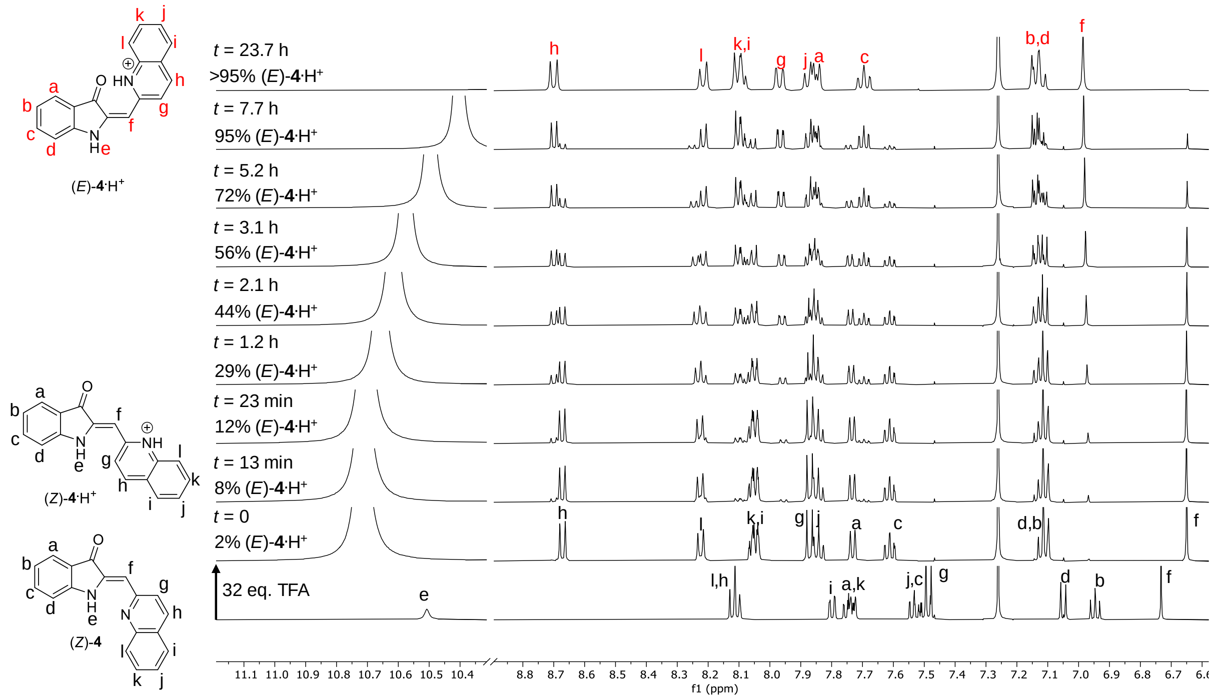


**Figure S31.** $^{1}$H NMR spectral changes (500 MHz, $CDCl_3$) of (*Z*)-**4** (5.7 mM) followed over time (from bottom to top) after addition of 32 equiv. TFA. The isomer ratio was determined by integration of the alkene H**f** signals.

## Subsequent $E \rightarrow Z$ isomerization of 4

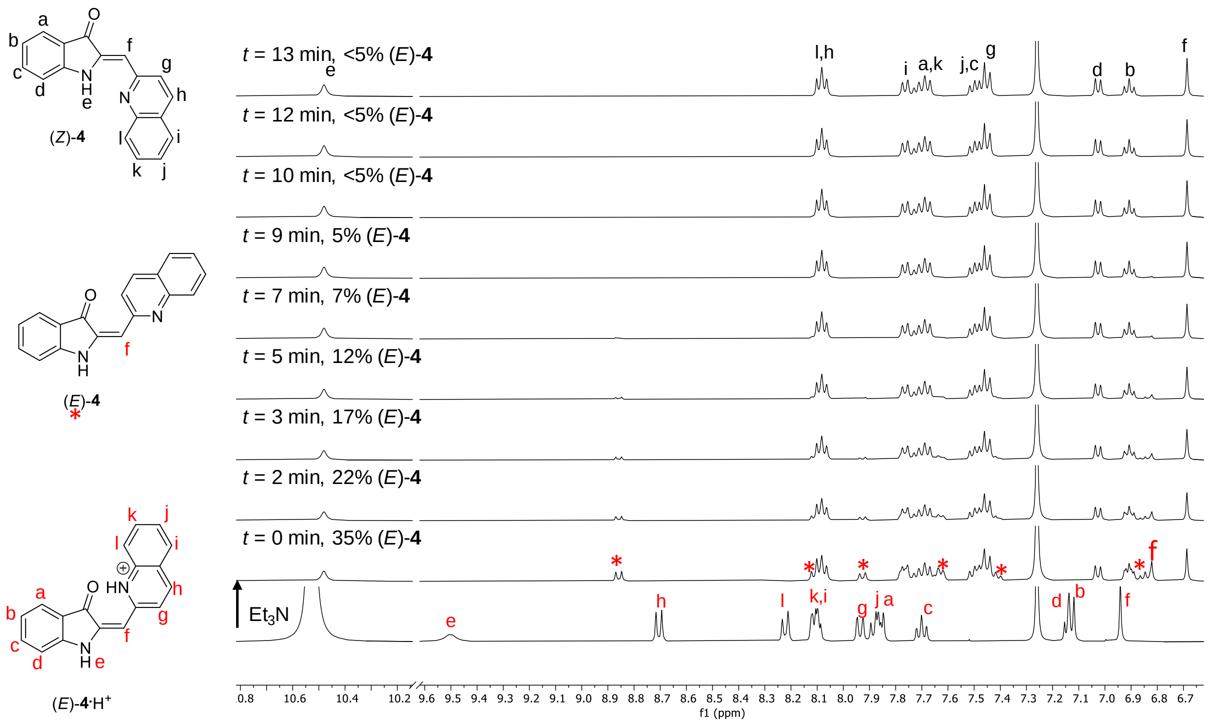


**Figure S32.** $^1$H NMR spectral changes (500 and 400 MHz, $CDCl_3$) of (*E*)-**4·H**$^+$ (5.7 mM) equilibrated in presence of 32 equiv. TFA followed over time (from bottom to top) after addition of 48 equiv. $Et_3N$. The isomer ratio was determined by integration of the alkene H**f** signals. Full characterization of (*E*)-**4** was omitted due to its short thermal half-life.

## Acid-induced *Z*→*E* isomerization of 5

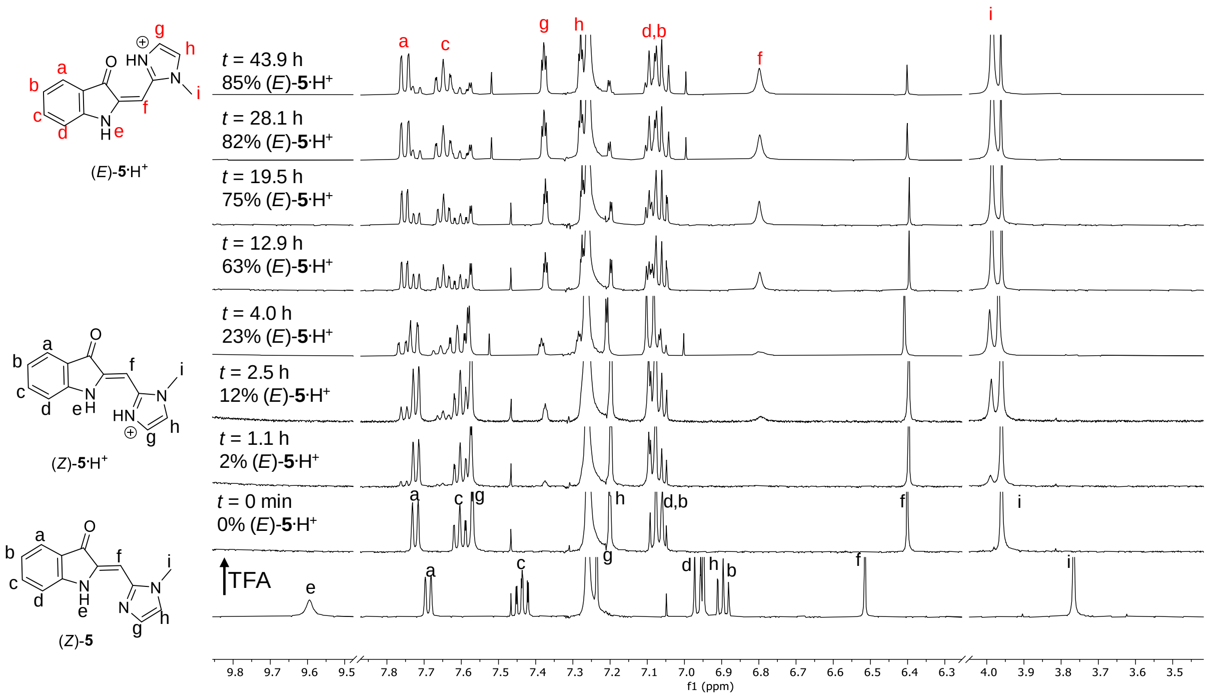

**Figure S33.** $^{1}$H NMR spectral changes (500 and 400 MHz, $CDCl_3$) of (*Z*)-**5** (5.7 mM) followed over time (from bottom to top) in a flame-sealed NMR tube after addition of 32 equiv. TFA. The isomer ratio was determined by integration of the alkene H**f** signals.

## Subsequent *E*→*Z* back isomerization of 5

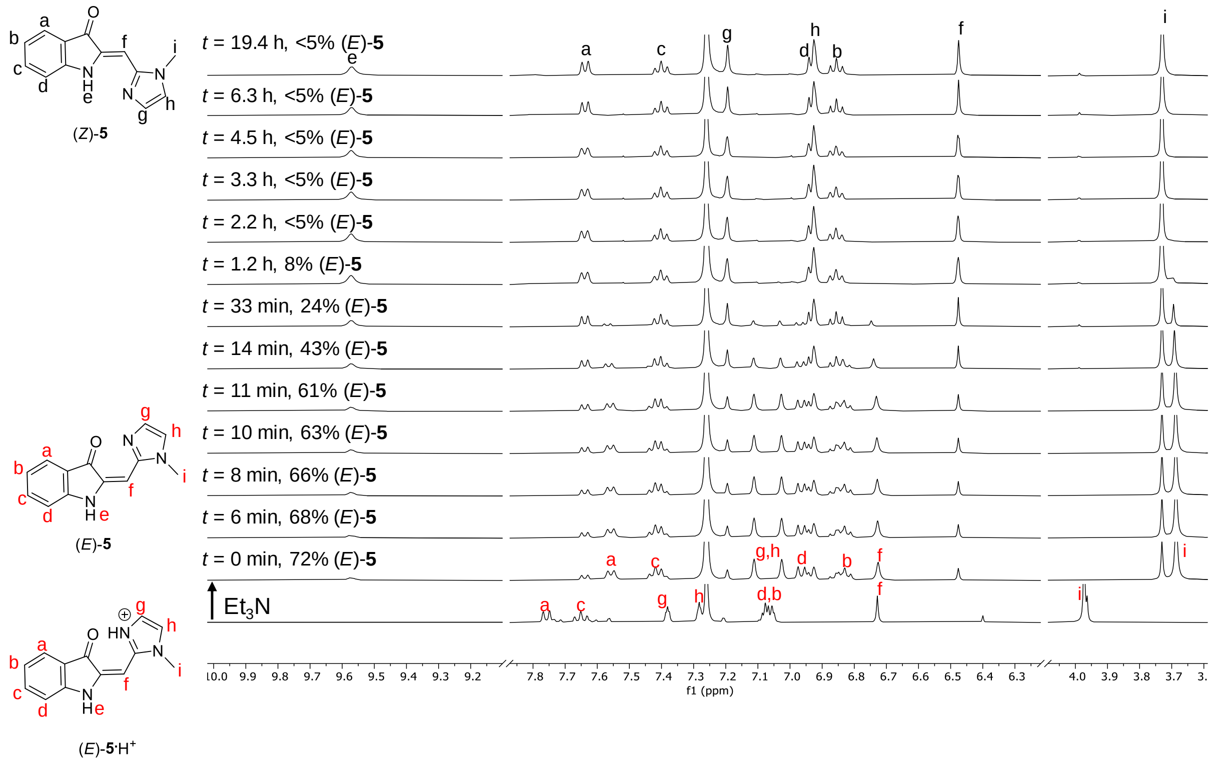


**Figure S34.** $^{1}$H NMR spectral changes (400 MHz, $CDCl_3$) of (*E*)-**5·H**$^+$ (5.7 mM) equilibrated in presence of 32 equiv. TFA followed over time (from bottom to top) after addition of 48 equiv. $Et_3N$. The isomer ratio was determined by integration of the alkene H**f** signals.

## Acid-induced *Z*→*E* isomerization of 6

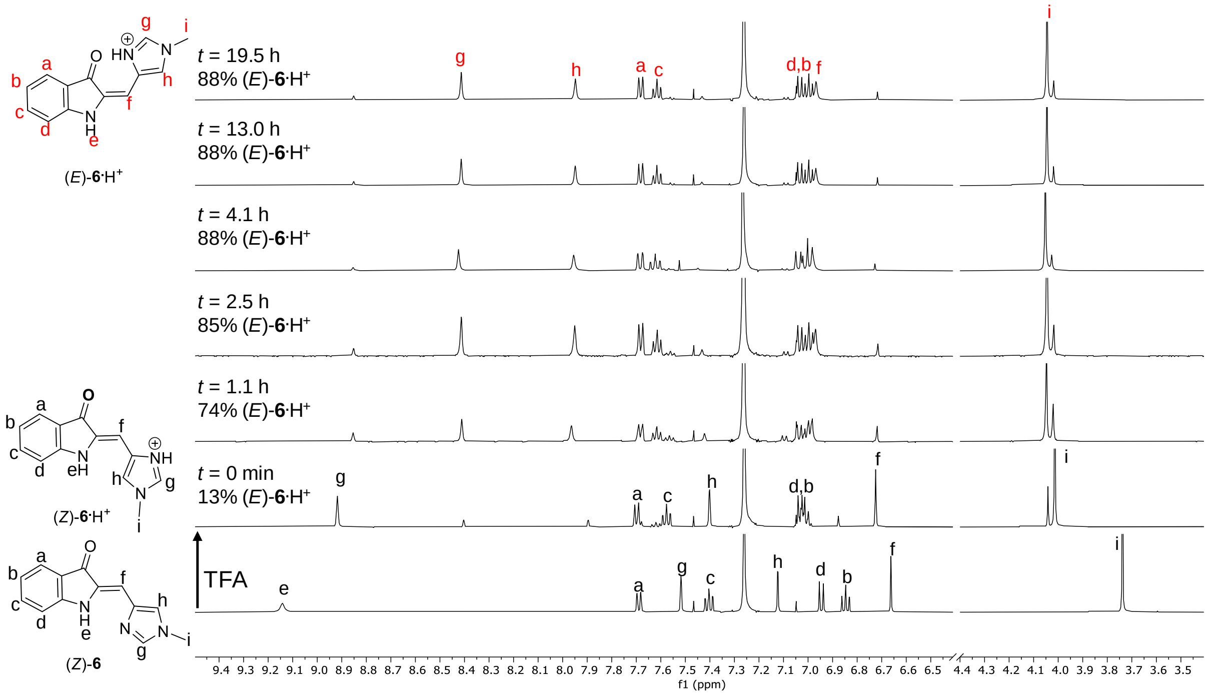


**Figure S35.** $^{1}$H NMR spectral changes (500 MHz, $CDCl_3$) of (*Z*)-**6** (5.7 mM) followed over time (from bottom to top) in a flame-sealed NMR tube after addition of 32 equiv. TFA. The isomer ratio was determined by integration of the H**i** signals.

## Subsequent *E*→*Z* back isomerization of 6

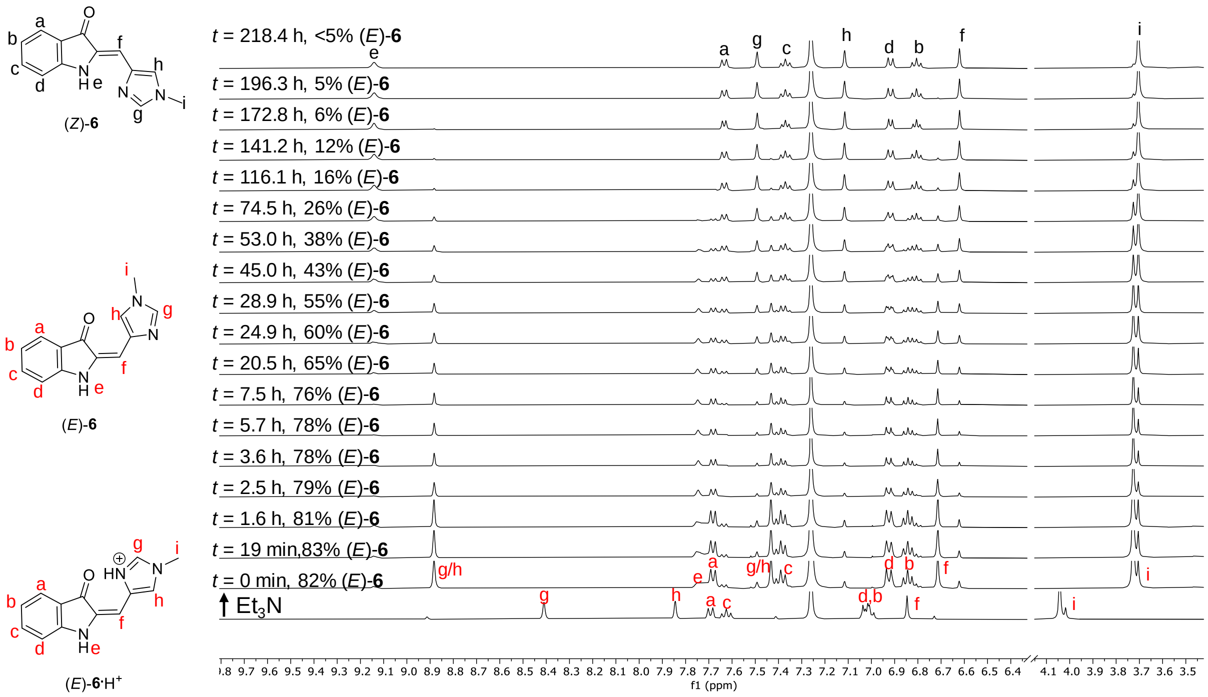


**Figure S36.** $^{1}$H NMR spectral changes (400 and 500 MHz, $CDCl_3$) of (*E*)-**6·H**$^{+}$ (5.7 mM) equilibrated in presence of 32 equiv. TFA followed over time (from bottom to top) after addition of 48 equiv. $Et_3N$. The isomer ratio was determined by integration of the alkene H**f** signals.

**NOE-DIFF spectrum of (*E*)-1·H⁺**

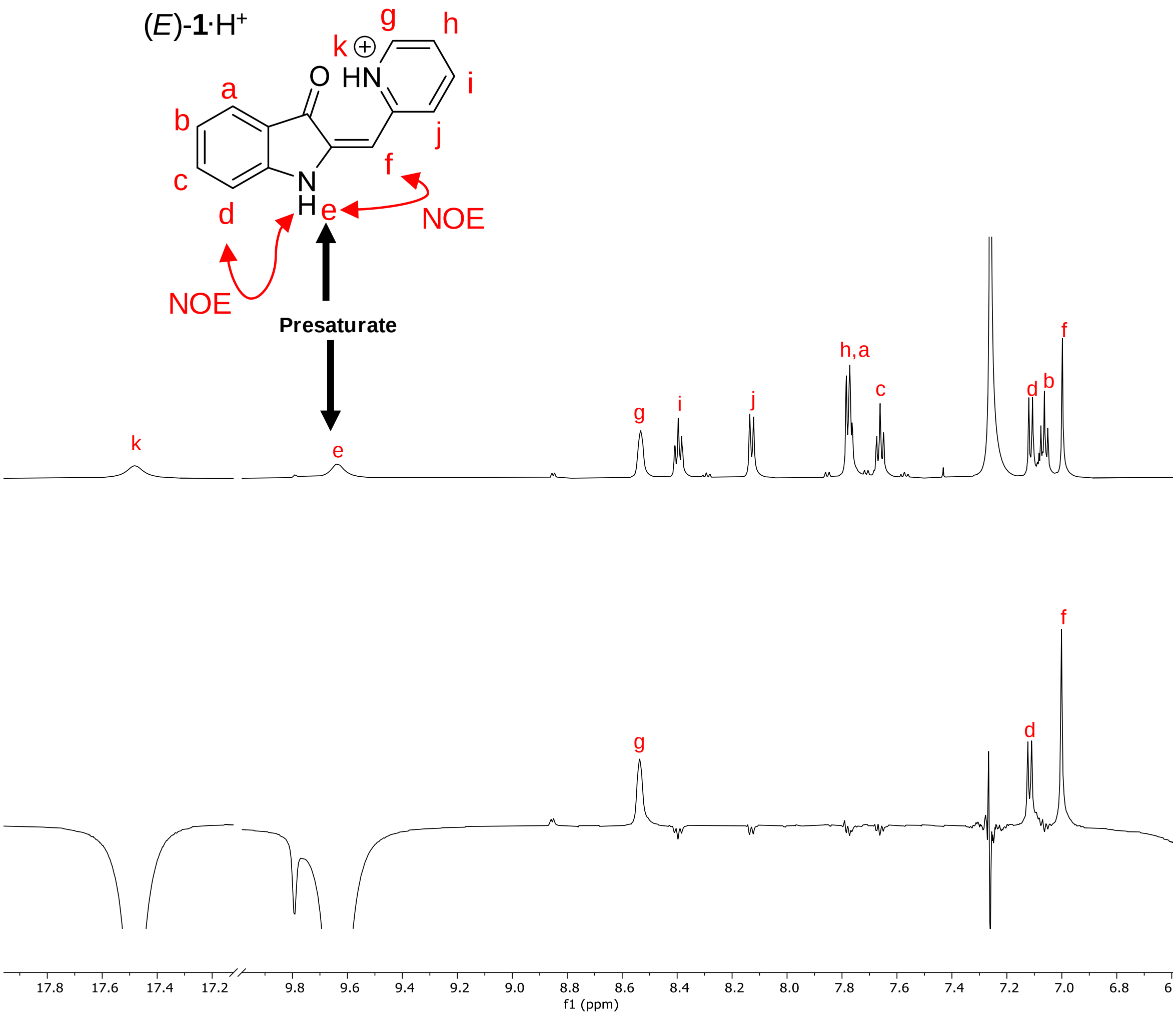


**Figure S37.** $^1$H-NMR (600 MHz, $CDCl_3$) (top) and NOE-DIFF NMR (600 MHz, $CDCl_3$) spectrum (bottom) of (*E*)-**1**·**H**$^+$ in presence of excess TFA: presaturation pulse at 9.64 ppm; control pulse at 13.06 ppm; pulse length: 3 seconds at 48 dB (1.5849E-5 W). Measured at 298 K. Please note that due to chemical exchange between the acidic protons **k**, **e**, water and TFA presaturation of signal H**e** inevitably leads to presaturation of H**k**. Hence, besides the NOE interaction of H**e** with H**d** and H**f** the NOE interaction between H**k** and H**g** is also observed. Nevertheless, the through-space interaction between H**e** and H**f** supports the (*E*)-configuration of the protonated and thermodynamically more stable form of compound **1**.

## NOESY NMR spectrum of (*E*)-1·H⁺

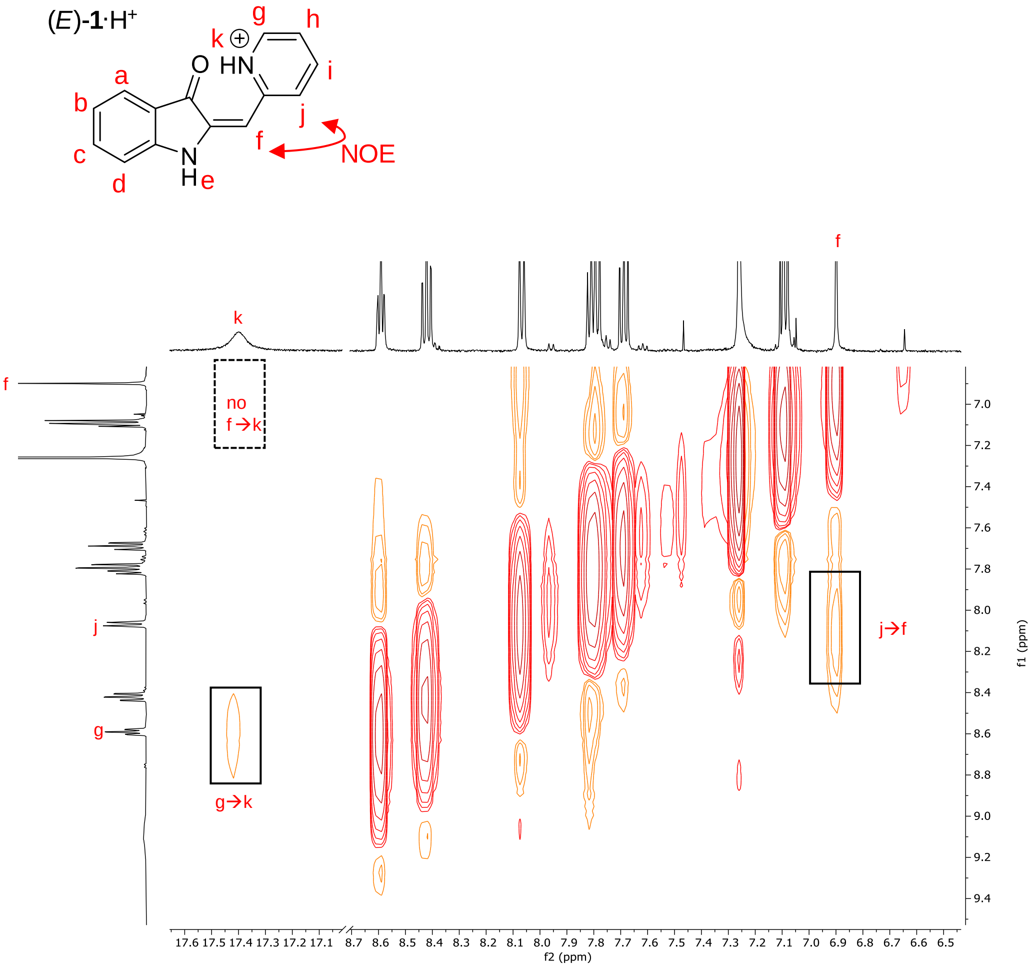


**Figure S38.** 2D NOESY NMR spectrum (500 MHz, $CDCl_3$) of (*E*)-**1**·**H**$^+$ in presence of excess TFA. The through space interaction between H**j** and H**f** (and the lack of such an interaction between H**k** and H**f**) indicates that the rotational conformer of **1** with the pyridinium proton H**k** oriented towards the carbonyl oxygen is the predominant species in solution.

## 6. Fitting of $^1$H NMR isomerization data to first-order decay kinetics

The first order decay in concentration of the protonated (*Z*)-isomer of the hemi-indigo dyes was fitted to the equation $A = A_0e^{-kt} + y0$ ($A = A_0e^{-t/t1} + y0$) using Origin software to obtain the rate constant (*k*).

### Acid-induced *Z*→*E* isomerization of 1

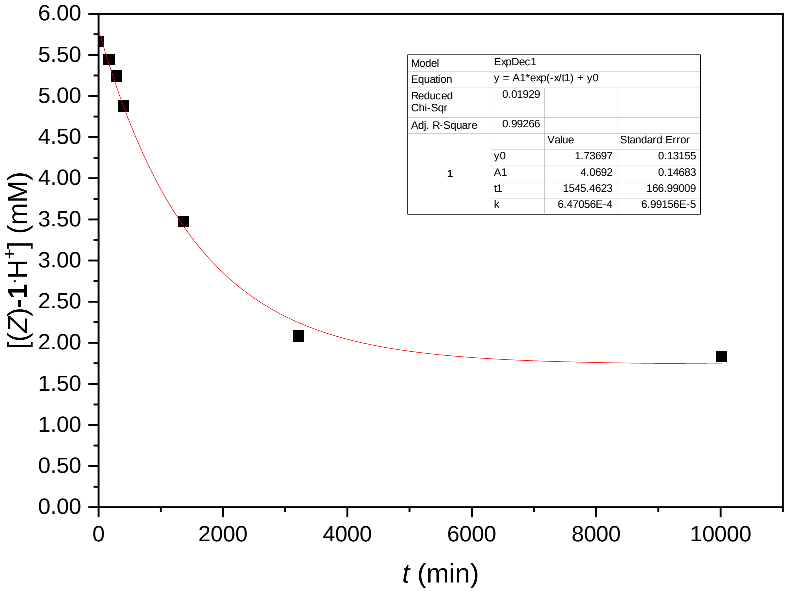


**Figure S39.** Change in concentration of (*Z*)-**1·H**$^+$ in presence of 4.2 equiv. TFA, monitored over time by $^1$H NMR spectroscopy (500 MHz). See Figure **S23** for the corresponding $^1$H NMR spectra and experimental conditions.

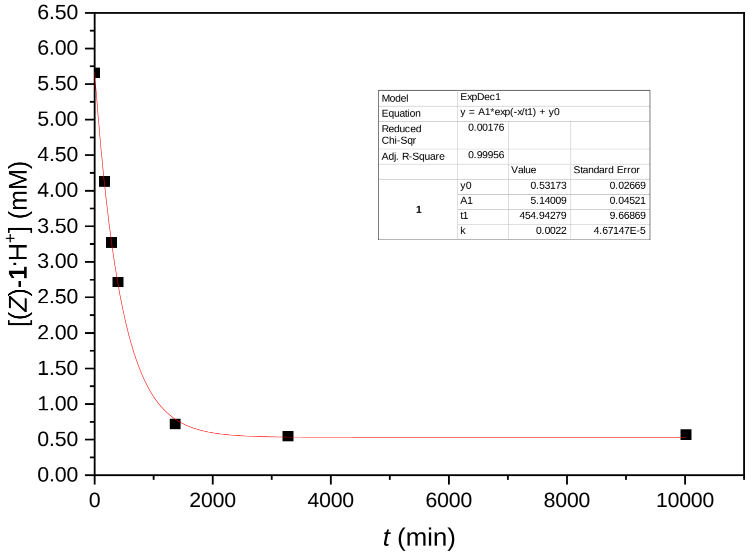


**Figure S40.** Change in concentration of ($Z$)-**1·H**$^+$ in presence of 16 equiv. TFA, monitored over time by $^1$H NMR spectroscopy (500 MHz). See Figure **S24** for the corresponding $^1$H NMR spectra and experimental conditions.

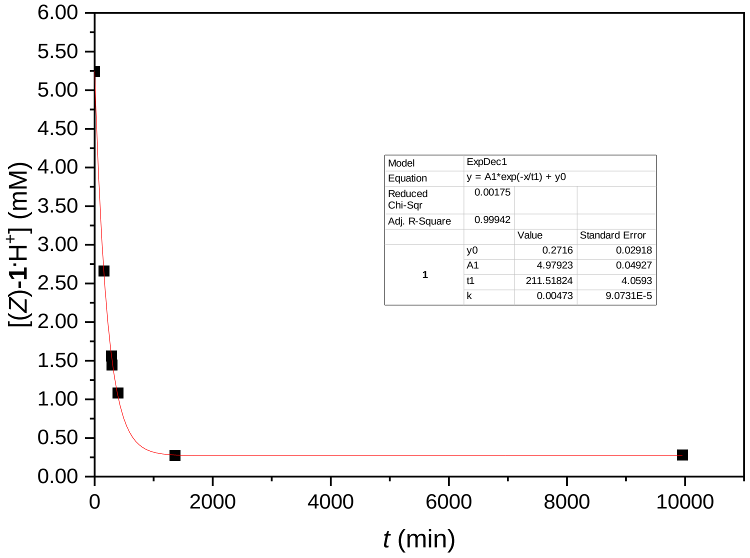


**Figure S41.** Change in concentration of ($Z$)-**1·H**$^+$ in presence of 32 equiv. TFA, monitored over time by $^1$H NMR spectroscopy (500 MHz). See Figure **S25** for the corresponding $^1$H NMR spectra and experimental conditions.

## Subsequent *E*→*Z* back isomerization of 1

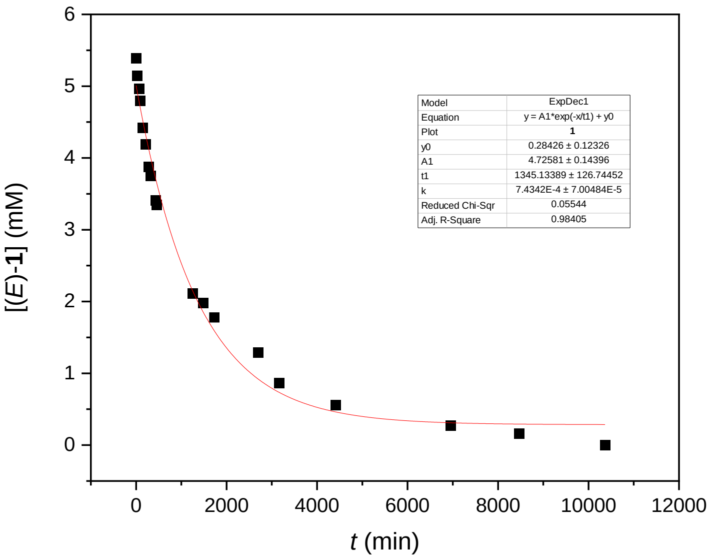


**Figure S42.** Change in concentration of (*E*)-**1** (5.7 mM) monitored by $^{1}$H NMR spectroscopy (400 and 500 MHz). See Figure **S26** for the corresponding $^{1}$H NMR spectra and experimental conditions.

## Acid-induced *Z*→*E* isomerization of 2

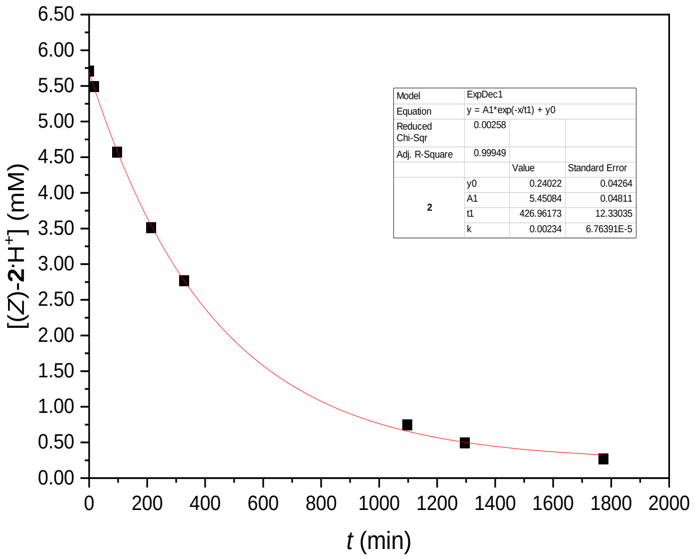


**Figure S43.** Change in concentration of **(**Z)**-2·H**$^{+}$ monitored over time by $^{1}$H NMR spectroscopy (500 MHz). See Figure **S27** for corresponding $^{1}$H NMR spectra and experimental conditions.

## Subsequent *E*→*Z* back isomerization of 2

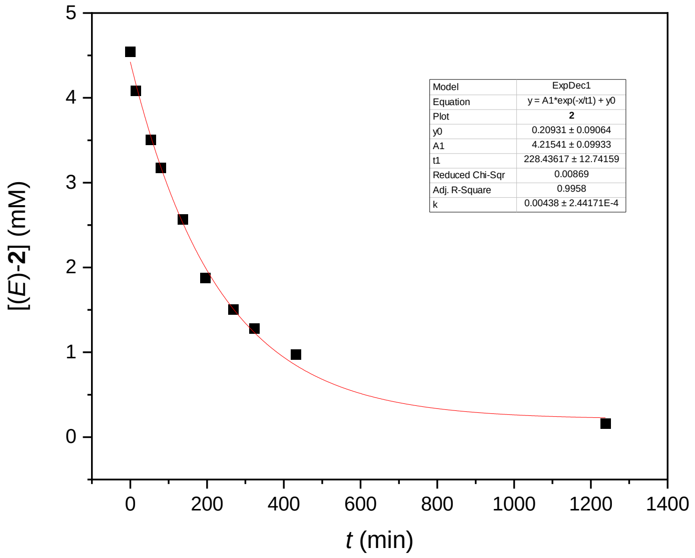


**Figure S44.** Change in concentration of (*E*)-**2** monitored by $^{1}$H NMR spectroscopy (500 and 400 MHz, $CDCl_3$). See Figure **S28** for the corresponding $^{1}$H NMR spectra and experimental conditions.

## Acid-induced *Z*→*E* isomerization of 3

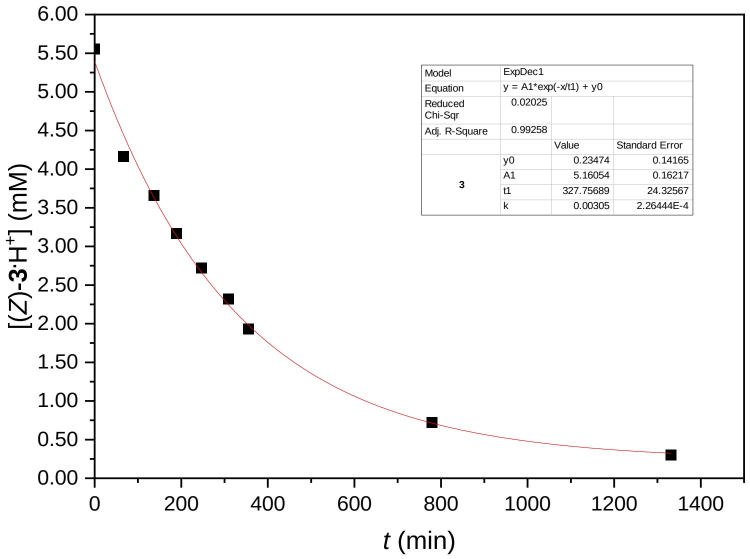


**Figure S45.** Change in concentration of **(***Z***)-3·H**$^{+}$ monitored over time by $^{1}$H NMR spectroscopy (500 MHz). See Figure **S29** for corresponding $^{1}$H NMR spectra and experimental conditions.

## Subsequent *E*→*Z* back isomerization of 3

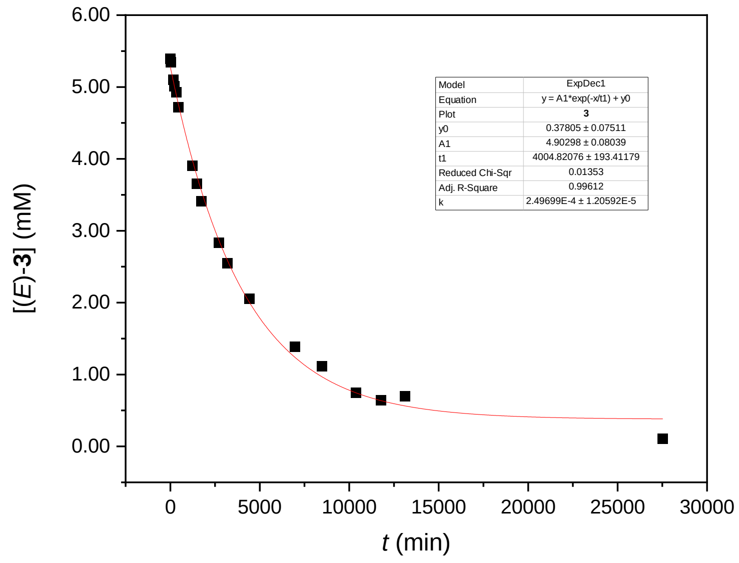


**Figure S46.** Concentration of (*E*)-**3** (5.7 mM) monitored by $^{1}$H NMR spectroscopy (400 and 500 MHz). See Figure **S30** for the corresponding $^{1}$H NMR spectra and experimental conditions.

## Acid-induced *Z*→*E* isomerization of 4

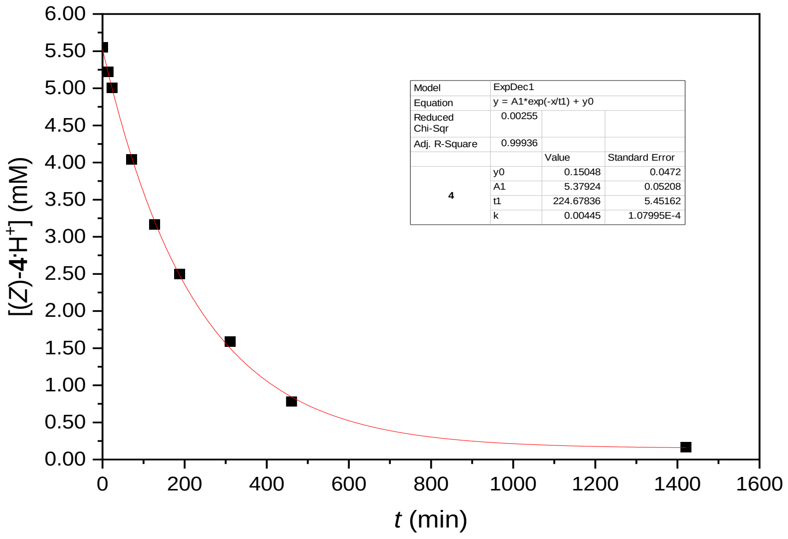


**Figure S47.** Change in concentration of (*Z*)-**4·H**$^+$ monitored over time by $^1$H NMR spectroscopy (500 MHz). See Figure **S31** for corresponding $^1$H NMR spectra and experimental conditions.

## Subsequent *E*→*Z* back isomerization of 4

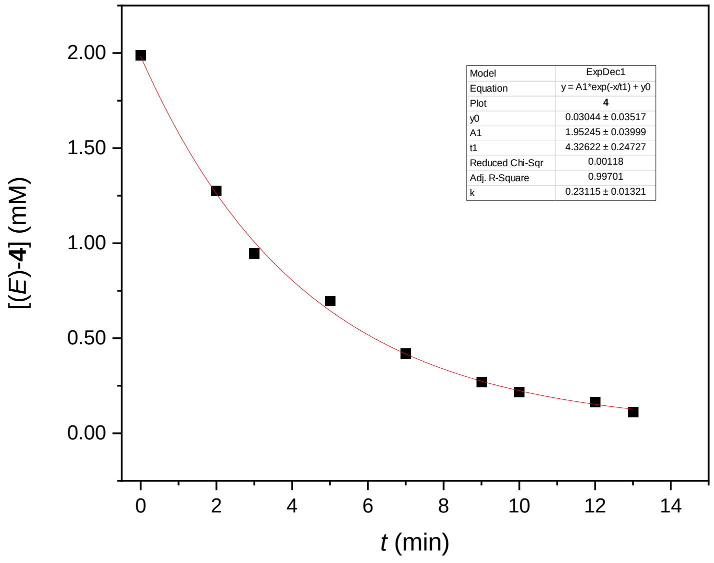


**Figure S48.** Concentration of (*E*)-**4** monitored by $^1$H NMR spectroscopy (400 MHz). See Figure **S32** for the corresponding $^1$H NMR spectra and experimental conditions.

## Acid-induced *Z*→*E* isomerization of 5

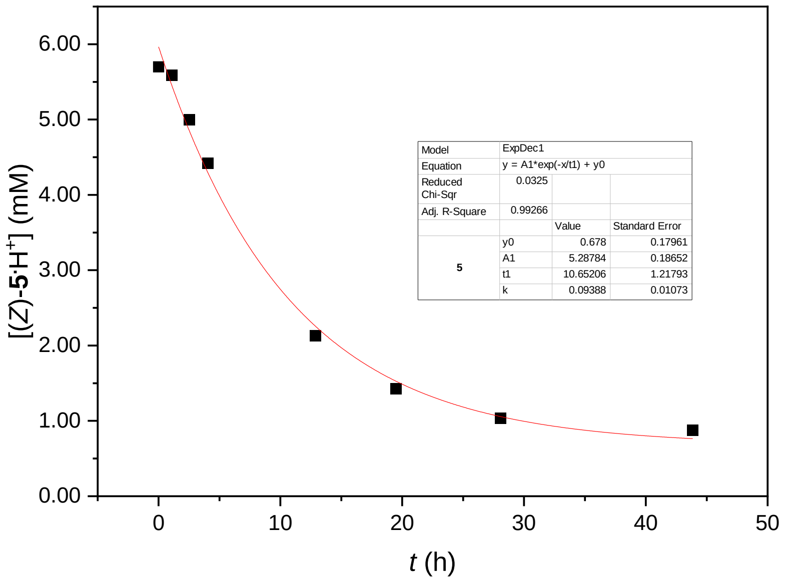


**Figure S49.** Change in concentration of **(***Z***)-5·H**$^+$ monitored over time by $^1$H NMR spectroscopy (500 and 400 MHz). See Figure **S33** for the corresponding $^1$H NMR spectra and experimental conditions.

## Subsequent *E*→*Z* back isomerization of 5

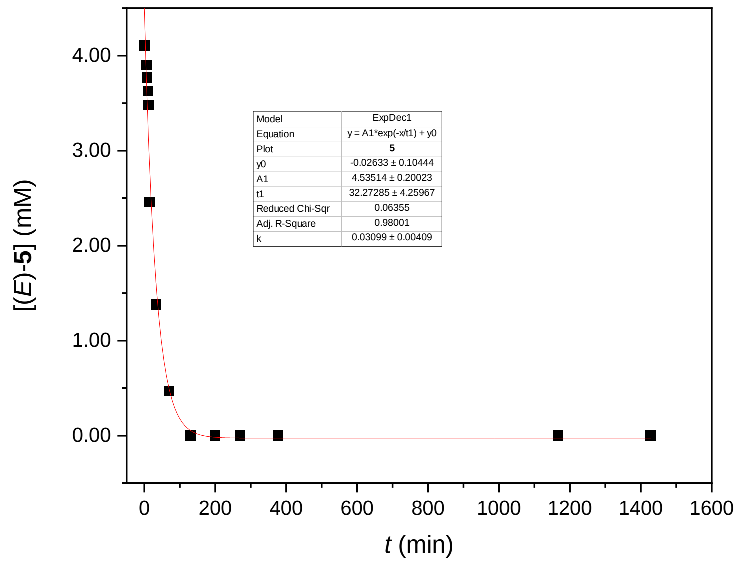


**Figure S50.** Change in concentration of (*E*)-**5** monitored by $^1$H NMR spectroscopy (400 MHz). See Figure **S34** for the corresponding $^1$H NMR spectra and experimental conditions.

## Acid-induced *Z*→*E* isomerization of 6

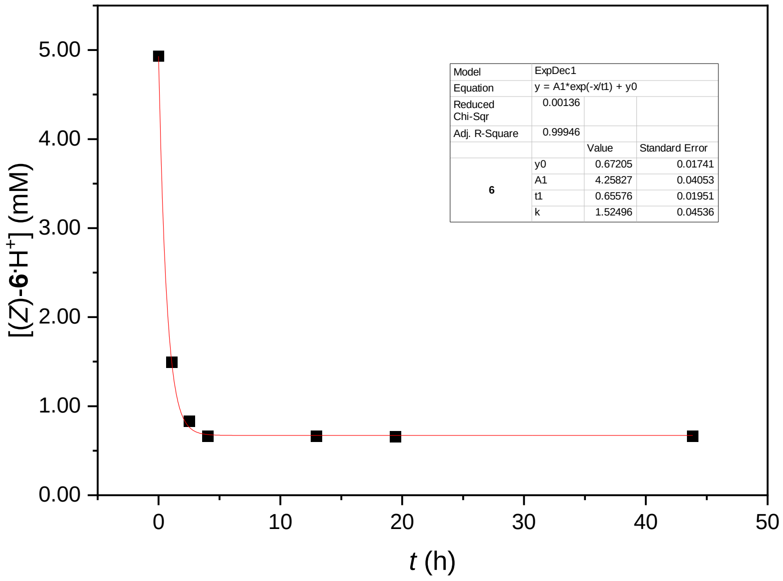


**Figure S51.** Change in concentration of **(**$Z$**)-6·H**$^+$ monitored over time by $^1$H NMR spectroscopy (500 MHz). See Figure **S35** for corresponding $^1$H NMR spectra and experimental conditions.

## Subsequent *E*→*Z* isomerization of 6

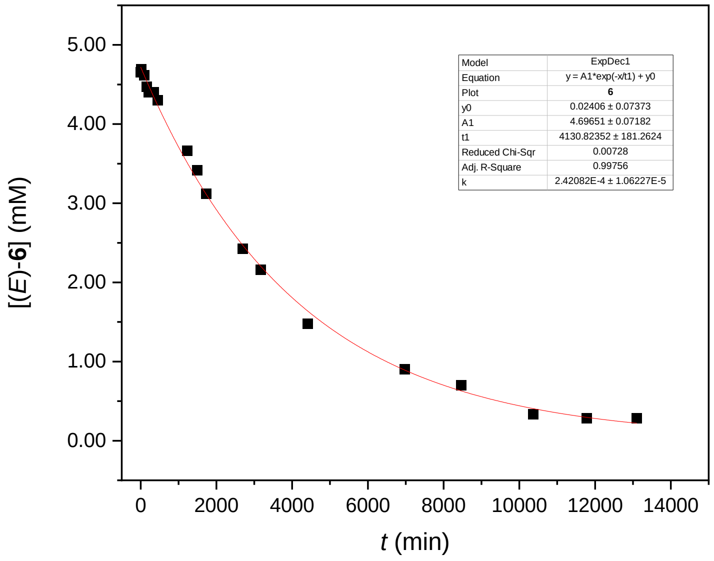


**Figure S52.** Change in concentration of ($E$)-**6** monitored by $^1$H NMR spectroscopy (500 and 400 MHz). See Figure **S36** for corresponding $^1$H NMR spectra and experimental conditions.

**Table S7.** Thermal isomerization parameters upon protonation and subsequent deprotonation.[a]

| Hemi-indigo | Ratio [$E$/$Z$·H$^+$][b] | $k_{(Z\rightarrow E)H^+}$ [min$^{-1}$][c] | $k_{(E\rightarrow Z)}$ [min$^{-1}$][c] |
|---|---|---|---|
| **1** | 95:5 | $4.73 \times 10^{-3}$ | $7.43 \times 10^{-4}$ |
| **2** | >95:5 | $2.34 \times 10^{-3}$ | $4.83 \times 10^{-3}$ |
| **3** | >95:5 | $3.05 \times 10^{-3}$ | $2.50 \times 10^{-4}$ |
| **4** | >95:5 | $4.45 \times 10^{-3}$ | $2.31 \times 10^{-1}$ |
| **5** | 85:15 | $1.56 \times 10^{-3}$ | $3.10 \times 10^{-2}$ |
| **6** | 88:12 | $2.54 \times 10^{-2}$ | $2.42 \times 10^{-4}$ |

[a] Determined by $^1$H NMR spectroscopy at 25 °C using ~6 mM solutions in $CDCl_3$ containing 32 equiv. of TFA, which were neutralized by subsequent addition of 48 equiv. of $Et_3N$. [b] Calculated by $^1$H NMR signal integration. [c] Calculated by fitting to a first order decay equation.

## 7. UV-Vis studies of acid/base controlled isomerization

### Acid-induced *Z*→*E* isomerization of 1

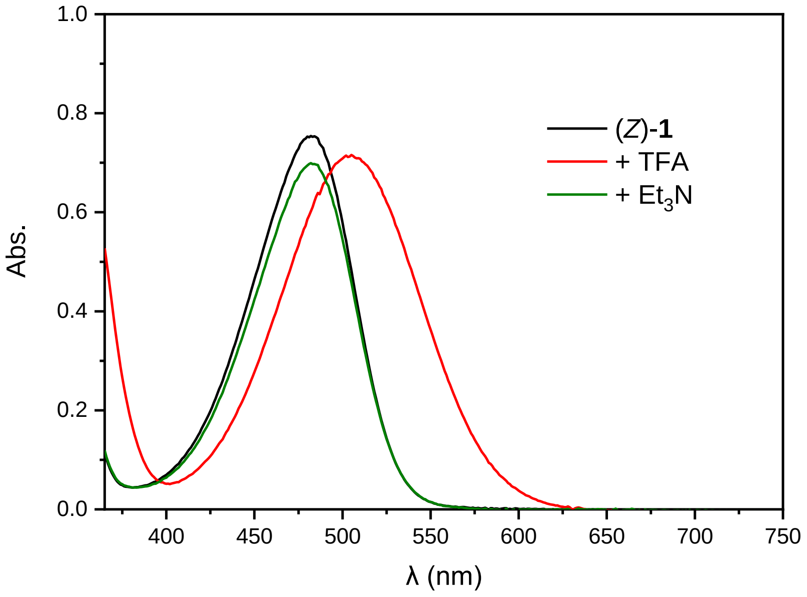


**Figure S53.** UV-Vis spectral data showing that direct addition of TFA ($4.3 \times 10^2$ equiv.) and $Et_3N$ (1.6 equiv. with respect to TFA) to (*Z*)-**1** (0.76 mM in degassed $CHCl_3$, 1 mm cuvette) gives a similar decrease in absorbance as observed for the thermally recovered species due to dilution of the sample (initial volume: 200 μL) and potential solvatochromic effects.

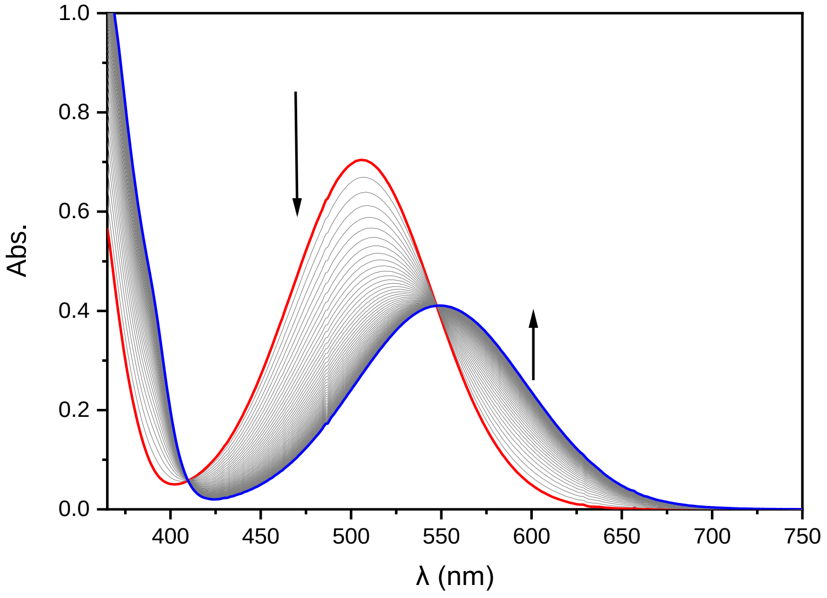


**Figure S54.** UV-Vis spectral changes of (*Z*)-**1** (0.76 mM, $CHCl_3$, 1 mm cuvette) monitored over time (15 min interval) in presence of excess TFA ($4.3 \times 10^2$ equiv.).

**Acid-induced *Z*→*E* isomerization of 2**

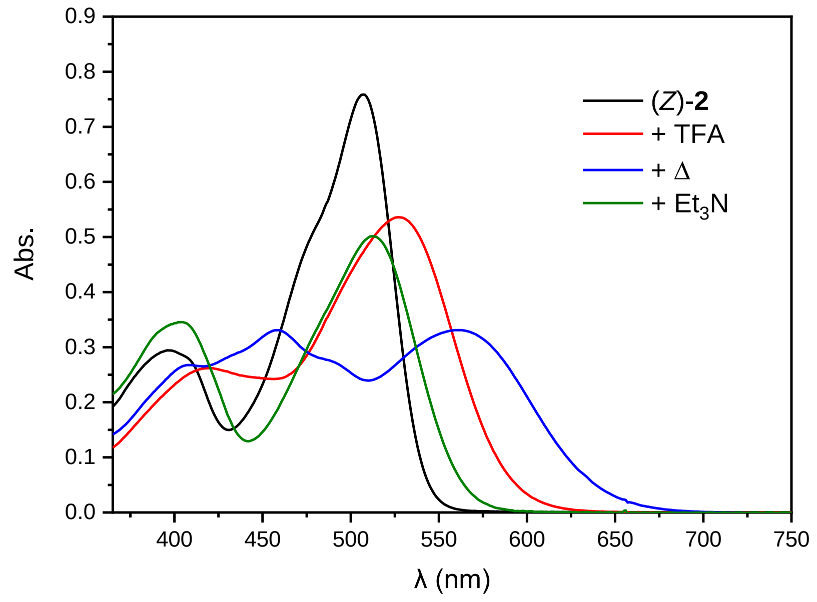


**Figure S55.** UV-Vis spectral data of **2** (0.28 mM in degassed $CHCl_3$, 1 mm quartz cuvette) before and after addition of excess TFA (4.7 × $10^2$ equiv.) [(*Z*)-**2**·**H**$^+$], followed by equilibration for 16 h [Δ, enriched in (*E*)-**2**·H$^+$], and treatment with $Et_3N$ (1.5 equiv. with respect to TFA) [enriched in (*E*)-**2**].

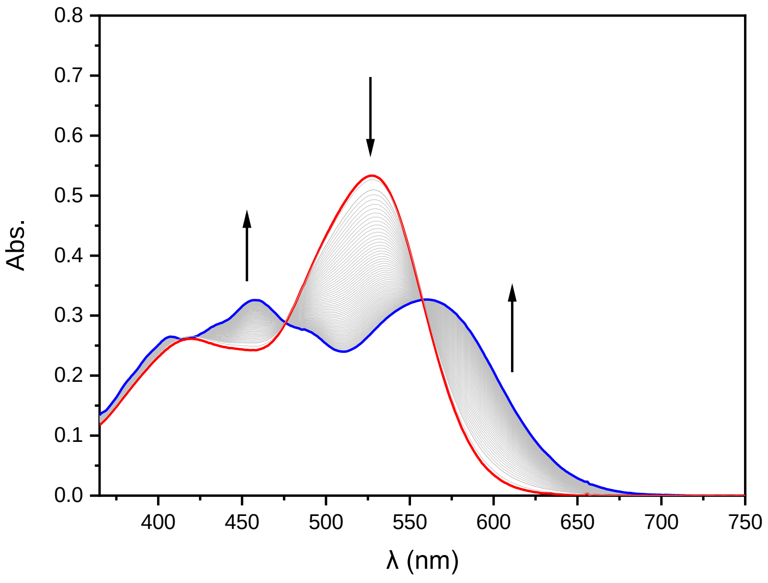


**Figure S56.** UV-Vis spectral changes of (*Z*)-**2** (0.28 mM, $CHCl_3$, 1 mm cuvette) monitored over time (15 min interval) in presence of excess TFA (4.7 × $10^2$ equiv.).

**Acid-induced *Z*→*E* isomerization of 3**

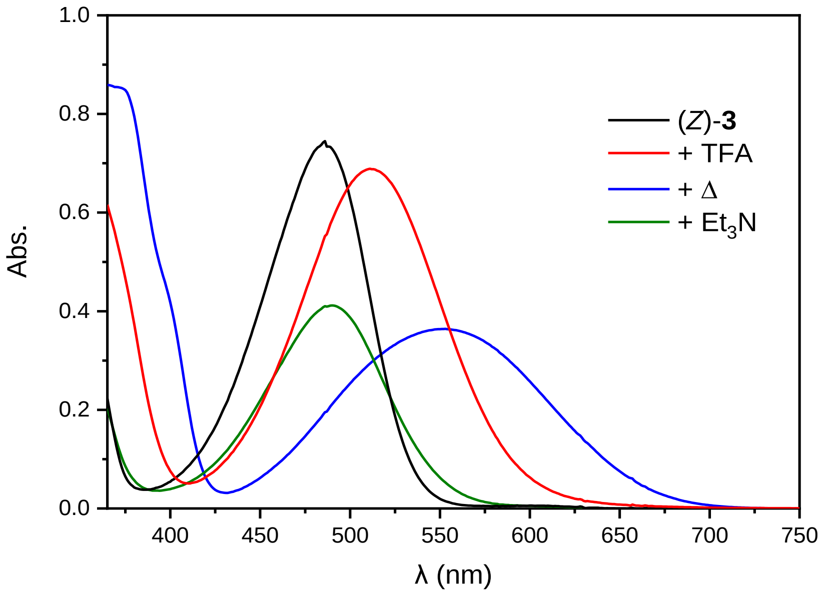


**Figure S57.** UV-Vis spectra data of **3** (0.61 mM in degassed $CHCl_3$, 1 mm quartz cuvette) before and after addition of excess TFA ($4.7 \times 10^2$ equiv.) [(*Z*)-**3**·**H**$^+$], followed by equilibration for 16 h [Δ, enriched in (*E*)-**3**·**H**$^+$] and treatment with $Et_3N$ (1.0 equiv. with respect to TFA) [enriched in (*E*)-**3**].

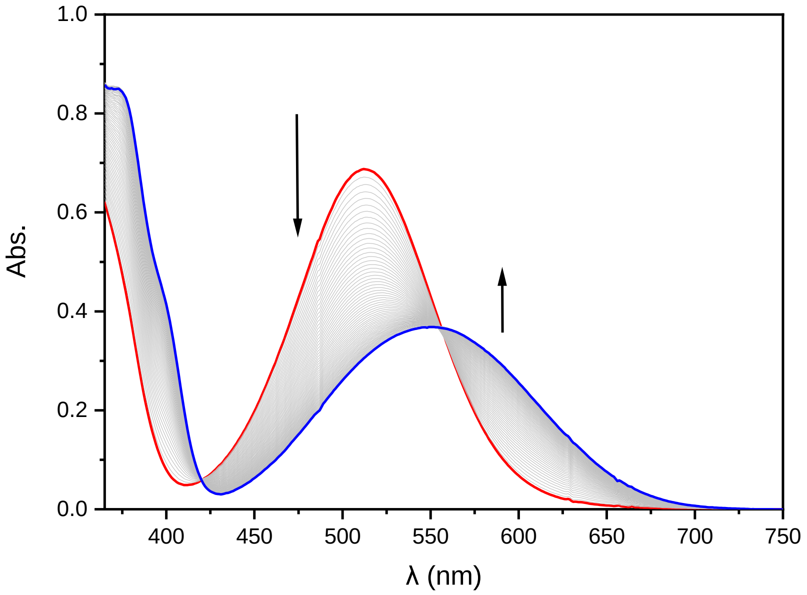


**Figure S58**. UV-Vis spectral changes of (*Z*)-**3** (0.61 mM, $CHCl_3$, 1 mm cuvette) monitored over time (15 min interval) in presence of excess TFA ($4.7 \times 10^2$ equiv.).

## Acid-induced *Z*→*E* isomerization of 4

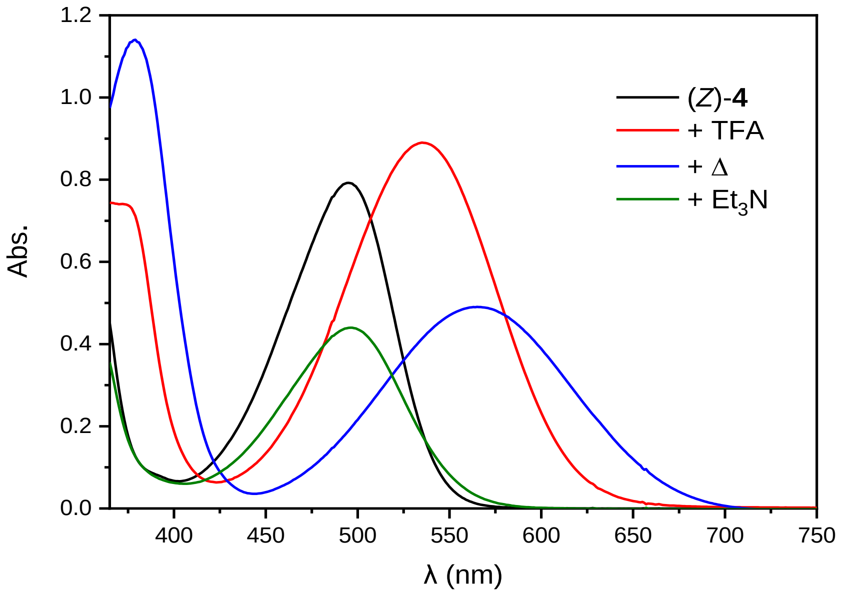


**Figure S59.** UV-Vis spectra data of **4** (0.64 mM in degassed $CHCl_3$, 1 mm quartz cuvette) before and after addition of excess TFA (4.3 × $10^2$ equiv.) [(*Z*)-**4**·**H**$^+$], followed by equilibration for 16 h [Δ, enriched in (*E*)-**4**·**H**$^+$], and treatment with $Et_3N$ (4.0 equiv. with respect to TFA) [enriched in (*E*)-**4**].

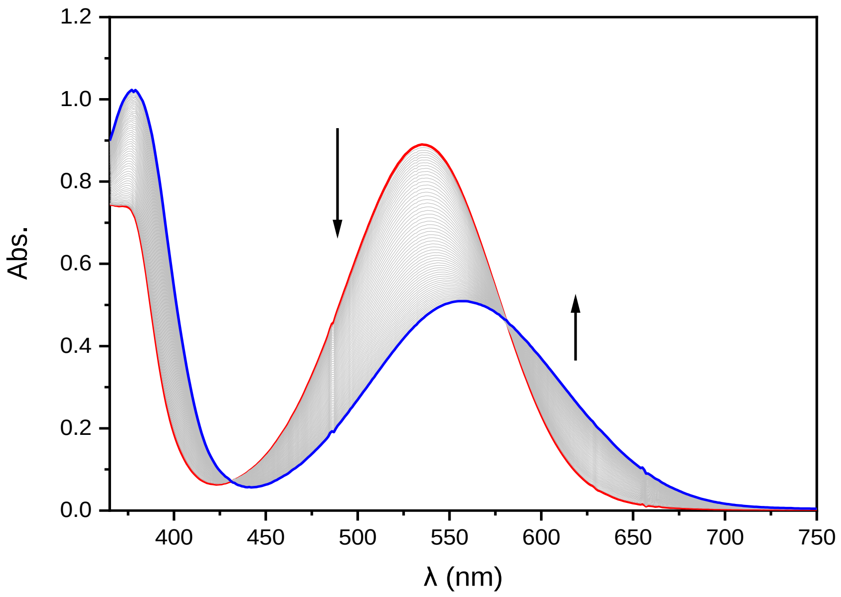


**Figure S60.** UV-Vis spectral changes of (*Z*)-**4** (0.64 mM, $CHCl_3$, 1 mm cuvette) monitored over time (15 min interval) in presence of excess TFA (4.3 × $10^2$ equiv.).

**Acid-induced *Z*→*E* isomerization of 5**

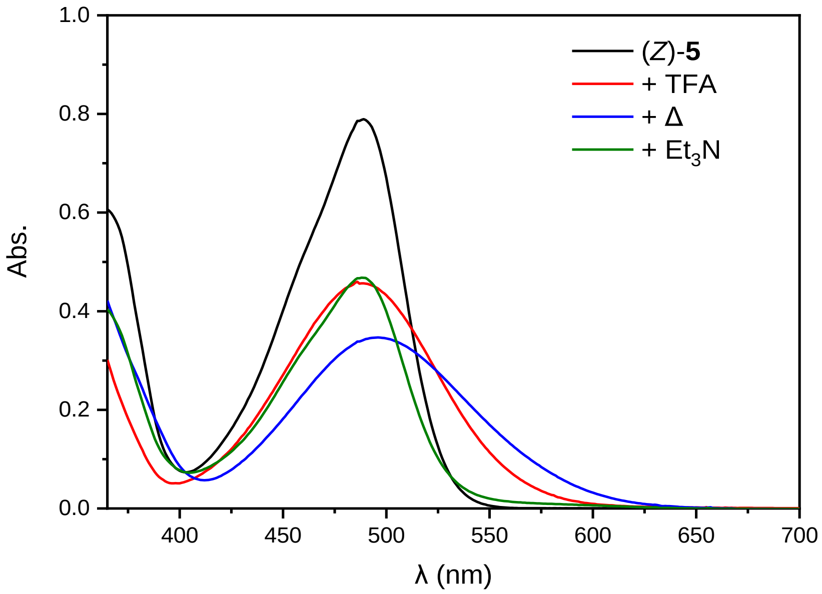


**Figure S61.** UV-Vis spectra data of **5** (0.71 mM in degassed $CHCl_3$, 1 mm quartz cuvette) before and after addition of excess TFA (3.7 × $10^2$ equiv.) [(*Z*)-**5**·**H**$^+$], followed by equilibration for 16 h [Δ, enriched in (*E*)-**5**·**H**$^+$], and treatment with $Et_3N$ (4.2 equiv. with respect to TFA) [enriched in (*E*)-**5**].

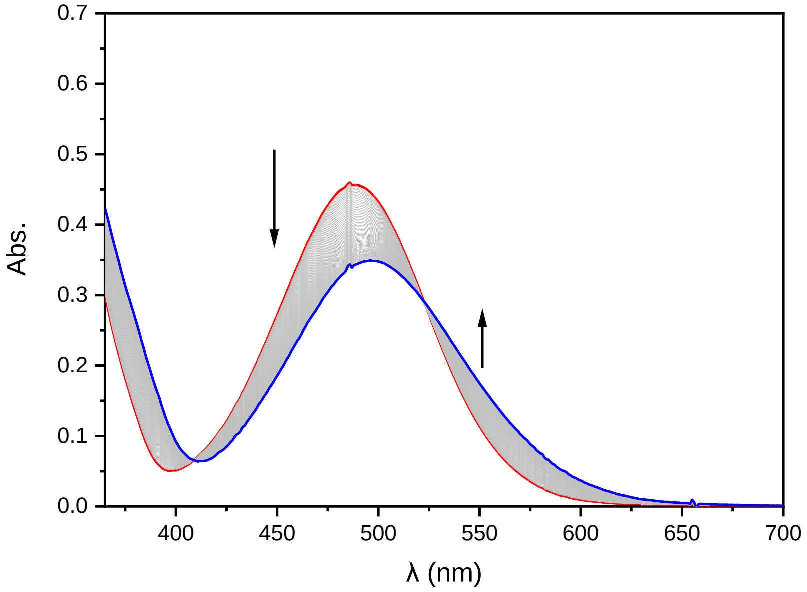


**Figure S62.** UV-Vis spectral changes of (*Z*)-**5** (0.71 mM, $CHCl_3$, 1 mm cuvette) monitored over time (15 min interval) in presence of excess TFA (3.7 × $10^2$ equiv.).

**Acid-induced *Z*→*E* isomerization of 6**

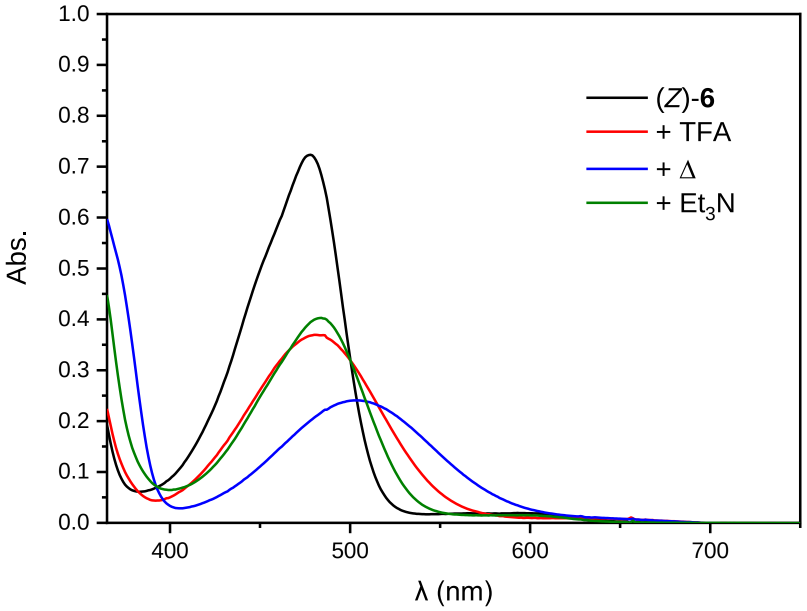


**Figure S63.** UV-Vis spectra data of **6** (0.89 mM in degassed $CHCl_3$, 1 mm quartz cuvette) before and after addition of excess TFA (3.7 × $10^2$ equiv.) [(*Z*)-**6**·**H**$^+$], followed by equilibration for 16 h [Δ, enriched in (*E*)-**6**·**H**$^+$], and treatment with $Et_3N$ (4.2 equiv. with respect to TFA) [enriched in (*E*)-**6**].

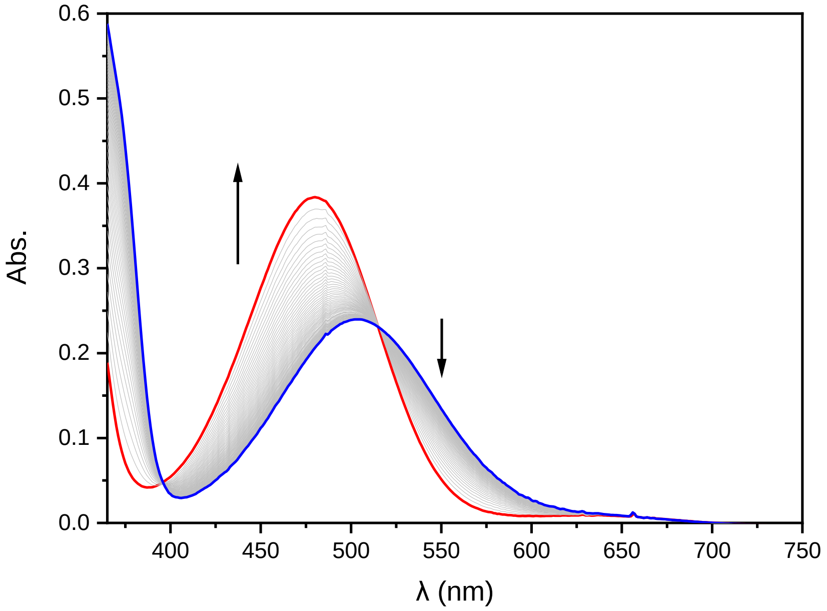


**Figure S64.** UV-Vis spectral changes of (*Z*)-**6** (0.89 mM, $CHCl_3$, 1 mm cuvette) monitored over time (15 min interval) in presence of excess TFA (3.7 × $10^2$ equiv.).

## 8. UV-Vis photoisomerization studies

All UV-Vis spectra were recorded at 273 K (or lower if specified) to minimize the effect of thermal isomerization. Samples of (*E*)-isomers were prepared from the (*Z*)-isomer by irradiation in presence of TFA or alternatively in the case of compound **6**, by thermal equilibration in the presence of excess TFA (left overnight) at the same concentration as at which the $^1$H NMR experiments were performed.

**Note:** In comparison to the photochemical isomerization, it appears that lower conversion was achieved upon thermal isomerization in the UV-Vis experiments (as described in Section 7) than in the $^1$H NMR experiments (as described in section 5 & 6). For example, for compound **1** (Figure S65), the spectrum for the photochemically generated species [(*E*)-**1**·**H**$^+$] is further red-shifted than was observed after the thermally activated process ($\lambda_{max,thermal}$ = 549 nm vs $\lambda_{max,photochemical}$ = 566 nm vs $\lambda_{max,TD\text{-}DFT}$ = 554 nm). The thermal isomerization process may be affected by incomplete protonation, the different nature of the pyridinium-carboxylate ion pair and/or the increased dielectric constant of the medium in presence of the larger excess of TFA necessary for protonation at the lower concentration used in the UV-Vis experiments.

## Photoisomerization of 1

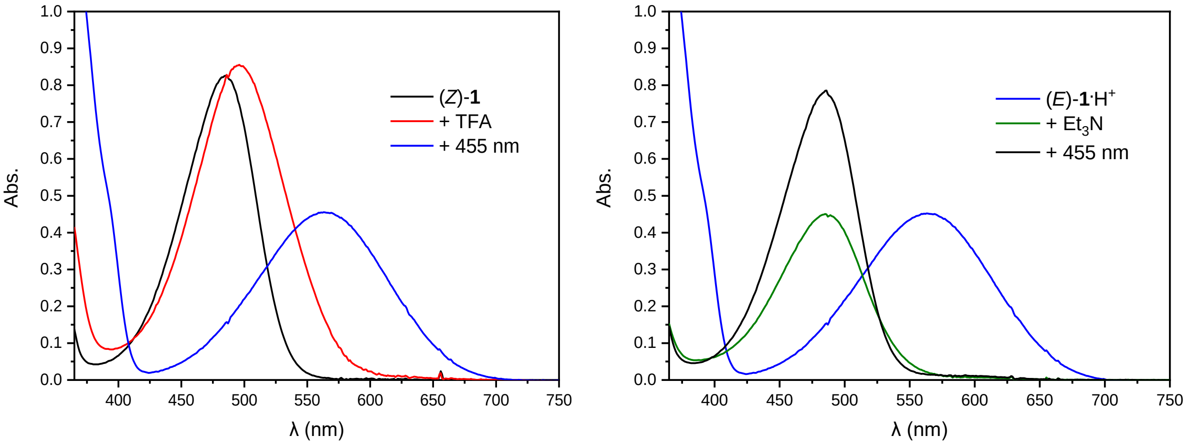


**Figure S65.** (left) UV-Vis spectra starting with (*Z*)-**1** (0.076 mM in degassed $CHCl_3$, 1 cm quartz cuvette), where irradiation with 365 nm, 385 nm, 455 nm, 465 nm, 525 nm, for 2-10 min did not cause any spectral changes, yet after addition of TFA ($4.7 \times 10^2$ equiv.) and irradiation with 455 nm photoisomerization occurred. (right) UV-Vis spectrum of (*E*)-**1·H**$^+$ (0.076 mM in degassed $CHCl_3$, formed by 455 nm irradiation at a concentration of 0.076 mM in presence of $4.7 \times 10^2$ eq TFA), where irradiation with 365 nm, 385 nm, 455 nm, 465 nm, 525 nm, 591 nm, 630 nm, and 660 nm for 2-10 min did not cause any further spectral changes. After addition of $Et_3N$ ($7.0 \times 10^2$ equiv.) and irradiation at 455 nm photoisomerization to (*Z*)-**1** took place.

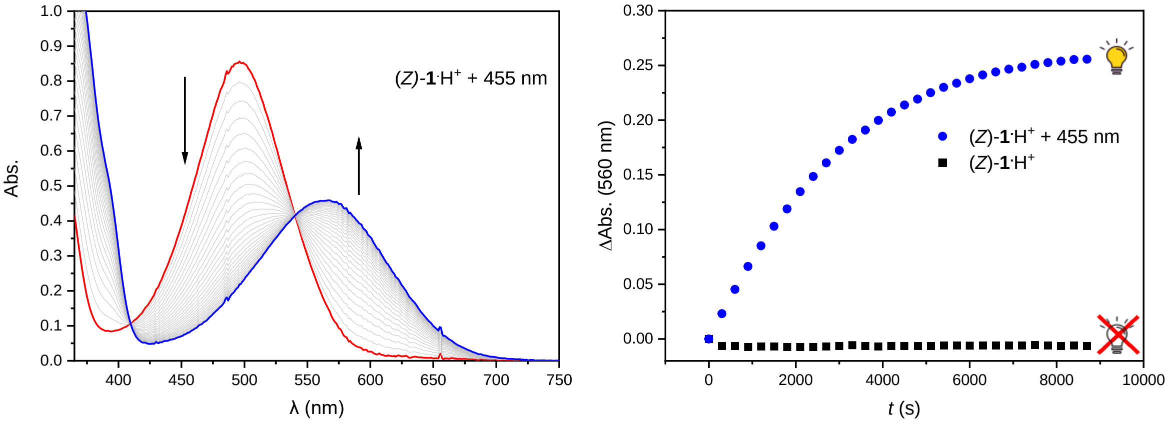


**Figure S66.** (left) UV-Vis spectral changes of ($Z$)-**1·H**$^+$ upon continuous irradiation at 455 nm, and (right) change in absorbance of ($Z$)-**1·H**$^+$ at 560 nm with and without of irradiation. In the latter case, virtually no change in absorbance was observed, showing that thermal isomerization is negligible under the experimental conditions used [as described in Figure **S65** (left)].

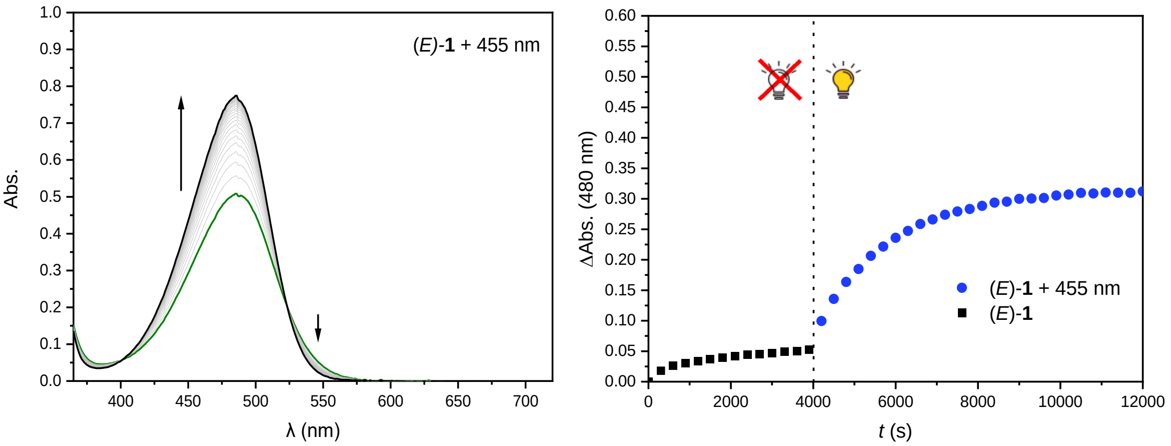


**Figure S67.** (Left) UV-Vis spectral changes of (*E*)-**1** under continuous irradiation at 455 nm, and (right) changes in absorbance of (*E*)-**1** at 480 nm with and without irradiation. In the latter case minor thermal isomerization is observed under the experimental conditions used [as described in Figure **S65** (right)].

## Photoisomerization of 2

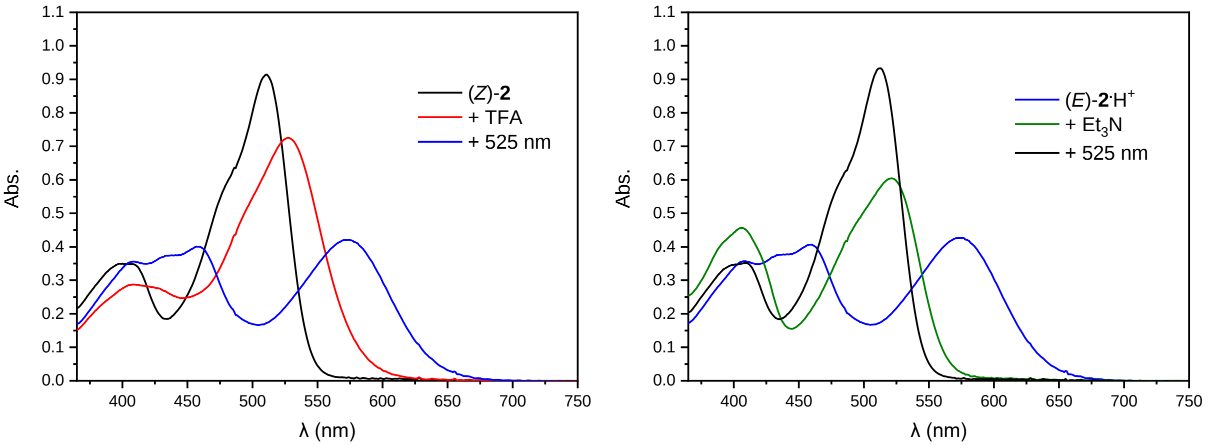


**Figure S68.** (left) UV-Vis spectra starting with (*Z*)-**2** (0.028 mM in degassed $CHCl_3$, 1 cm quartz cuvette), where irradiation with 405 nm, 455 nm, 465 nm, 525 nm for 6 min did not cause any spectral changes, yet after addition of TFA ($4.7 \times 10^2$ equiv.) and irradiation with 525 nm photoisomerization occurred. (right) UV-Vis spectrum of (*E*)-**2·H**$^+$ at 263 K (0.028 mM in degassed $CHCl_3$, formed by irradiation using 525 nm light at a concentration of 0.028 mM in presence of $4.7 \times 10^2$ equiv. TFA), where irradiation with 405 nm, 455 nm, 525 nm, 591 nm, 630 nm or 660 nm for 6 min did not cause any further spectral changes. After addition of $Et_3N$ ($7.0 \times 10^2$ equiv.) and irradiation at 525 nm photoisomerization to (*Z*)-**2** took place.

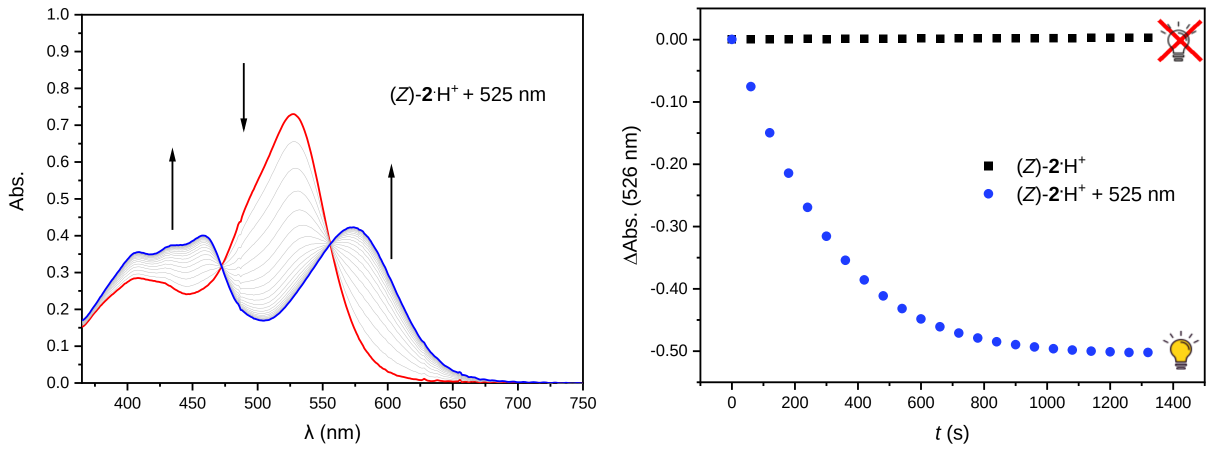


**Figure S69.** (left) UV-Vis spectral changes of (*Z*)-**2·H**$^+$ upon continuous irradiation at 525 nm, and (right) change in absorbance of (*Z*)-**2·H**$^+$ at 526 nm with and without irradiation. In the latter case, virtually no change in absorbance was observed, showing that thermal isomerization is negligible under the experimental conditions and concentrations used [as described in Figure **S68** (left)].

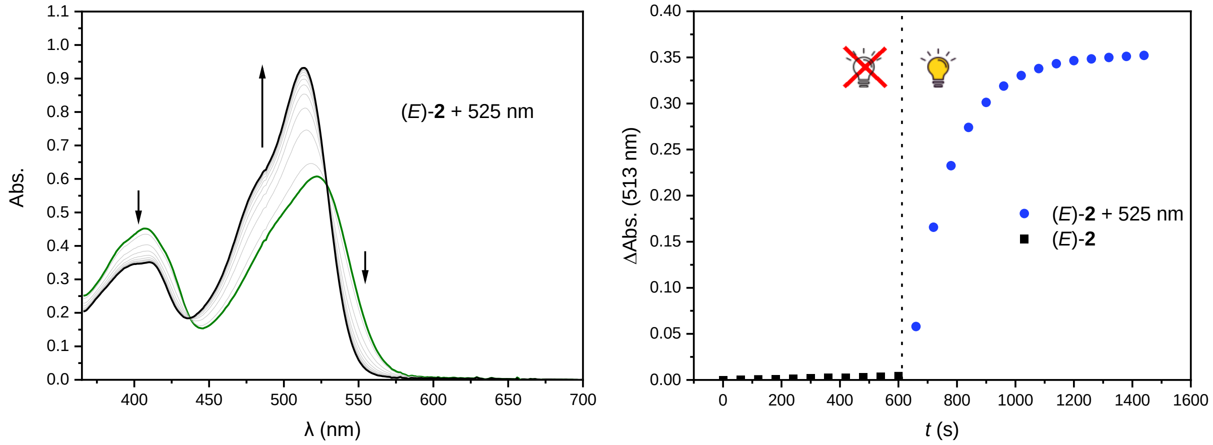


**Figure S70**. (left) UV-Vis spectral changes of (*E*)-**2** under continuous irradiation at 525 nm, and (right) changes in absorbance of (*E*)-**2** at 513 nm with and without irradiation. In the latter case, virtually no change in absorbance was observed, showing that thermal isomerization is negligible under the experimental conditions used [as described in Figure **S68** (right), T = 263 K].

## Photoisomerization of 3

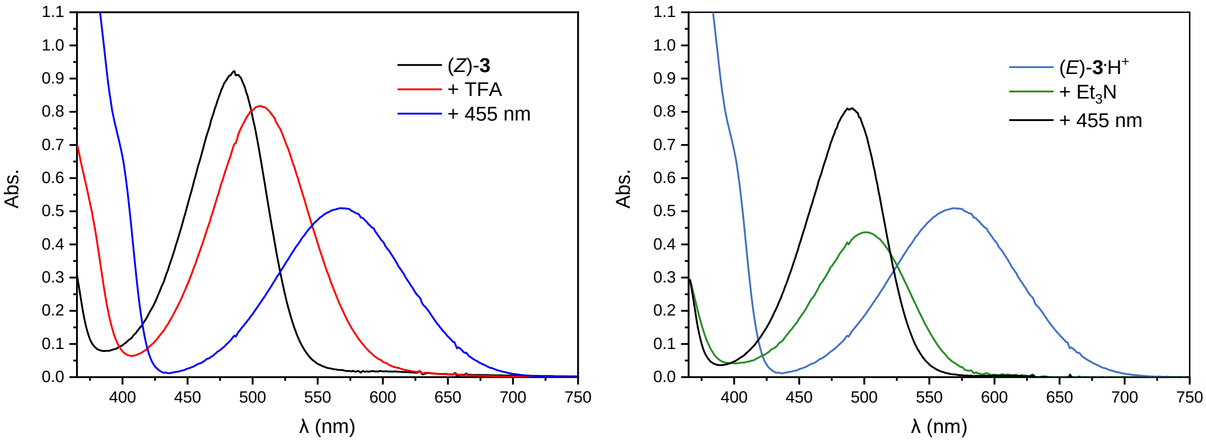


**Figure S71.** (left) UV-Vis spectra starting with (*Z*)-**3** (0.073 mM in degassed $CHCl_3$, 1 cm quartz cuvette), where irradiation with 405 nm, 455 nm, 525 nm, or 591 nm for 2-10 min did not cause any spectral changes, yet after addition of TFA ($2.4 \times 10^3$ equiv.) and irradiation with 455 nm photoisomerization occurred. (right) UV-Vis spectrum of (*E*)-**3·H**$^+$ (0.073 mM in degassed $CHCl_3$, formed by irradiation using 455 nm light at a concentration of 0.073 mM in presence of $2.4 \times 10^3$ equiv. TFA), where irradiation with 405 nm, 455 nm, 525 nm, or 591 nm for 2-10 min did not cause any further spectral changes. After addition of $Et_3N$ ($3.7 \times 10^3$ equiv.) and irradiation with 525 nm photoisomerization to (*Z*)-**3** took place.

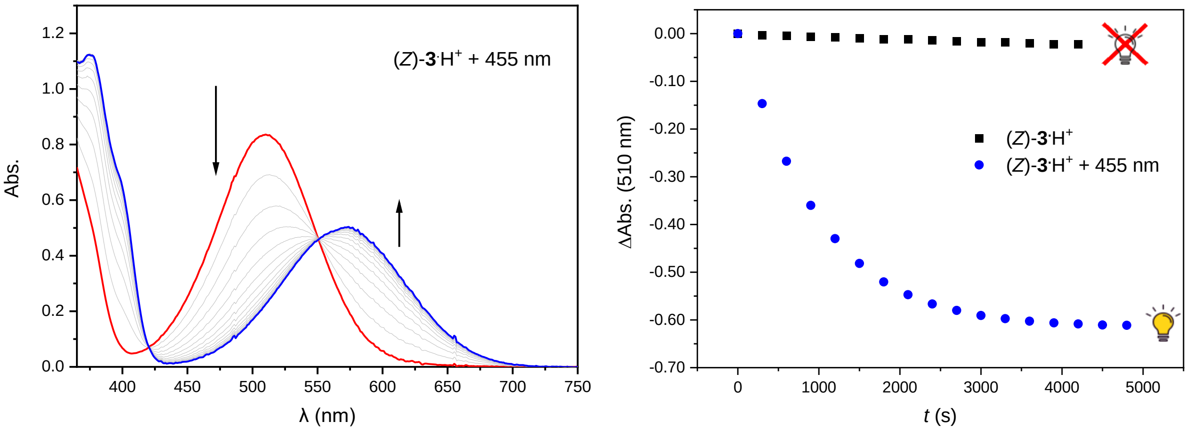


**Figure S72.** (left) UV-Vis spectral changes of (*Z*)-**3·H**$^+$ upon continuous irradiation at 455 nm, and (right**)** change in absorbance of (*Z*)-**3·H**$^+$ at 510 nm with and without irradiation. In the latter case, only a minor change in absorbance was observed, showing that thermal isomerization is negligible under the experimental conditions used [as described in Figure **S71** (left)].

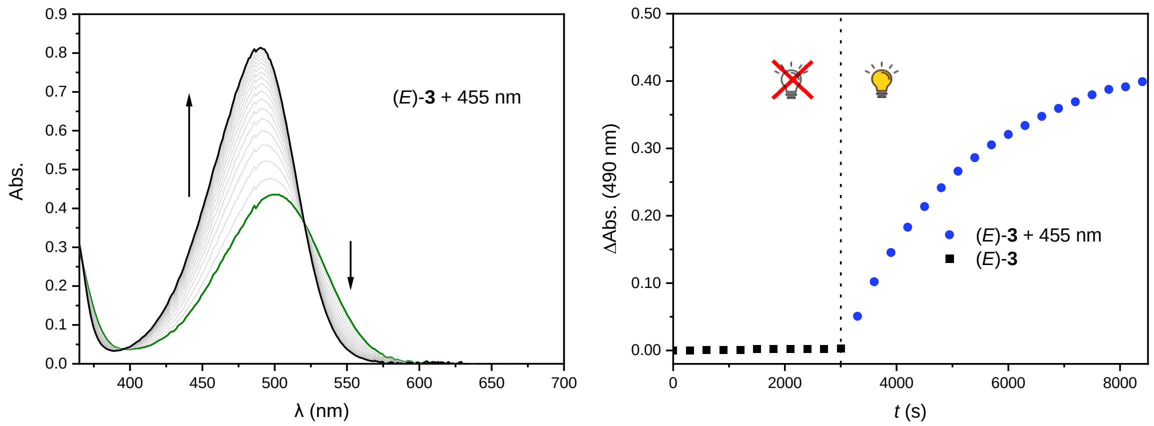


**Figure S73.** (left) UV-Vis spectral changes of (*E*)-**3** under continuous irradiation at 455 nm, and (right) changes in absorbance of (*E*)-**3** at 490 nm with and without irradiation. In the latter case, virtually no change in absorbance was observed, showing that thermal isomerization pathway is negligible under the experimental conditions used [as described in Figure **S71** (right)].

## Photoisomerization of 4

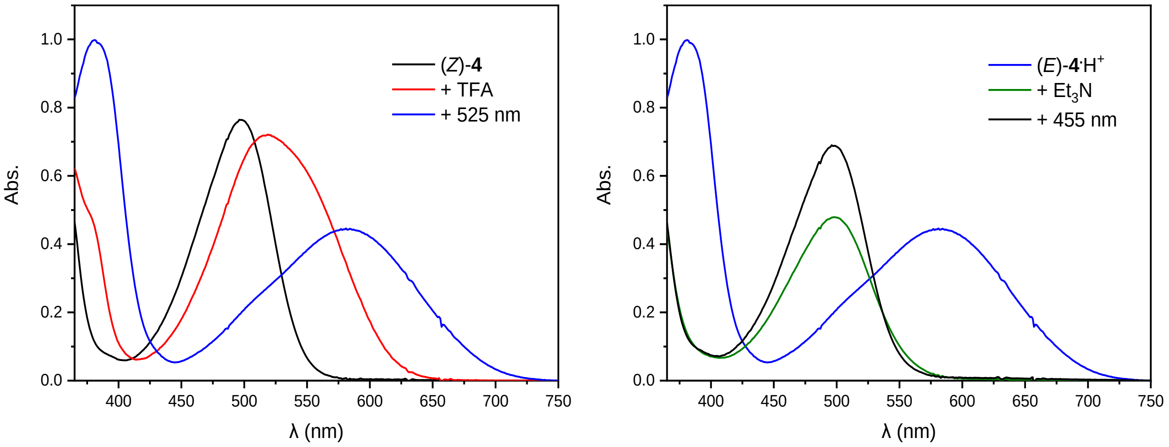


**Figure S74. (**Left) UV-Vis spectra starting with (*Z*)-**4** (0.051 mM in degassed $CHCl_3$, 1 cm quartz cuvette), where irradiation with 405 nm, 455 nm, 465 nm, 525 nm for 2-10 min did not cause any spectral changes, yet after addition of TFA ($4.7 \times 10^2$ equiv.) and irradiation with 525 nm photoisomerization occurred. (right) UV-Vis spectrum of (*E*)-**4·H**$^+$ at 263 K (0.051 mM in degassed $CHCl_3$, formed by irradiation using 525 nm light at a concentration of 0.051 mM in presence of $4.7 \times 10^2$ equiv. TFA), where irradiation with 455 nm, 525 nm and 591 nm for 2-10 min did not cause in any further spectral changes. After addition of $Et_3N$ ($7.0 \times 10^2$ equiv.) and irradiation with 455 nm photoisomerization to (*Z*)-**4** took place.

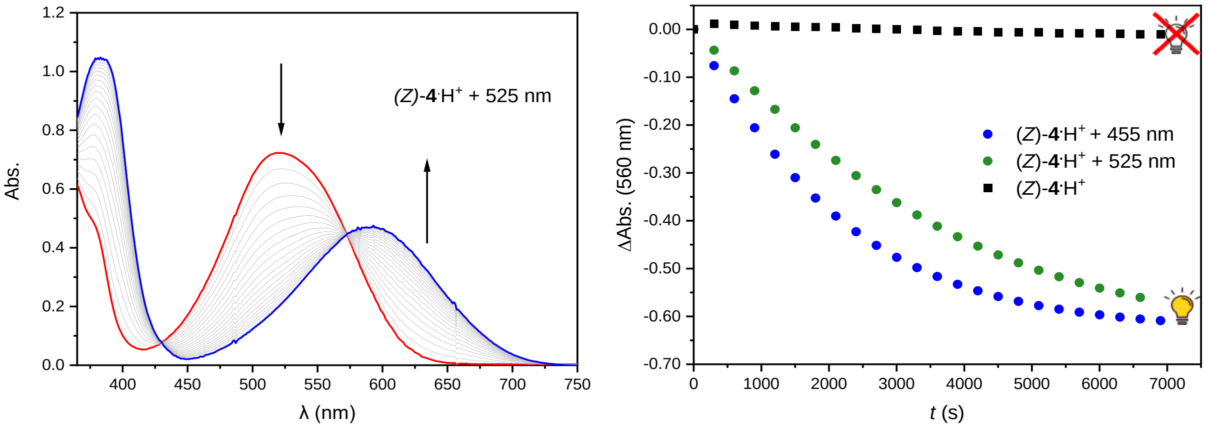


**Figure S75.** (left) UV-Vis spectral changes of (*Z*)-**4·H**$^+$ upon continuous irradiation at 525 nm, and (right**)** change in absorbance of (*Z*)-**4·H**$^+$ at 560 nm with and without irradiation. In the latter case, virtually no change in absorbance was observed, showing that thermal isomerization is negligible under the experimental conditions used [as described in Figure **S74** (left)].

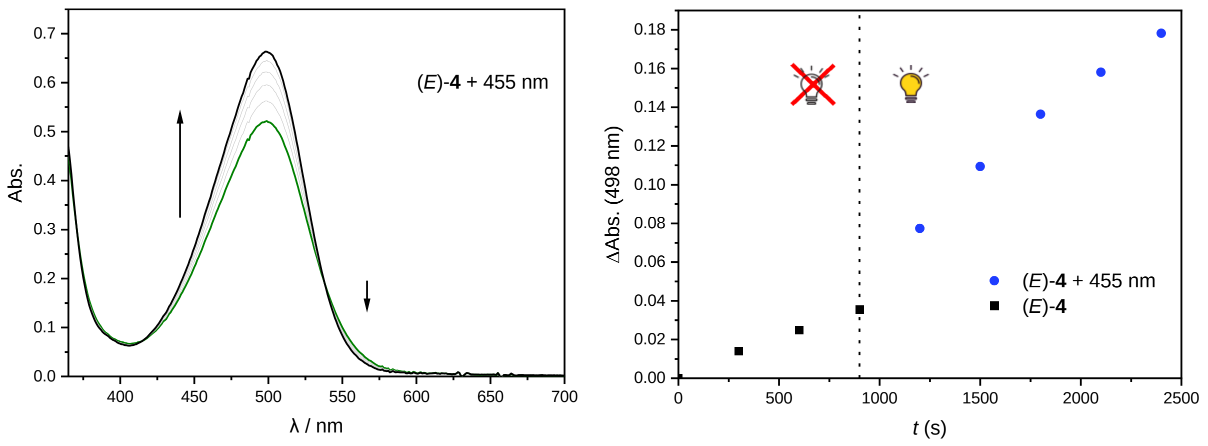


**Figure S76.** (left) UV-Vis spectral changes of (*E*)-**4** under continuous irradiation at 455 nm, and (right) changes in absorbance of (*E*)-**4** at 498 nm with and without irradiation. While thermal isomerization is observed under the experimental conditions used [as described in Figure **S74** (right), T = 263 K), conversion to (*E*)-**4** is clearly accelerated by light.

## Photoisomerization of 5

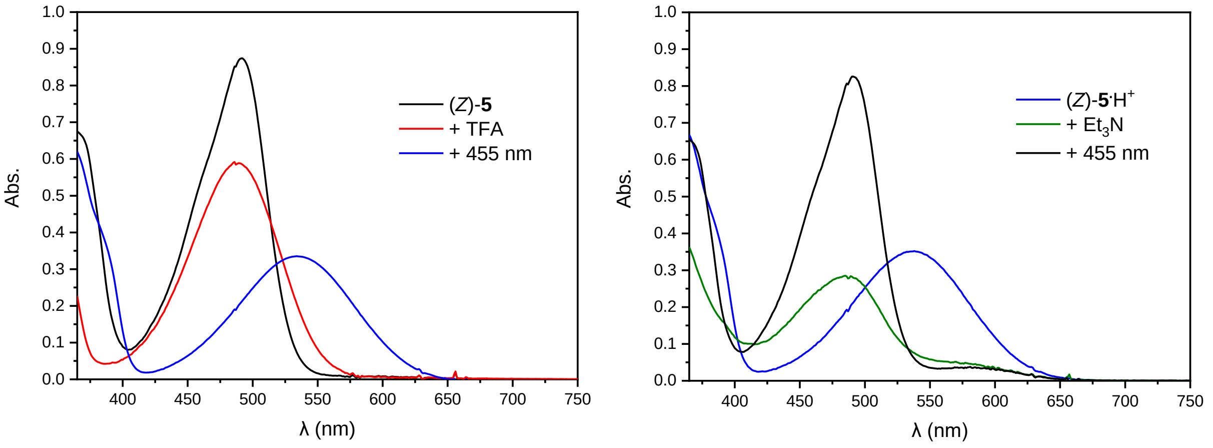


**Figure S77.** (left) UV-Vis spectra starting with (*Z*)-**5** (0.071 mM in degassed $CHCl_3$, 1 cm quartz cuvette), where irradiation with 455 nm, 465 nm, 525 nm for 2-10 min did not cause any spectral changes, yet after addition of TFA (4.7 × $10^2$ equiv.) and irradiation with 455 nm photoisomerization occurred. (right) UV-Vis spectrum of (*E*)-**5·H**$^+$ 263 K (0.071 mM in degassed $CHCl_3$, formed by irradiation using 455 nm light at a concentration of 0.071 mM in presence of 4.7 × $10^2$ equiv. TFA), where irradiation with 455 nm, 465 nm, 525 nm and 591 nm for 2-10 min did not cause in any spectral changes. After addition of $Et_3N$ (2.0 × $10^3$ equiv.) and irradiation with 455 nm photoisomerization to (*Z*)-**5** took place (alternatively 465 nm or 525 nm light could be used).

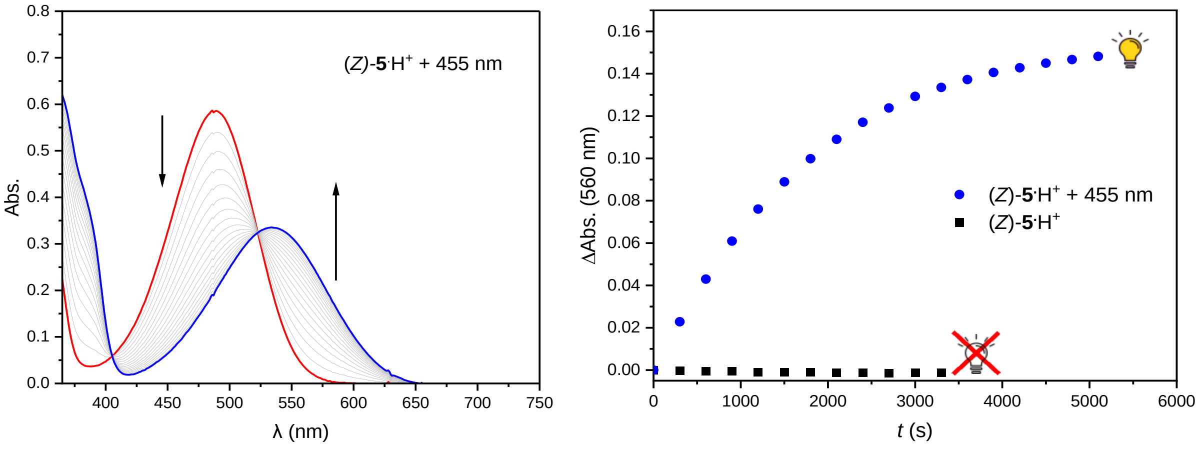


**Figure S78.** (left) UV-Vis spectral changes of (*Z*)-**5·H**$^+$ upon continuous irradiation at 455 nm, and (right) change in absorbance of (*Z*)-**5·H**$^+$ at 560 nm with and without irradiation. In the latter case, virtually no change in absorbance was observed, showing that thermal isomerization is negligible under the experimental conditions used [as described in Figure **S77** (left)].

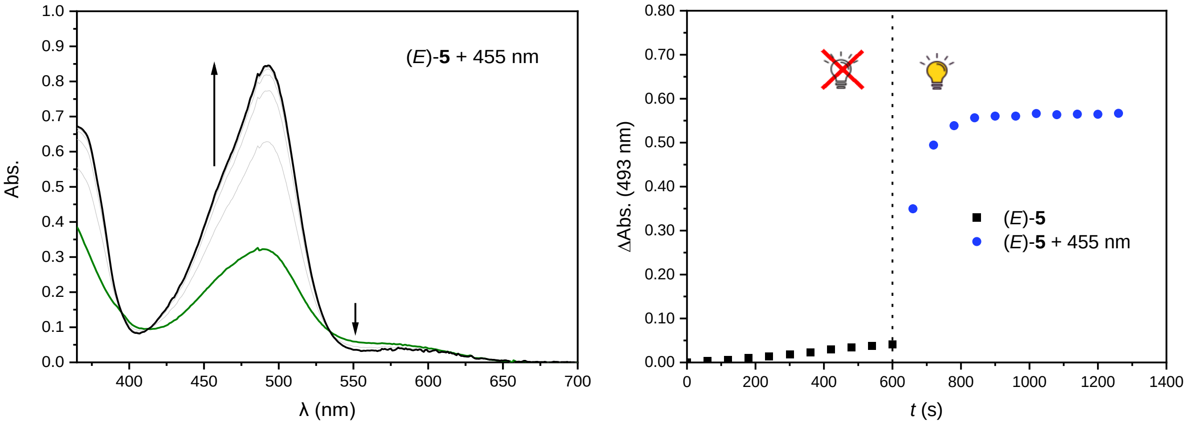


**Figure S79.** (left) UV-Vis spectral changes of (*E*)-**5** under continuous irradiation at 455 nm, and (right) changes in absorbance of (*E*)-**5** at 493 nm with and without irradiation. In the latter case, only minor thermal isomerization is observed under the experimental conditions used.[as described in Figure **S77** (right), T = 263 K).

## Photoisomerization of 6

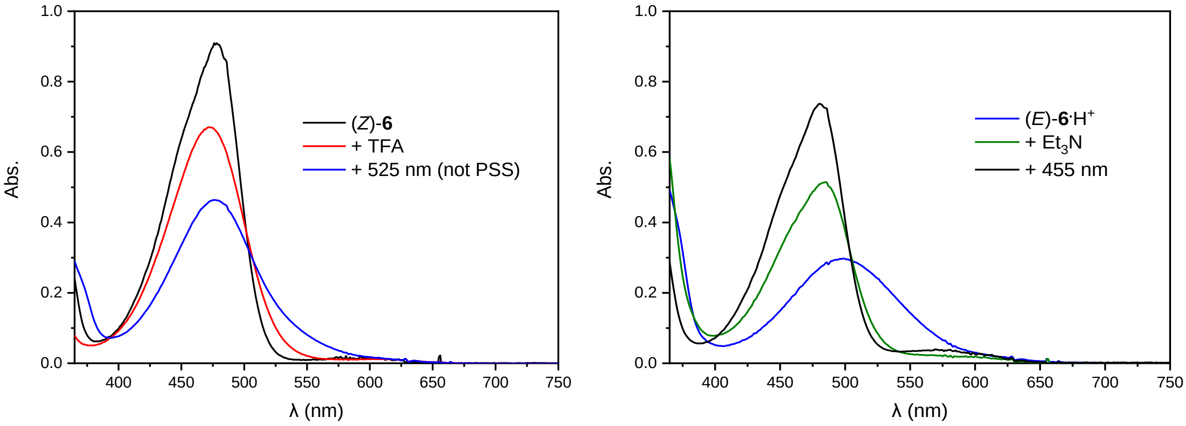


**Figure S80.** (left) UV-Vis spectra starting with (*Z*)-**6** (0.088 mM in degassed $CHCl_3$, 1 cm quartz cuvette), where irradiation with 525 nm for 2-10 min did not cause any spectral changes, but irradiation with 405 and 455 nm for 2-12 min resulted in minor decomposition (<5%, see Figure **S81**). After addition of TFA ($4.7 \times 10^2$ equiv.) and irradiation with 455 nm photoisomerization and decomposition occurred (see Figure **S81**), irradiation with 525 nm afforded clean photoisomerization (see Figure **S82**), however, the PSS was not yet reached after 80 minutes due to slow photoisomerization. (right) UV-Vis spectrum of (*E*)-**6·H**$^+$ (0.088 mM in degassed $CHCl_3$, which was formed by thermal equilibration at 4.8 mM concentration in presence of $4.7 \times 10^2$ eq TFA), where irradiation with 405 nm and 455 nm for 2 min did not cause any significant spectral changes. After addition of $Et_3N$ ($7.0 \times 10^2$ equiv.) and irradiation with 455 nm photoisomerization to (*Z*)-**6** took place.

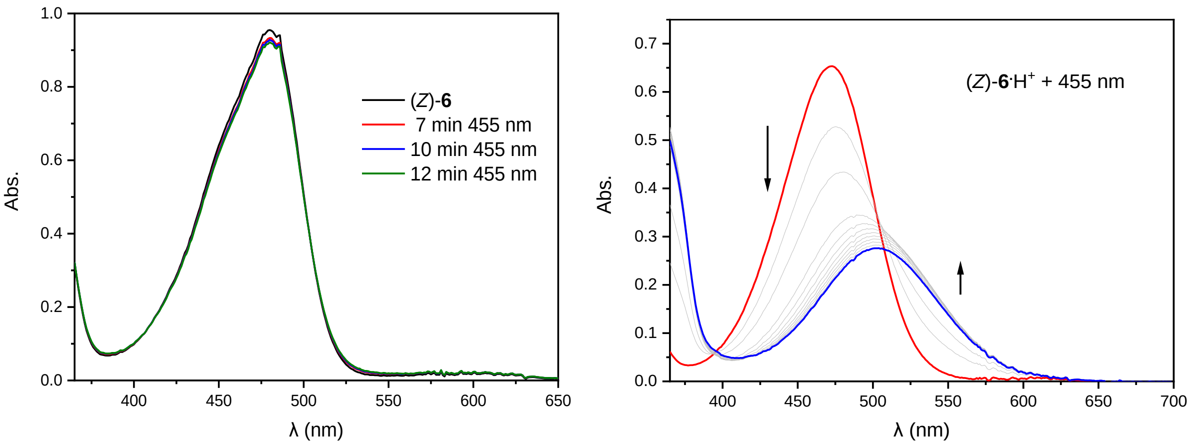


**Figure S81.** (left) UV-Vis spectra of (*Z*)-**6** under continuous irradiation at 455 nm. (right) UV-Vis spectra of (*Z*)-**6·H**$^+$ under continuous irradiation with 455 nm showing concomitant photoisomerization and photodegradation. Spectra were taken every 5 min under the experimental conditions described in Figure **S80** (left).

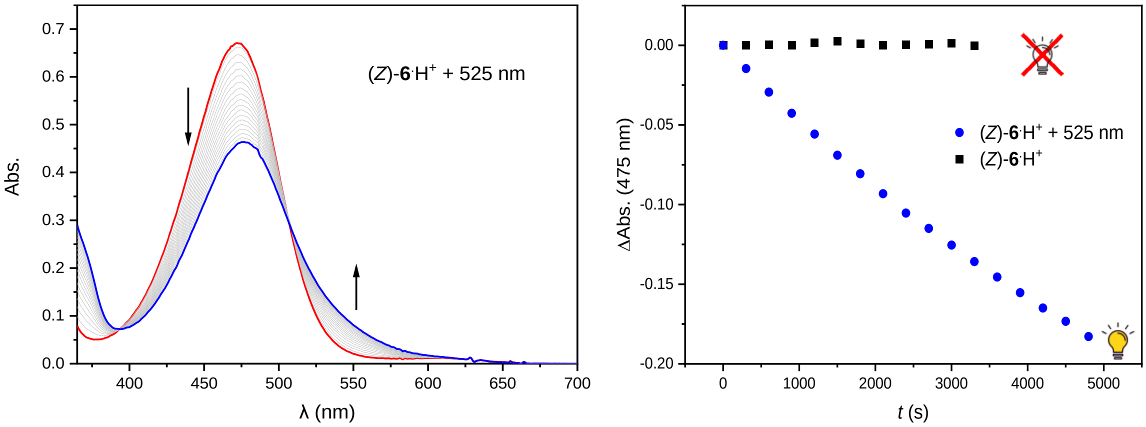


**Figure S82.** (left) UV-Vis spectral changes of (*Z*)-**6·H**$^+$ upon continuous irradiation at 525 nm, and (right) change in absorbance of (*Z*)-**6·H**$^+$ at 475 nm with and without irradiation. In the latter case, virtually no change in absorbance was observed, showing that thermal isomerization is negligible under the experimental conditions used [as described in Figure **S82** (left)].

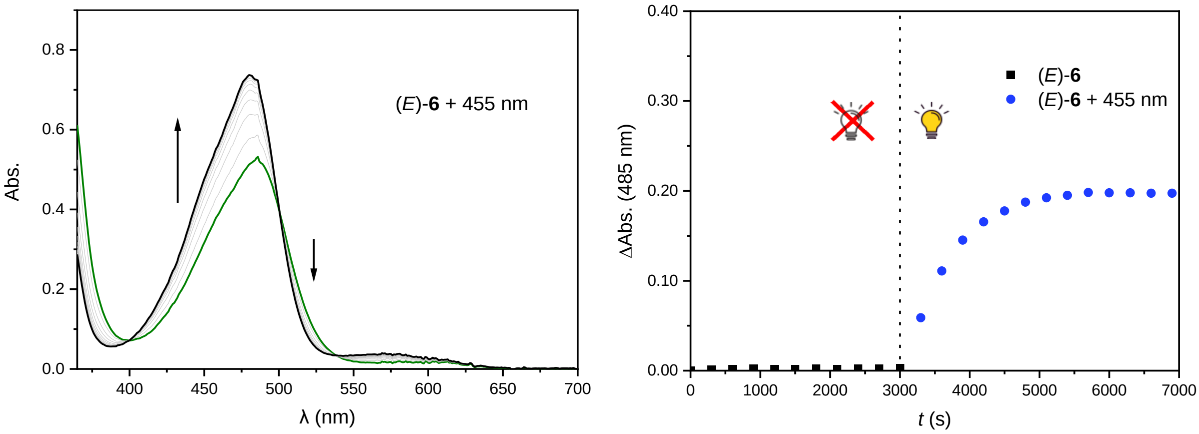


**Figure S83.** (left) UV-Vis spectral changes of (*E*)-**6** under continuous irradiation at 455 nm, and (right) changes in absorbance of (*E*)-**6** at 485 nm with and without irradiation. In the latter case, thermal isomerization is negligible under the experimental conditions used [as described in Figure **S80** (right)].

**Table S8.** Overview of photoresponsive behavior of **1-6** with the wavelengths listed in nanometer. "N" or "Y" indicate the absence or presence of spectral changes indicative of isomerization upon irradiation with the wavelength listed, respectively.

| **Compound** | Wavelength: | 365 | 385 | 405 | 455 | 465 | 525 | 591 | 630 | 660 |
|---|---|---|---|---|---|---|---|---|---|---|
| **1** | $Z \rightarrow E$ | N | N | | N | N | N | | | |
| | $Z{\cdot}H^+ \rightarrow E{\cdot}H^+$ | | | | Y | Y | | | | |
| | $E{\cdot}H^+ \rightarrow Z{\cdot}H^+$ | N | N | | N | N | N | N | N | N |
| | $E \rightarrow Z$ | | | | Y | | | | | |
| **2** | $Z \rightarrow E$ | | | N | N | N | N | | | |
| | $Z{\cdot}H^+ \rightarrow E{\cdot}H^+$ | | | | Y | | Y | | | |
| | $E{\cdot}H^+ \rightarrow Z{\cdot}H^+$ | | | N | N | | N | N | N | N |
| | $E \rightarrow Z$ | | | | Y | | Y | | | |
| **3** | $Z \rightarrow E$ | | | N | N | | N | N | | |
| | $Z{\cdot}H^+ \rightarrow E{\cdot}H^+$ | | | | Y | | | | | |
| | $E{\cdot}H^+ \rightarrow Z{\cdot}H^+$ | | | N | N | | N | N | | |
| | $E \rightarrow Z$ | | | | Y | | | | | |
| **4** | $Z \rightarrow E$ | | | N | N | N | N | | | |
| | $Z{\cdot}H^+ \rightarrow E{\cdot}H^+$ | | | | Y | | Y | | | |
| | $E{\cdot}H^+ \rightarrow Z{\cdot}H^+$ | | | | N | | N | N | | |
| | $E \rightarrow Z$ | | | | Y | | | | | |
| **5** | $Z \rightarrow E$ | | | | N | N | N | | | |
| | $Z{\cdot}H^+ \rightarrow E{\cdot}H^+$ | | | | Y | | | | | |
| | $E{\cdot}H^+ \rightarrow Z{\cdot}H^+$ | | | | N | N | N | N | | |
| | $E \rightarrow Z$ | | | | Y | Y | Y | | | |
| **6** | $Z \rightarrow E$ | | | N[a] | N[a] | | N | | | |
| | $Z{\cdot}H^+ \rightarrow E{\cdot}H^+$ | | | | Y[a] | | Y | | | |
| | $E{\cdot}H^+ \rightarrow Z{\cdot}H^+$ | | | N | N[a] | | | | | |
| | $E \rightarrow Z$ | | | | Y | | | | | |

[a] Spectral changes were ascribed to some decomposition.

**Table S9.** Overview of UV-Vis absorption maxima of **1-6** (see section 8) alongside wavelengths that could be used for photoisomerization.

| | *Z* $\lambda_{max}$ = | *Z*·H$^+$ $\lambda_{max}$ = | *Z*·H$^+$→*E*·H$^+$ $\lambda_{irrad}$ = | *E*·H$^+$ $\lambda_{max}$ = | *E*[b] $\lambda_{max}$ = | *E*→*Z* $\lambda_{irrad}$ = |
|---|---|---|---|---|---|---|
| **1** | 485 nm | 496 nm | 455nm | 566 nm | 486 nm | 455 nm |
| **2** | 511 nm | 528 nm | 525 nm | 573 nm | 521 nm | 525 nm |
| **3** | 486 nm | 507 nm | 455 nm | 567 nm | 500 nm | 455 nm |
| **4** | 496 nm | 519 nm | 455 nm | 581 nm | 498 nm | 455 nm |
| **5** | 490 nm | 486 nm | 455 nm | 538 nm | 489 nm | 455 nm |
| **6** | 476 nm | 472 nm | 525 nm | 499 nm[a] | 485 nm[a] | 455 nm |

[a)](*E*)-**6·H**$^+$ thermally generated due to exceedingly slow photoisomerization. [b)]Note that partial thermal isomerization upon exothermic basification to the *Z*-isomer cannot be excluded

# 9. $^{1}$H NMR photoisomerization studies

## Photoisomerization of (*Z*)-1·H$^{+}$

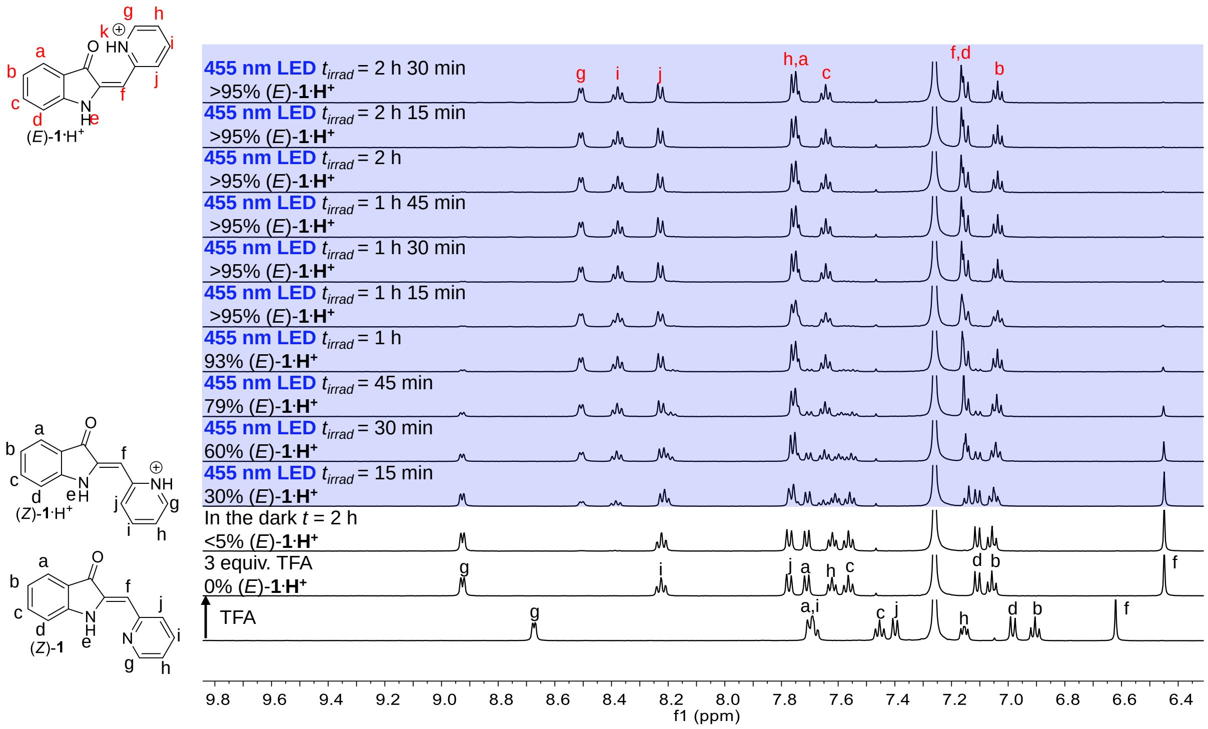


**Figure S84.** $^{1}$H NMR (500 MHz) spectra showing photochemical generation of (*E*)-**1·H**$^{+}$ from (*Z*)-**1·H**$^{+}$ (5.6 mM, $CDCl_3$) in presence of TFA (3.0 eq) by irradiation of the solution in an NMR tube equipped with a J Young valve (to avoid TFA evaporation) with 455 nm. Less TFA is used here than in the thermal isomerization experiments (32 equiv.) to lower thermal conversion to (*E*)-**1·H**$^{+}$ and reduce the rate of this process. Prior to irradiation the sample is kept in the dark for 2 h to confirm that thermal isomerization is negligible.

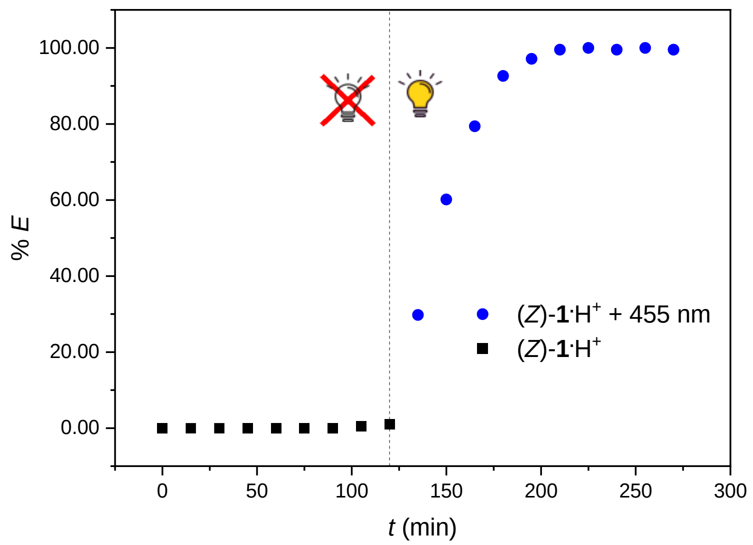


**Figure S85.** Percentage of (*E*)-**1·H**$^+$ (see Figure **S84**) upon irradiation of (*Z*)-**1·H**$^+$ (5.6 mM, $CDCl_3$) in presence of TFA (3.0 equiv.). Minimal thermal conversion takes place in the dark, yet quantitative photoisomerization occurs upon irradiation with 455 nm light.

## 10. Quantum yield determination

The photon flux of the used Thorlabs model M455F3 high-power LED ($\lambda_{max}$ = 455 nm) was determined at maximum power by measuring the production of ferrous ions from potassium ferrioxalate.[8] The number of moles of $Fe^{2+}$ ions produced was determined at four different irradiation times, and the slope of a plot of the molar amount of $Fe^{2+}$ ions versus time (Figure **S86**) corresponds to the rate of $Fe^{2+}$ ion formation, which was found to be $4.46 \times 10^{-8}$ mol $s^{-1}$.

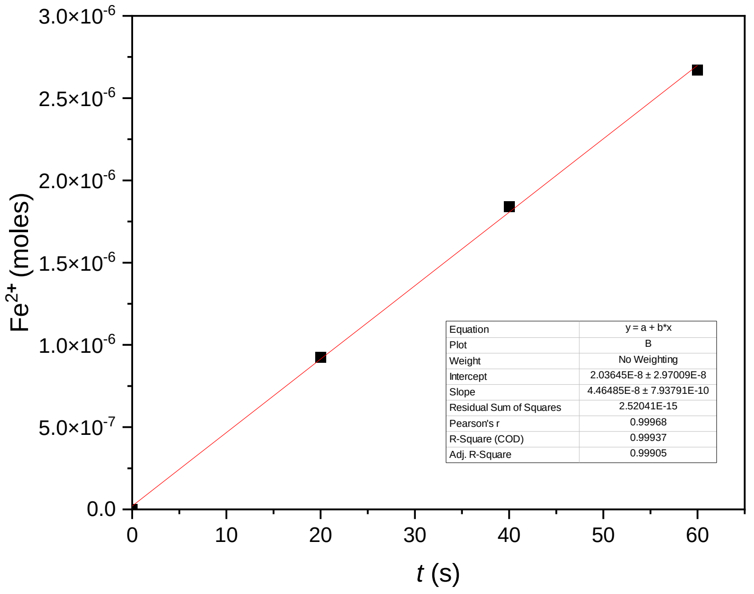


**Figure S86.** Moles of $Fe^{2+}$ ions produced after irradiation of a 0.15 M ferrioxalate solution at 455 nm in a 1 cm quartz cuvette at 293 K and linear data fitting to determine the slope.

The rate of $Fe^{2+}$ ion formation was used to calculate the photon flux with the equation:[8,9]

$$\text{Photon flux (mol s}^{-1}\text{)} = \frac{dFe^{2+}}{dt} \times \frac{1}{\phi \times F}$$

Where the closest reported quantum yield of ferrioxalate ($\phi$ = 1.09 at 457.9 nm)[10] was taken and the fraction of light absorbed by the ferrioxalate solution is given by $F = 1 - 10^{-\mathrm{Abs}(455\ \mathrm{nm})}$.[8] Using these values (F=0.957 in this case), the photon flux was calculated as $4.28 \times 10^{-8}$ mol $s^{-1}$.

Photoisomerization of compounds **1-5** was followed in the same irradiation set-up as the ferrioxalate solution and the rates of new isomer formation and the previously determined photon flux were used to calculate the quantum yields by the following equation:

$$\phi = \frac{\text{Rate of isomer formation}}{\text{Photon flux}}$$

The quantum yield of ($Z$)·$\mathbf{H}^+$→($E$)·$\mathbf{H}^+$ photoisomerization was determined by first preparing solutions of the respective ($Z$)-isomer in degassed $CHCl_3$ and followed by the addition of 470 equiv. of TFA at 0° C. The concentrations were such that all incoming light was absorbed (Abs ≥ 2.0 at 455 nm). Upon irradiation with 455 nm light, the change in absorbance was followed at a wavelength at which the absorbance is linear with concentration (Abs < 1.0). This change in absorbance was then used to calculate the concentration increase of ($E$)-isomer, by using the known molar absorptivity ($\varepsilon$) at the selected wavelength (see Table **S10** for an overview).

$$\Delta[(\boldsymbol{E})\cdot\mathbf{H}^+] = \frac{\Delta\boldsymbol{Abs}(\boldsymbol{\lambda})}{\boldsymbol{\varepsilon}\{(\boldsymbol{E})\cdot\mathbf{H}^+),\boldsymbol{\lambda}\} - \boldsymbol{\varepsilon}\{(\boldsymbol{Z})\cdot\mathbf{H}^+),\boldsymbol{\lambda}\}}$$

To determine the quantum yield of ($E$)→($Z$) photoisomerization, first solutions of ($E$)·$\mathbf{H}^+$ were prepared by thermally equilibrating solutions of the respective ($Z$)-isomer at 5.7 mM in presence of 32 equiv. of TFA, similar as performed for the $^1$H NMR thermal isomerization experiments. The samples were subsequently diluted in $CHCl_3$ that was acidified with TFA, to reach a sample with a total of 470 equiv. of TFA. The sample was then cooled to 0° C or –10 °C and $Et_3N$ was added (1.5 equiv. with respect to TFA). Again, the concentrations were such that all incoming light was absorbed (Abs ≥ 2.0 at 455 nm). The *in situ* formed ($E$)-isomer was then irradiated with 455 nm light and also in this case, the change in absorbance was followed at a wavelength at which the absorbance was linear with concentration (Abs < 1.0). This change in absorbance was used to calculate the increase in the concentration of ($Z$)-isomer, by using the known molar absorptivity at the selected wavelength (see Table **S11** for an overview).

$$\Delta[(\boldsymbol{Z})] = \frac{\Delta\boldsymbol{Abs}(\boldsymbol{\lambda})}{\boldsymbol{\varepsilon}\{(\boldsymbol{Z}),\boldsymbol{\lambda}\} - \boldsymbol{\varepsilon}\{(\boldsymbol{E}),\boldsymbol{\lambda}\}}$$

**Table S10.** Overview of wavelengths (*λ*) used to follow (*Z*)·**H**$^+$→(*E*)·**H**$^+$ photoisomerization and the respective molar absorptivities (*ε*) of the (*E*)- and (*Z*)-isomers under the experimental conditions used.[a]

| **Compound** | **Concentration** | **ε (wavelength) M$^{-1}$ cm$^{-1}$** |
|---|---|---|
| (*Z*)-**1**·**H**$^+$ | 0.55 mM | 223.27 (620 nm) |
| (*E*)-**1**·**H**$^+$ | 0.55 mM | 2162.59 (620 nm) |
| (*Z*)-**2**·**H**$^+$ | 0.30 mM | 442.33 (620 nm) |
| (*E*)-**2**·**H**$^+$ | 0.30 mM | 5649.76 (620 nm) |
| (*Z*)-**3**·**H**$^+$ | 0.72 mM | 320.30 (620 nm) |
| (*E*)-**3**·**H**$^+$ | 0.72 mM | 2653.84 (620 nm) |
| (*Z*)-**4**·**H**$^+$ | 0.50 mM | 184.75 (660 nm) |
| (*E*)-**4**·**H**$^+$ | 0.50 mM | 2110.35 (660 nm) |
| (*Z*)-**5**·**H**$^+$ | 0.70 mM | 206.39 (600 nm) |
| (*E*)-**5**·**H**$^+$ | 0.70 mM | 1692.73 (600 nm) |

[a] The molar absorptivities were determined at the same concentration as used for the irradiation experiment to exclude differences due to solvatochromic effects of TFA and $Et_3N$ addition.

**Table S11.** Overview of wavelengths (*λ*) used to follow (*E*)→(*Z*) photoisomerization and the respective molar absorptivities (*ε*) of the (*E*)- and (*Z*)-isomers under the experimental conditions used.[a]

| **Compound** | **Concentration** | **ε (wavelength)** **$M^{-1}$ $cm^{-1}$** |
|---|---|---|
| (*E*)-**1** | 0.55 mM | 1144.04 (420 nm) |
| (Z)-**1** | 0.55 mM | 2027.41 (420 nm) |
| (*E*)-**2** | 0.30 mM | 3427.19 (565 nm) |
| (*Z*)-**2** | 0.30 mM | 308.69 (565 nm) |
| (*E*)-**3** | 0.72 mM | 1065.13 (420 nm) |
| (*Z*)-**3** | 0.72 mM | 2201.62 (420 nm) |
| (*E*)-**4** | - | - |
| (Z)-**4** | - | - |
| (*E*)-**5** | 0.70 mM | 1328.48 (420 nm) |
| (*Z*)-**5** | 0.70 mM | 2390.64 (420 nm) |

[a]The molar absorptivities were determined at the same concentration as used for the irradiation experiment to exclude differences due to solvatochromic effects of TFA and $Et_3N$ addition. The molar absorptivity of the pure (*Z*)-isomer was determined in the presence of TFA and $Et_3N$.

**Rates of formation of compound 1**

Note that the rate of formation of the photogenerated (*E*)-**1**·H$^+$ isomer was determined in duplicate in both degassed and aerated $CHCl_3$, which gave similar quantum yields.

*Degassed solution:*

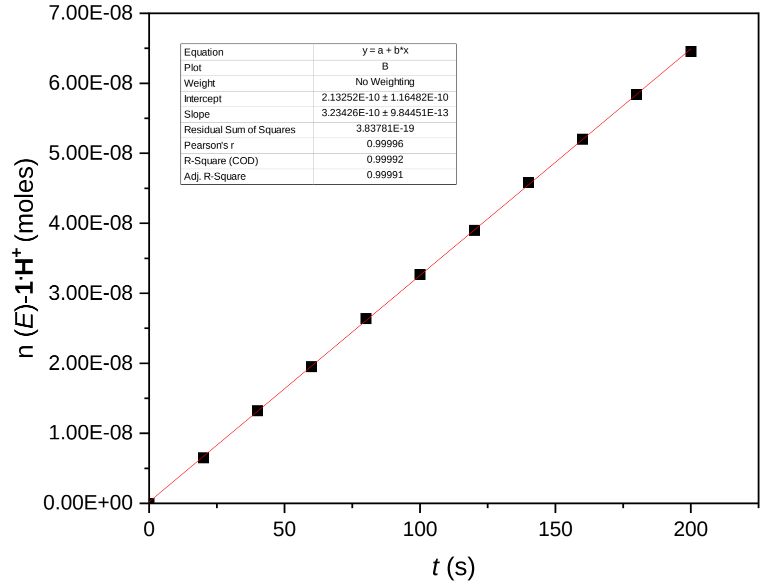


**Figure S87.** Molar amount of (*E*)-**1·H**$^+$ generated upon irradiation of a stirred (1200 rpm) solution of (Z)-**1** (0.55 mM, 2 mL degassed $CHCl_3$, 1 cm quartz cuvette, 273 K) with 455 nm light in presence of 470 equiv. TFA. Rate of formation: $3.23 \times 10^{-10}$ mol s$^{-1}$.

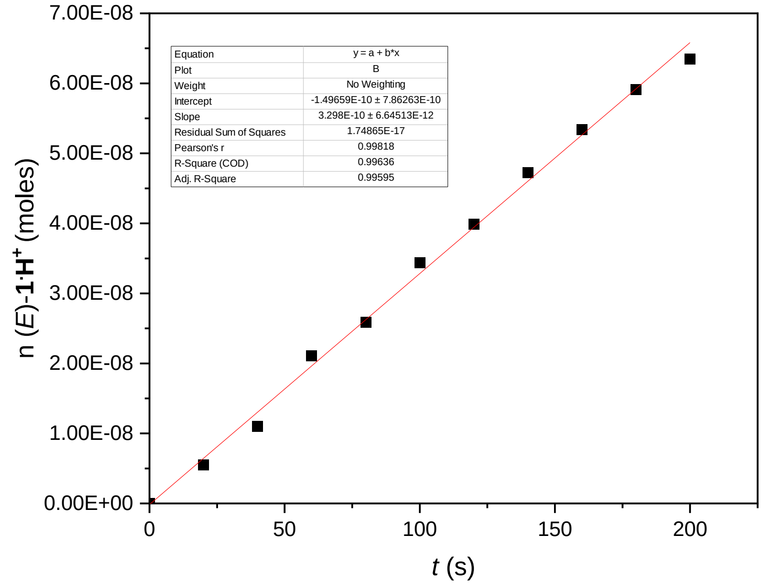


**Figure S88**. Molar amount of (*E*)-**1·H**$^+$ generated upon irradiation of a stirred (1200 rpm) solution of (Z)-**1** (0.55 mM, 2 mL degassed $CHCl_3$, 1 cm quartz cuvette, 273 K) with 455 nm light in presence of 470 equiv. TFA. Rate of formation: $3.30 \times 10^{-10}$ mol s$^{-1}$.

*Aerated solution:*

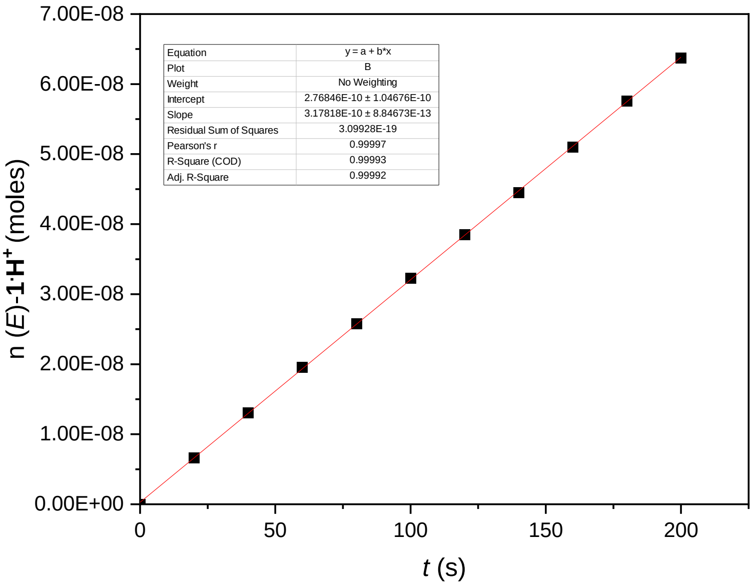


**Figure S89.** Molar amount of (*E*)-**1**·**H**$^+$ generated upon irradiation of a stirred (1200 rpm) solution of (Z)-**1** (0.55 mM, 2 mL aerated $CHCl_3$, 1 cm quartz cuvette, 273 K) with 455 nm light in presence of 470 equiv. TFA. Rate of formation: $3.18 \times 10^{-10}$ mol $s^{-1}$.

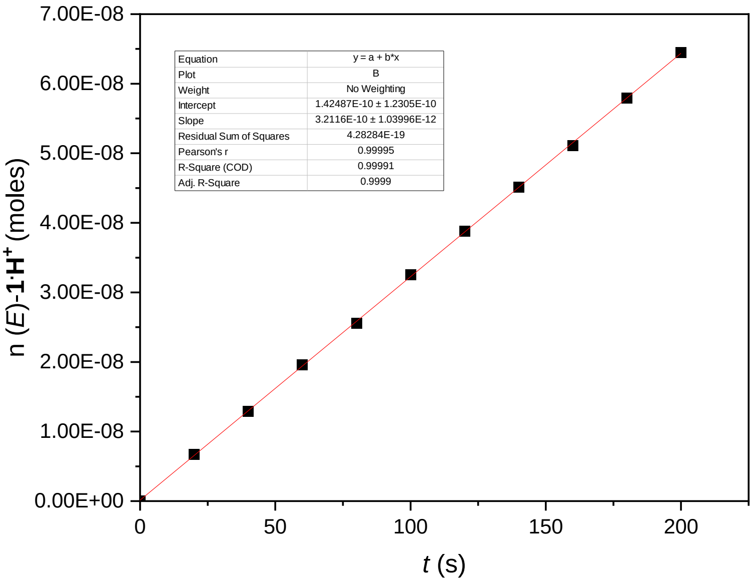


**Figure S90.** Molar amount of (*E*)-**1**·**H**$^+$ generated upon irradiation of a stirred (1200 rpm) solution of (Z)-**1** (0.55 mM, 2 mL aerated $CHCl_3$, 1 cm quartz cuvette, 273 K) with 455 nm light in presence of 470 equiv. TFA. Rate of formation: $3.21 \times 10^{-10}$ mol $s^{-1}$.

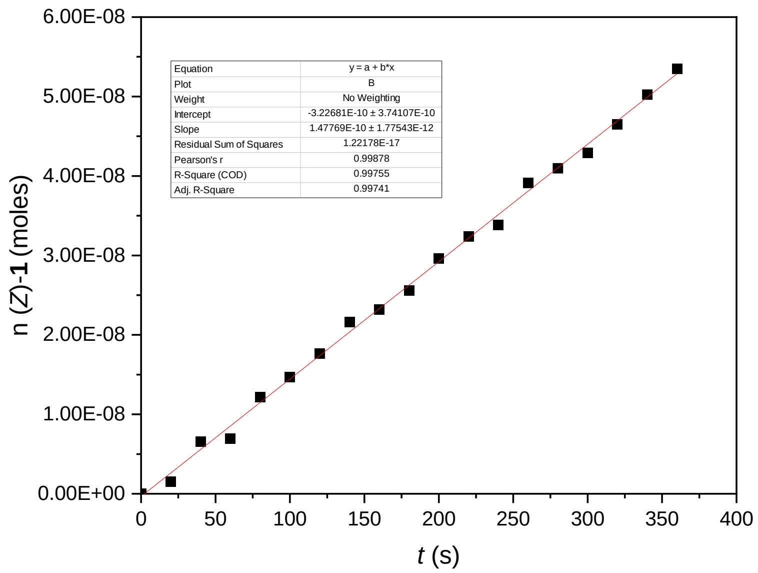


**Figure S91.** Molar amount of (*Z*)-**1** generated upon irradiation of a stirred (1200 rpm) solution of (*E*)-**1** (0.55 mM, 2 mL degassed $CHCl_3$, 1 cm quartz cuvette, 273 K) with 455 nm light in presence of 470 equiv. TFA and 700 equiv. of $Et_3N$. Rate of formation: $1.48 \times 10^{-10}$ mol $s^{-1}$.

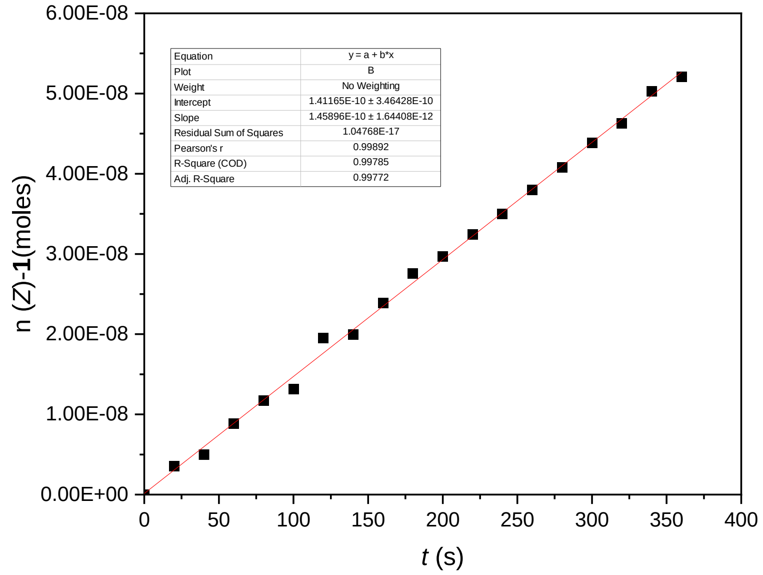


**Figure S92.** Molar amount of (*Z*)-**1** generated upon irradiation of a stirred (1200 rpm) solution of (*E*)-**1** (0.55 mM, 2 mL degassed $CHCl_3$, 1 cm quartz cuvette, 273 K) with 455 nm light in presence of 470 equiv. TFA and 700 equiv. of $Et_3N$. Rate of formation: $1.46 \times 10^{-10}$ mol $s^{-1}$.

## Rates of formation of compound 2

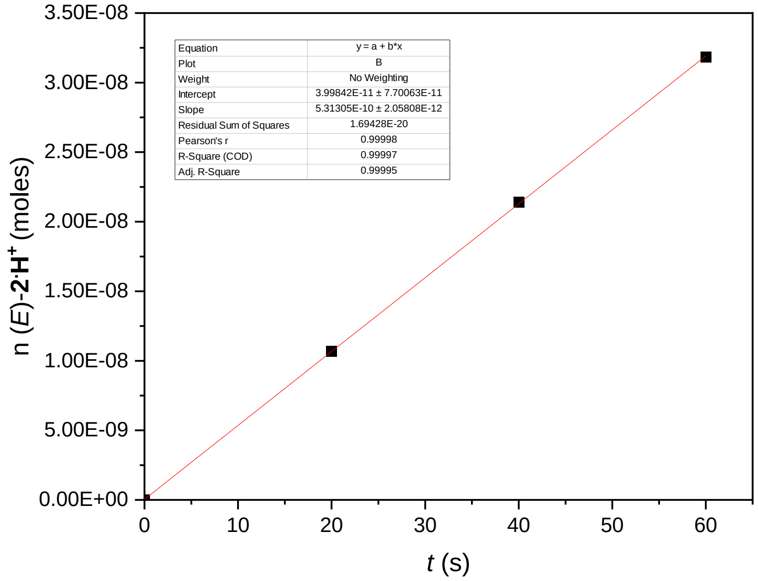


**Figure S93** Molar amount of (*E*)-**2**·**H**$^{+}$ generated upon irradiation of a stirred (1200 rpm) solution of (Z)-**2** (0.30 mM, 2 mL degassed $CHCl_3$, 1 cm quartz cuvette, 273 K) with 455 nm light in presence of 470 equiv. TFA. Rate of formation: $5.31 \times 10^{-10}$ mol $s^{-1}$.

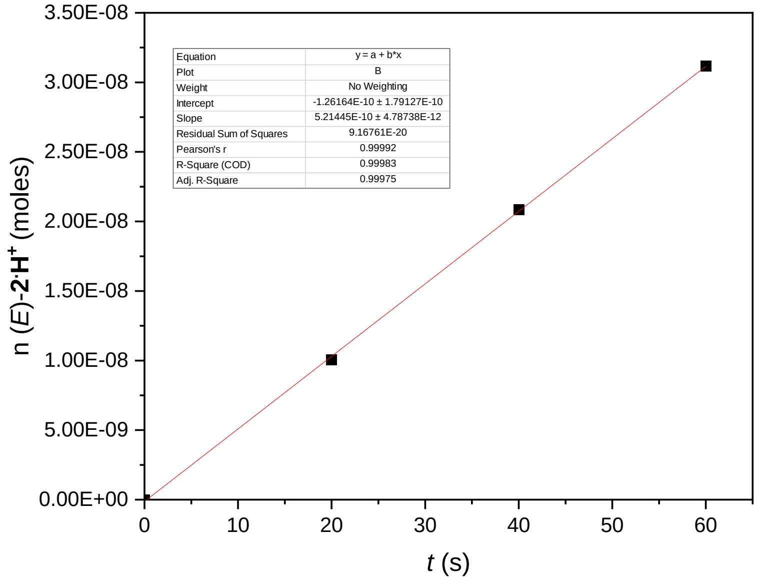


**Figure S94.** Molar amount of (*E*)-**2**·**H**$^{+}$ generated upon irradiation of a stirred (1200 rpm) solution of (Z)-**2** (0.30 mM, 2 mL degassed $CHCl_3$, 1 cm quartz cuvette, 273 K) with 455 nm light in presence of 470 equiv. TFA. Rate of formation: $5.21 \times 10^{-10}$ mol $s^{-1}$.

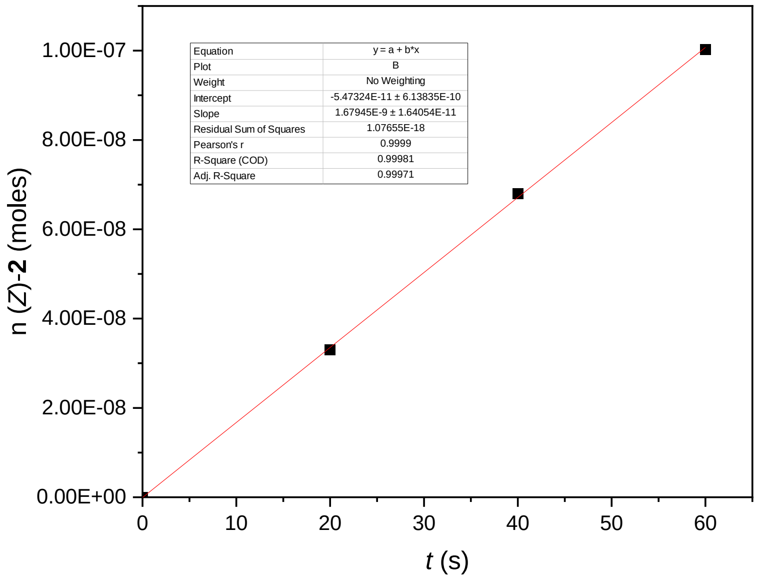


**Figure S95.** Molar amount of (*Z*)-**2** generated upon irradiation of a stirred (1200 rpm) solution of (*E*)-**2** (0.30 mM, 2 mL degassed $CHCl_3$, 1 cm quartz cuvette, 263 K) with 455 nm light in presence of 470 equiv. TFA and 700 equiv. of $Et_3N$. Rate of formation: $1.68 \times 10^{-9}$ mol $s^{-1}$.

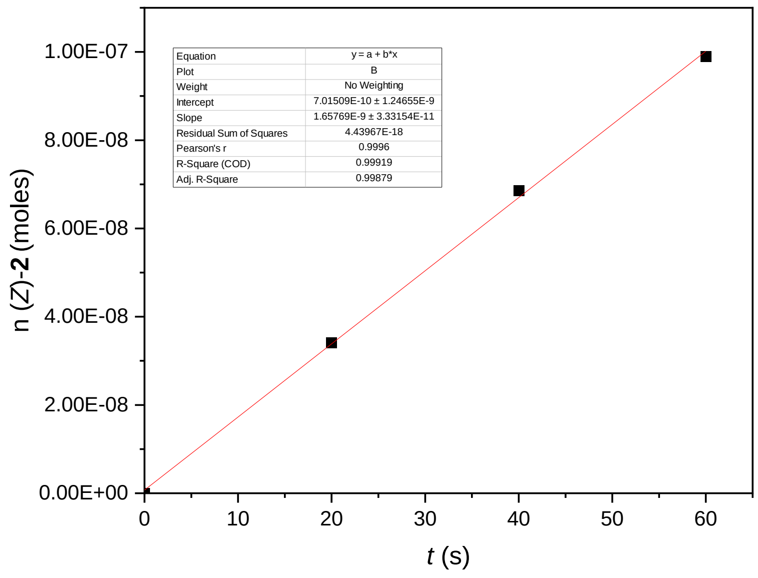


**Figure S96.** Molar amount of (*Z*)-**2** generated upon irradiation of a stirred (1200 rpm) solution of (*E*)-**2** (0.30 mM, 2 mL degassed $CHCl_3$, 1 cm quartz cuvette, 263 K) with 455 nm light in presence of 470 equiv. TFA and 700 equiv. of $Et_3N$. Rate of formation: $1.66 \times 10^{-9}$ mol $s^{-1}$.

## Rates of formation of compound 3

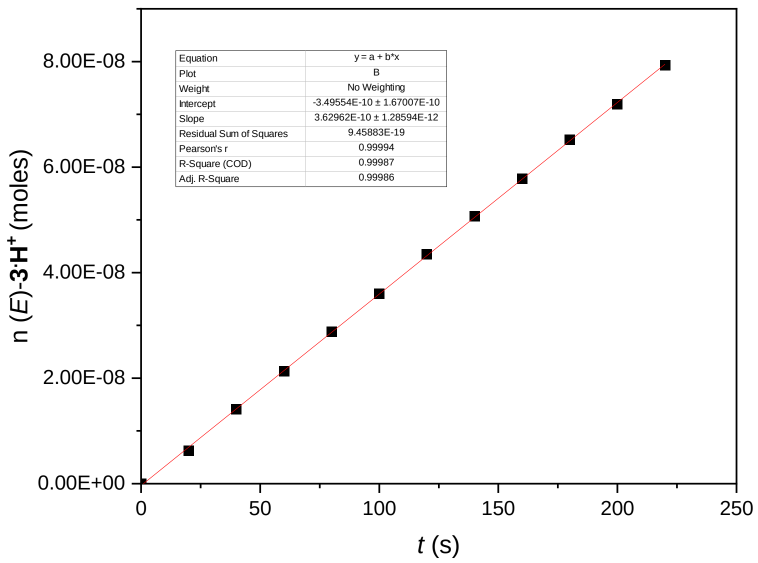


**Figure S97** Molar amount of (*E*)-**3·H**$^+$ generated upon irradiation of a stirred (1200 rpm) solution of (Z)-**3** (0.72 mM, 2 mL degassed $CHCl_3$, 1 cm quartz cuvette, 273 K) with 455 nm light in presence of 470 equiv. TFA. Rate of formation: $3.63 \times 10^{-10}$ mol $s^{-1}$.

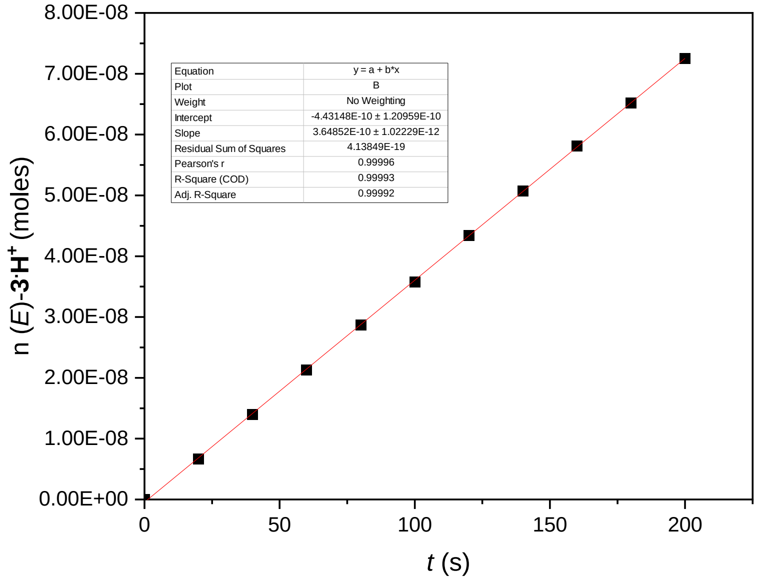


**Figure S98** Molar amount of (*E*)-**3·H**$^+$ generated upon irradiation of a stirred (1200 rpm) solution of (Z)-**3** (0.72 mM, 2 mL degassed $CHCl_3$, 1 cm quartz cuvette, 273 K) with 455 nm light in presence of 470 equiv. TFA. Rate of formation: $3.65 \times 10^{-10}$ mol $s^{-1}$.

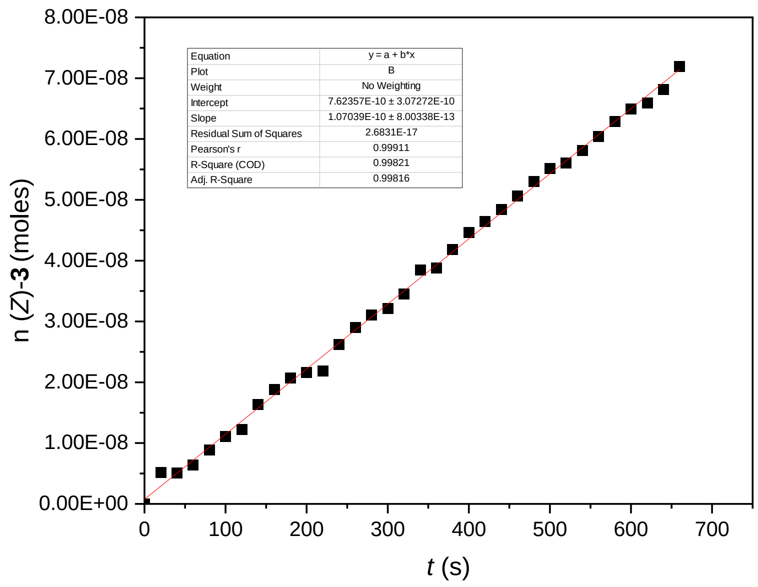


**Figure S99.** Molar amount of (*Z*)-**3** generated upon irradiation of a stirred (1200 rpm) solution of (*E*)-**3** (0.72 mM, 2 mL degassed $CHCl_3$, 1 cm quartz cuvette, 273 K) with 455 nm light in presence of 470 equiv. TFA and 700 equiv. of $Et_3N$. Rate of formation: $1.07 \times 10^{-10}$ mol $s^{-1}$.

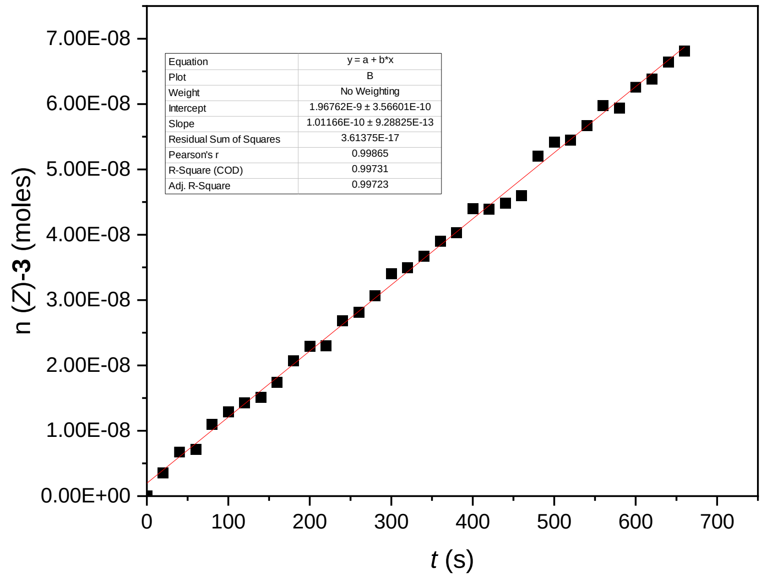


**Figure S100.** Molar amount of (*Z*)-**3** generated upon irradiation of a stirred (1200 rpm) solution of (*E*)-**3** (0.72 mM, 2 mL degassed $CHCl_3$, 1 cm quartz cuvette, 273 K) with 455 nm light in presence of 470 equiv. TFA and 700 equiv. of $Et_3N$. Rate of formation: $1.01 \times 10^{-10}$ mol $s^{-1}$.

## Rates of formation of compound 4

Note that $E \rightarrow Z$ isomerization was not followed due to rapid competing thermal isomerization.

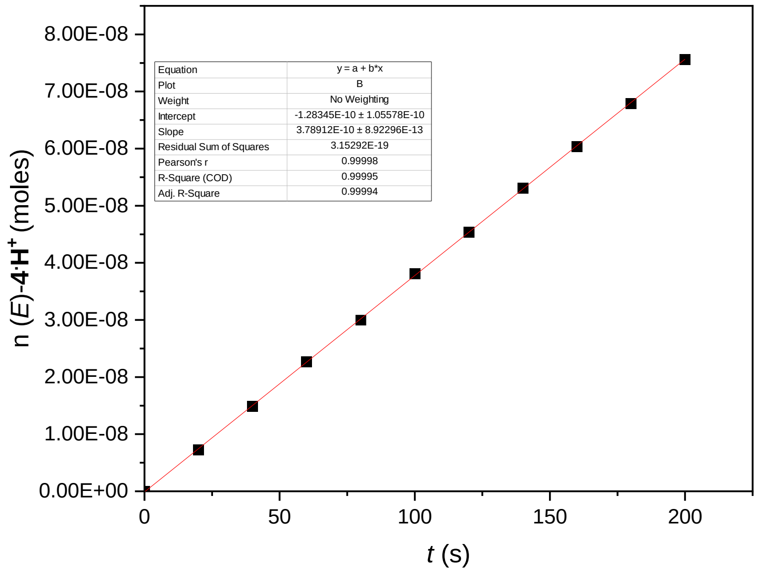


**Figure S101**. Molar amount of (*E*)-**4**·**H**$^+$ generated upon irradiation of a stirred (1200 rpm) solution of (Z)-**4** (0.50 mM, 2 mL degassed $CHCl_3$, 1 cm quartz cuvette, 273 K) with 455 nm light in presence of 470 equiv. TFA. Rate of formation: $3.79 \times 10^{-10}$ mol $s^{-1}$.

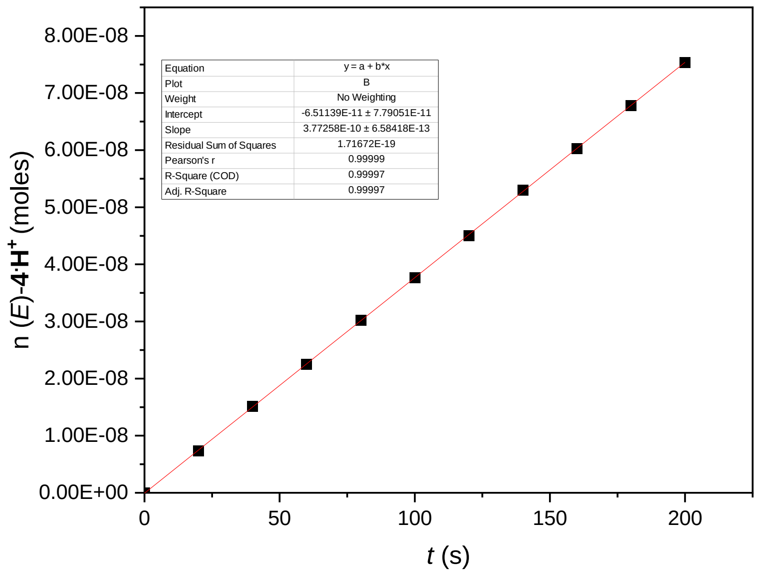


**Figure S102.** Molar amount of (*E*)-**4**·**H**$^+$ generated upon irradiation of a stirred (1200 rpm) solution of (Z)-**4** (0.50 mM, 2 mL degassed $CHCl_3$, 1 cm quartz cuvette, 273 K) with 455 nm light in presence of 470 equiv. TFA. Rate of formation: $3.77 \times 10^{-10}$ mol $s^{-1}$.

## Rates of formation of compound 5

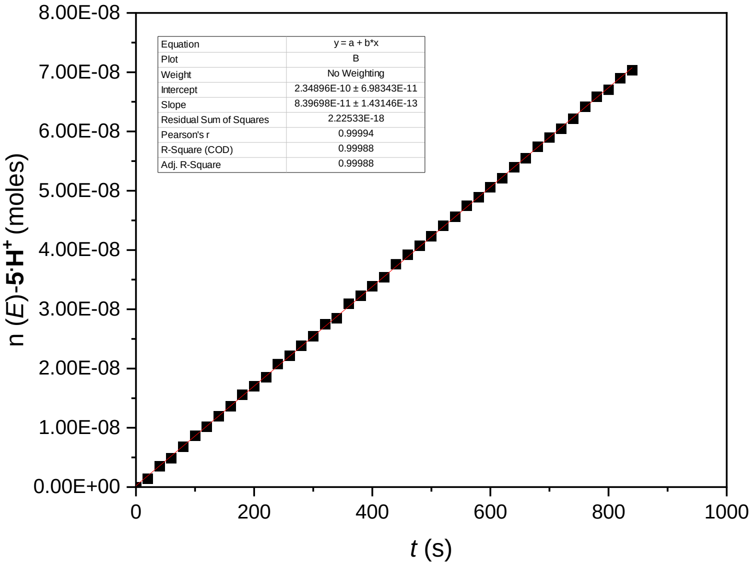


**Figure S103.** Molar amount of (*E*)-**5·H**$^+$ generated upon irradiation of a stirred (1200 rpm) solution of (Z)-**5** (0.70 mM, 2 mL degassed $CHCl_3$, 1 cm quartz cuvette, 273 K) with 455 nm light in presence of 470 equiv. TFA. Rate of formation: $8.40 \times 10^{-11}$ mol $s^{-1}$.

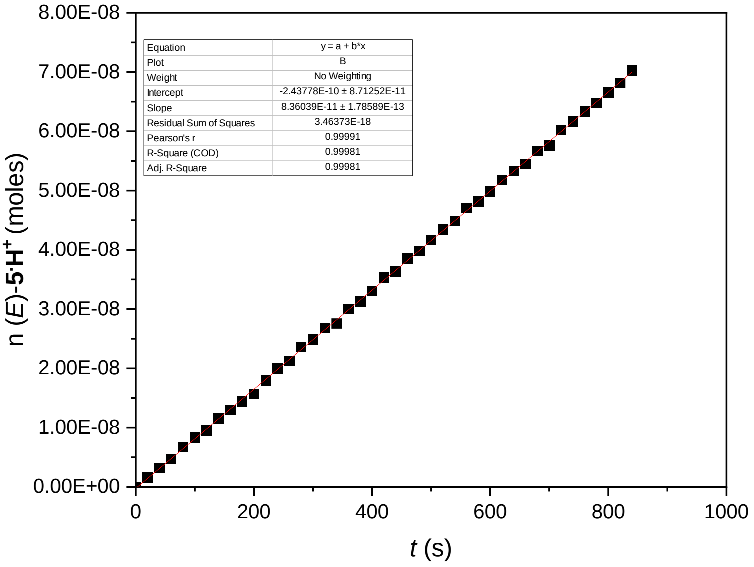


**Figure S104.** Molar amount of (*E*)-**5·H**$^+$ generated upon irradiation of a stirred (1200 rpm) solution of (Z)-**5** (0.70 mM, 2 mL degassed $CHCl_3$, 1 cm quartz cuvette, 273 K) with 455 nm light in presence of 470 equiv. TFA. Rate of formation: $8.36 \times 10^{-11}$ mol $s^{-1}$.

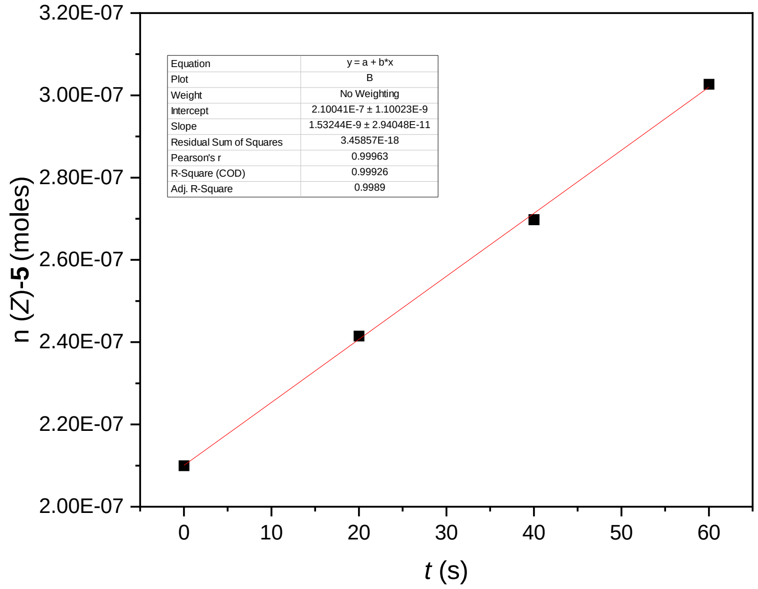


**Figure S105.** Molar amount of (*Z*)-**5** generated upon irradiation of a stirred (1200 rpm) solution of (*E*)-**5** (0.70 mM, 2 mL degassed $CHCl_3$, 1 cm quartz cuvette, 263 K) with 455 nm light in presence of 470 equiv. TFA and 700 equiv. of $Et_3N$. Rate of formation: $1.53 \times 10^{-9}$ mol $s^{-1}$.

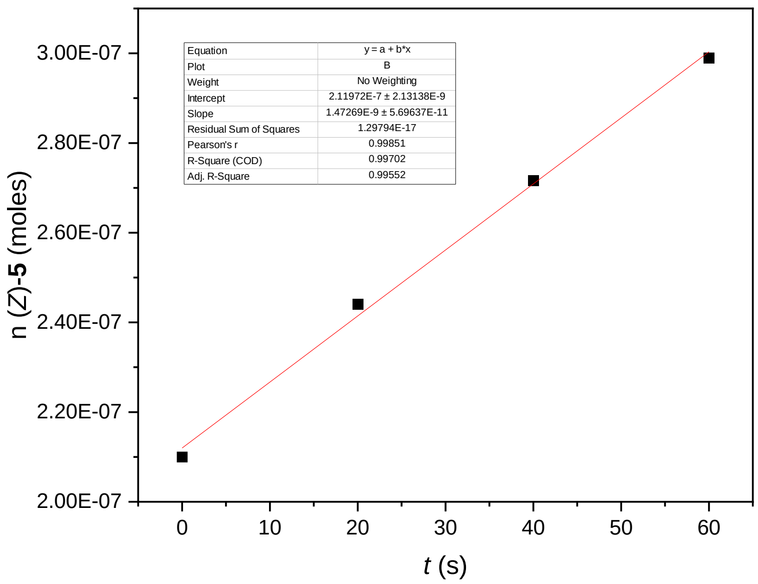


**Figure S106.** Molar amount of (*Z*)-**5** generated upon irradiation of a stirred (1200 rpm) solution of (*E*)-**5** (0.70 mM, 2 mL degassed $CHCl_3$, 1 cm quartz cuvette, 263 K) with 455 nm light in presence of 470 equiv. TFA and 700 equiv. of $Et_3N$. Rate of formation: $1.47 \times 10^{-9}$ mol $s^{-1}$.

# 11. Computational details

All calculations were performed using Gaussian 09,[11] and data was visualized using Avogadro 1.2.0[12] and Gaussview 6.[13] Geometry optimizations were performed at the B3LYP 6-311++G(d,p) level of theory using an IEFPCM solvation model ($CHCl_3$). All computed stationary points were subjected to frequency analysis and confirmed as local minima through analytical computation of force constants (no imaginary frequencies found for all minima). UV-Vis spectra were calculated by TD-DFT on the B3LYP 6-311++G(d,p) level of theory with non-equilibrium IEFPCM ($CHCl_3$), solving for the first 30 singlet excited states for the most stable conformers of the configurational isomers of **1**.This level of theory has been previously used to describe the related hemi-thioindigo[14] scaffold, and a similar approach proved accurate for hemi-indigo (PCM-TD-B3LYP/6-311+G(2d,p)//PCM-B3LYP/6- 311(d,p)).[15]

### Overview of relative energies

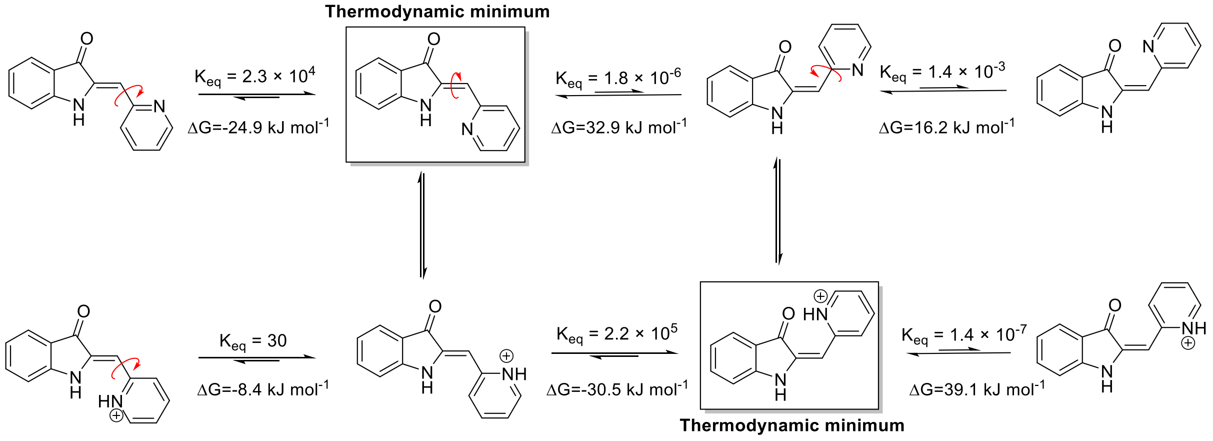


**Scheme S2.** Overview of relative energies of **1**. Calculated at B3LYP 6-311++G(d,p) level of theory with an IEFPCM $CHCl_3$ solvent model.

**(*Z*)-1 A**

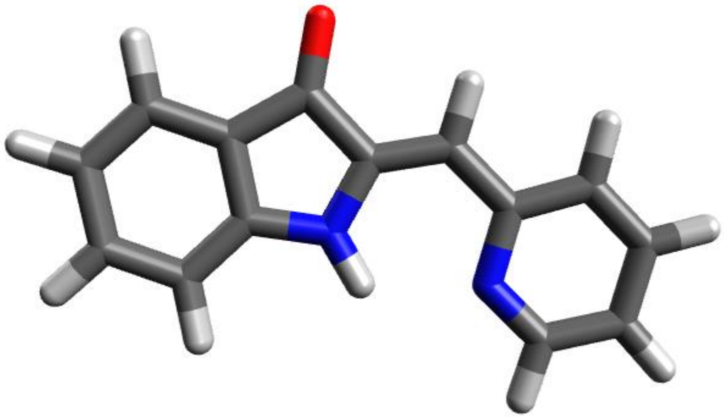

**Figure S107.** The optimized geometry of (*Z*)-**1** A calculated at B3LYP 6-311++G(d,p) level of theory with an IEFPCM $CHCl_3$ solvent model. Color codes: O = red; N = blue.

Zero-point correction= 0.207407 (Hartree/Particle)
Thermal correction to Energy= 0.220067
Thermal correction to Enthalpy= 0.221011
Thermal correction to Gibbs Free Energy= 0.167477
Sum of electronic and zero-point Energies= -724.234021
Sum of electronic and thermal Energies= -724.221361
Sum of electronic and thermal Enthalpies= -724.220417
Sum of electronic and thermal Free Energies= -724.273950

-----------------------------------------------------------------------------------------------

| Atom | X | Y | Z |
| --- | --- | --- | --- |
| O | -1.0329710000 | 2.9053390000 | 0.0000000000 |
| N | 2.1683210000 | -1.2319300000 | 0.0000000000 |
| N | -0.5617390000 | -0.5679510000 | 0.0000000000 |
| C | 3.7502700000 | 0.5856610000 | 0.0000000000 |
| C | 4.8080910000 | -0.3119190000 | 0.0000000000 |
| C | 4.5326010000 | -1.6786690000 | 0.0000000000 |
| C | 3.1981710000 | -2.0804890000 | 0.0000000000 |
| C | 2.4298300000 | 0.0980500000 | 0.0000000000 |
| C | 1.3118200000 | 1.0251400000 | 0.0000000000 |
| C | -1.1409400000 | 1.6876880000 | 0.0000000000 |
| C | 0.0000000000 | 0.6922290000 | 0.0000000000 |
| C | -3.7057100000 | 1.1968670000 | 0.0000000000 |
| C | -2.3533700000 | 0.8579480000 | 0.0000000000 |
| C | -1.9460090000 | -0.4922220000 | 0.0000000000 |
| C | -2.8844790000 | -1.5232020000 | 0.0000000000 |
| C | -4.2328090000 | -1.1667530000 | 0.0000000000 |
| C | -4.6497700000 | 0.1743170000 | 0.0000000000 |
| H | 3.9269300000 | 1.6546310000 | 0.0000000000 |
| H | 5.8311400000 | 0.0462320000 | 0.0000000000 |
| H | 5.3252920000 | -2.4163080000 | 0.0000000000 |
| H | 2.9458120000 | -3.1370590000 | 0.0000000000 |
| H | 1.5349190000 | 2.0858000000 | 0.0000000000 |
| H | -4.0053010000 | 2.2387670000 | 0.0000000000 |
| H | -2.5806380000 | -2.5632420000 | 0.0000000000 |
| H | -4.9808690000 | -1.9518930000 | 0.0000000000 |
| H | -5.7075100000 | 0.4073660000 | 0.0000000000 |
| H | 0.0217510000 | -1.3957110000 | 0.0000000000 |

-----------------------------------------------------------------------------------------------

**($Z$)-1 B**

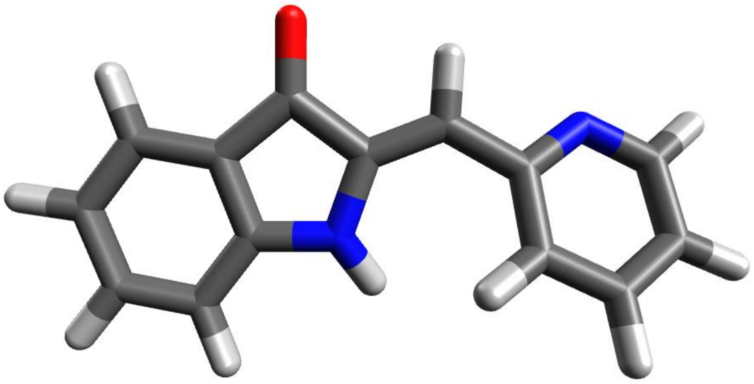

**Figure S108.** The optimized geometry of ($Z$)-**1** B calculated at B3LYP 6-311++G(d,p) level of theory with an IEFPCM $CHCl_3$ solvent model. Color codes: O = red; N = blue.

Zero-point correction= 0.207359 (Hartree/Particle)

Thermal correction to Energy= 0.220185

Thermal correction to Enthalpy= 0.221129

Thermal correction to Gibbs Free Energy= 0.166837

Sum of electronic and zero-point Energies= -724.223958

Sum of electronic and thermal Energies= -724.211133

Sum of electronic and thermal Enthalpies= -724.210188

Sum of electronic and thermal Free Energies= -724.264481

| Atom | X | Y | Z |
|---|---|---|---|
| O | -1.3074680000 | 2.6778090000 | 0.2334210000 |
| N | 3.4831030000 | 1.0695080000 | -0.3766240000 |
| N | -0.5759330000 | -0.7535670000 | 0.0417490000 |
| C | 4.6822210000 | 0.4920400000 | -0.4356590000 |
| C | 4.9209990000 | -0.8372700000 | -0.0851200000 |
| C | 3.8456260000 | -1.5966860000 | 0.3658340000 |
| C | 2.5857900000 | -1.0131340000 | 0.4264420000 |
| C | 2.4282410000 | 0.3280020000 | 0.0325930000 |
| C | 1.1550210000 | 1.0401190000 | 0.0637670000 |
| C | -1.3217650000 | 1.4605680000 | 0.1342400000 |
| C | -0.1056700000 | 0.5591610000 | 0.0820780000 |
| C | -3.8433570000 | 0.7932250000 | 0.0254950000 |
| C | -2.4697570000 | 0.5495020000 | 0.0471160000 |
| C | -1.9684680000 | -0.7605180000 | -0.0441670000 |
| C | -2.8256960000 | -1.8506430000 | -0.1786580000 |
| C | -4.1954090000 | -1.5916460000 | -0.2054670000 |
| C | -4.7085800000 | -0.2884260000 | -0.0999770000 |
| H | 5.4991320000 | 1.1231520000 | -0.7743160000 |
| H | 5.9194430000 | -1.2513420000 | -0.1513130000 |
| H | 3.9853230000 | -2.6262900000 | 0.6746510000 |
| H | 1.7498690000 | -1.5774110000 | 0.8180740000 |
| H | 1.2319870000 | 2.1215960000 | 0.0358960000 |
| H | -4.2169710000 | 1.8080800000 | 0.0994140000 |
| H | -2.4464330000 | -2.8626430000 | -0.2563850000 |
| H | -4.8832820000 | -2.4235860000 | -0.3080480000 |
| H | -5.7800330000 | -0.1312480000 | -0.1203630000 |
| H | -0.0246580000 | -1.5201300000 | -0.3119670000 |

**(Z)-1·H⁺ A**

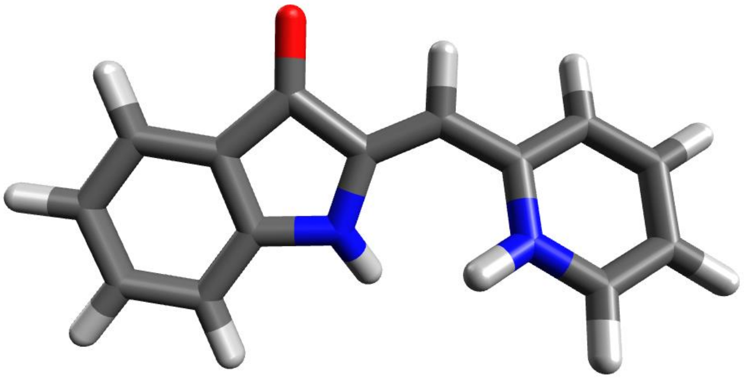

**Figure S109.** The optimized geometry of (*Z*)-**1·H**$^+$ A calculated at B3LYP 6-311++G(d,p) level of theory with an IEFPCM $CHCl_3$ solvent model. Color codes: O = red; N = blue.

Zero-point correction= 0.221632 (Hartree/Particle)

Thermal correction to Energy= 0.234526

Thermal correction to Enthalpy= 0.235470

Thermal correction to Gibbs Free Energy= 0.181290

Sum of electronic and zero-point Energies= -724.640101

Sum of electronic and thermal Energies= -724.627207

Sum of electronic and thermal Enthalpies= -724.626263

Sum of electronic and thermal Free Energies= -724.680443

| Atom | X | Y | Z |
|---|---|---|---|
| O | -1.3184500000 | 2.6790080000 | 0.2497350000 |
| N | 2.4933840000 | -0.8971500000 | 0.3613000000 |
| N | -0.5716370000 | -0.7239770000 | -0.1127220000 |
| C | 1.1321890000 | 1.0902810000 | -0.0404840000 |
| C | 2.3977980000 | 0.4084090000 | -0.0284200000 |
| C | 3.6564050000 | -1.5876160000 | 0.4159320000 |
| C | 4.8344090000 | -0.9774770000 | 0.0582140000 |
| C | 4.7978650000 | 0.3684410000 | -0.3333710000 |
| C | 3.5959840000 | 1.0533360000 | -0.3699380000 |
| C | -1.3542200000 | 1.4736400000 | 0.1137820000 |
| C | -0.1213640000 | 0.5761460000 | -0.0315950000 |
| C | -3.8671930000 | 0.7787150000 | 0.1262900000 |
| C | -2.4923210000 | 0.5521390000 | 0.0614720000 |
| C | -1.9874410000 | -0.7438260000 | -0.1063760000 |
| C | -2.8246710000 | -1.8417240000 | -0.2256480000 |
| C | -4.2007700000 | -1.6031100000 | -0.1641920000 |
| C | -4.7216410000 | -0.3141550000 | 0.0137920000 |
| H | 1.1822590000 | 2.1729630000 | -0.0481540000 |
| H | 3.5872060000 | -2.6113180000 | 0.7551740000 |
| H | 5.7599950000 | -1.5338700000 | 0.0945670000 |
| H | 5.7139930000 | 0.8762030000 | -0.6084610000 |
| H | 3.5550570000 | 2.0915800000 | -0.6697640000 |
| H | -4.2509090000 | 1.7836770000 | 0.2551520000 |
| H | -2.4349160000 | -2.8436640000 | -0.3574520000 |
| H | -4.8814760000 | -2.4416940000 | -0.2537700000 |
| H | -5.7940180000 | -0.1722700000 | 0.0608100000 |
| H | -0.0721130000 | -1.4459040000 | -0.6142850000 |
| H | 1.6601110000 | -1.3590750000 | 0.7115240000 |

**(*Z*)-1·H$^+$ B**

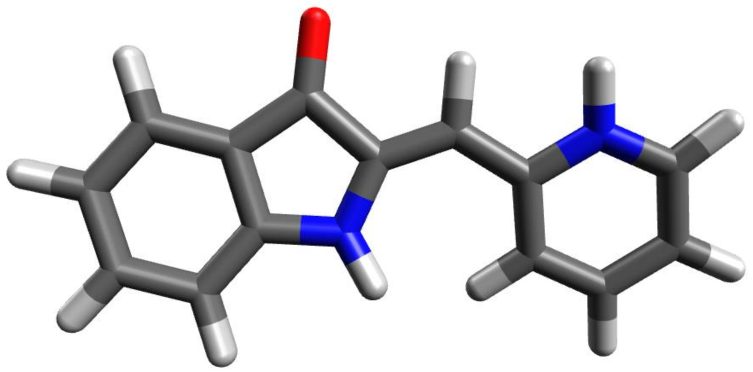

**Figure S110.** The optimized geometry of (*Z*)-**1·H$^+$** B calculated at B3LYP 6-311++G(d,p) level of theory with an IEFPCM $CHCl_3$ solvent model. Color codes: O = red; N = blue.

Zero-point correction= 0.221670 (Hartree/Particle)

Thermal correction to Energy= 0.234616

Thermal correction to Enthalpy= 0.235560

Thermal correction to Gibbs Free Energy= 0.181217

Sum of electronic and zero-point Energies= -724.643205

Sum of electronic and thermal Energies= -724.630259

Sum of electronic and thermal Enthalpies= -724.629315

Sum of electronic and thermal Free Energies= -724.683658

| Atom | X | Y | Z |
|---|---|---|---|
| O | 1.2439130000 | 2.6358420000 | 0.1405610000 |
| N | -3.4978950000 | 1.0082410000 | -0.1799310000 |
| N | 0.6650610000 | -0.7951930000 | -0.0238140000 |
| C | -1.1269440000 | 0.9443540000 | 0.0250900000 |
| C | -2.3774050000 | 0.2469520000 | 0.0185030000 |
| C | -2.5960680000 | -1.1269440000 | 0.2207140000 |
| C | -3.8781890000 | -1.6472440000 | 0.1983240000 |
| C | -4.9840880000 | -0.8159120000 | -0.0254560000 |
| C | -4.7591240000 | 0.5278620000 | -0.2070800000 |
| C | 1.3394470000 | 1.4281910000 | 0.0758520000 |
| C | 0.1438610000 | 0.4618670000 | 0.0247210000 |
| C | 3.8840060000 | 0.8573240000 | 0.0376400000 |
| C | 2.5215600000 | 0.5639290000 | 0.0297810000 |
| C | 2.0741610000 | -0.7629620000 | -0.0424630000 |
| C | 2.9588910000 | -1.8272910000 | -0.1112790000 |
| C | 4.3237960000 | -1.5206430000 | -0.1034830000 |
| C | 4.7872490000 | -0.2008640000 | -0.0289280000 |
| H | -1.1724630000 | 2.0292140000 | 0.0261760000 |
| H | -1.7644880000 | -1.7801830000 | 0.4336890000 |
| H | -4.0253640000 | -2.7072660000 | 0.3642540000 |
| H | -5.9925170000 | -1.2029740000 | -0.0473910000 |
| H | -5.5426470000 | 1.2533200000 | -0.3748520000 |
| H | 4.2230820000 | 1.8848570000 | 0.0926870000 |
| H | 2.6161980000 | -2.8534140000 | -0.1668500000 |
| H | 5.0411890000 | -2.3314180000 | -0.1551740000 |
| H | 5.8524070000 | -0.0068030000 | -0.0239060000 |
| H | 0.1465370000 | -1.6406350000 | -0.1991900000 |
| H | -3.3703190000 | 2.0055110000 | -0.3193430000 |

**(*E*)-1 A**

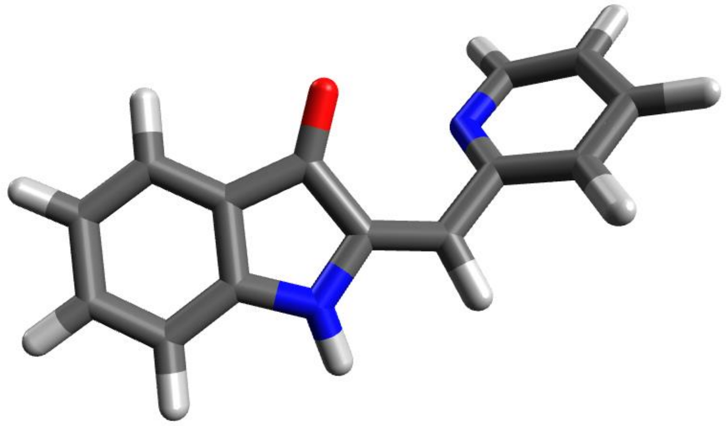

**Figure S111.** The optimized geometry of (*E*)-**1** A calculated at B3LYP 6-311++G(d,p) level of theory with an IEFPCM $CHCl_3$ solvent model. Color codes:O = red; N = blue.

| | |
|---|---|
| Zero-point correction= | 0.206512 (Hartree/Particle) |
| Thermal correction to Energy= | 0.219542 |
| Thermal correction to Enthalpy= | 0.220486 |
| Thermal correction to Gibbs Free Energy= | 0.165614 |
| Sum of electronic and zero-point Energies= | -724.214374 |
| Sum of electronic and thermal Energies= | -724.201344 |
| Sum of electronic and thermal Enthalpies= | -724.200400 |
| Sum of electronic and thermal Free Energies= | -724.255271 |

| Atom | X | Y | Z |
|---|---|---|---|
| O | 0.0251410000 | -1.5791100000 | -0.6071230000 |
| N | 2.4558180000 | -0.5679170000 | 0.8091870000 |
| N | -1.3051670000 | 1.5829520000 | 0.1439940000 |
| C | 3.4723990000 | 1.0814490000 | -0.6155170000 |
| C | 4.6813140000 | 0.3971150000 | -0.5830950000 |
| C | 4.7634200000 | -0.7811980000 | 0.1535510000 |
| C | 3.6240120000 | -1.2105640000 | 0.8345530000 |
| C | 2.3678230000 | 0.5574710000 | 0.0766400000 |
| C | 1.0968090000 | 1.2932250000 | 0.0566890000 |
| C | -0.6474640000 | -0.6201820000 | -0.2712590000 |
| C | -0.1560750000 | 0.7929690000 | 0.0201080000 |
| C | -3.0979010000 | -1.5126320000 | -0.2773220000 |
| C | -2.1110730000 | -0.5342280000 | -0.1580380000 |
| C | -2.4508600000 | 0.8037030000 | 0.1092420000 |
| C | -3.7813410000 | 1.1871730000 | 0.2804150000 |
| C | -4.7549530000 | 0.1962300000 | 0.1683790000 |
| C | -4.4281150000 | -1.1415320000 | -0.1104090000 |
| H | 3.3722950000 | 2.0036230000 | -1.1761760000 |
| H | 5.5424360000 | 0.7778460000 | -1.1203900000 |
| H | 5.6838650000 | -1.3490270000 | 0.2132520000 |
| H | 3.6574080000 | -2.1133150000 | 1.4384450000 |
| H | 1.1912630000 | 2.3768950000 | 0.0845560000 |
| H | -2.8216790000 | -2.5395520000 | -0.4878610000 |
| H | -4.0513160000 | 2.2156450000 | 0.4898360000 |
| H | -5.7964060000 | 0.4692390000 | 0.2983090000 |
| H | -5.2160490000 | -1.8801840000 | -0.1926420000 |
| H | -1.2854810000 | 2.5124710000 | 0.5337670000 |

**(*E*)-1 B**

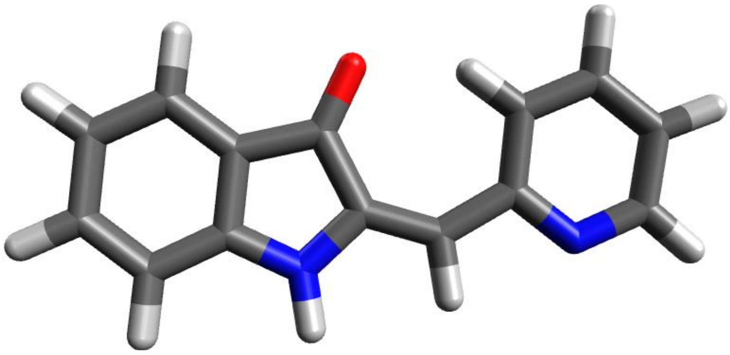

**Figure S112.** The optimized geometry of (*E*)-**1** B calculated at B3LYP 6-311++G(d,p) level of theory with an IEFPCM $CHCl_3$ solvent model. Color codes: O = red; N = blue.

Zero-point correction= 0.207016 (Hartree/Particle)

Thermal correction to Energy= 0.219999

Thermal correction to Enthalpy= 0.220943

Thermal correction to Gibbs Free Energy= 0.166244

Sum of electronic and zero-point Energies= -724.220666

Sum of electronic and thermal Energies= -724.207683

Sum of electronic and thermal Enthalpies= -724.206739

Sum of electronic and thermal Free Energies= -724.261438

| Atom | X | Y | Z |
|---|---|---|---|
| O | 0.1914070000 | 1.8468370000 | -0.0000240000 |
| N | -3.4395860000 | -1.3330240000 | 0.0000200000 |
| N | 1.2523560000 | -1.5043050000 | -0.0000370000 |
| C | -4.6911790000 | -0.8805900000 | 0.0000330000 |
| C | -5.0242270000 | 0.4745590000 | 0.0000230000 |
| C | -3.9849610000 | 1.3997080000 | -0.0000020000 |
| C | -2.6695150000 | 0.9502570000 | -0.0000150000 |
| C | -2.4214730000 | -0.4342430000 | -0.0000030000 |
| C | -1.1100680000 | -1.0736290000 | -0.0000160000 |
| C | 0.7815570000 | 0.7727050000 | -0.0000210000 |
| C | 0.1687980000 | -0.6169110000 | -0.0000250000 |
| C | 3.2929100000 | 1.4544880000 | 0.0000120000 |
| C | 2.2281360000 | 0.5513350000 | -0.0000050000 |
| C | 2.4585880000 | -0.8370230000 | -0.0000100000 |
| C | 3.7572280000 | -1.3500170000 | 0.0000070000 |
| C | 4.8074440000 | -0.4357050000 | 0.0000260000 |
| C | 4.5892570000 | 0.9539280000 | 0.0000270000 |
| H | -5.4719820000 | -1.6362900000 | 0.0000510000 |
| H | -6.0620700000 | 0.7845210000 | 0.0000330000 |
| H | -4.1925010000 | 2.4638720000 | -0.0000110000 |
| H | -1.8399090000 | 1.6414240000 | -0.0000340000 |
| H | -1.2103950000 | -2.1565010000 | -0.0000160000 |
| H | 3.1005740000 | 2.5212880000 | 0.0000140000 |
| H | 3.9433130000 | -2.4171960000 | 0.0000040000 |
| H | 5.8254630000 | -0.8093860000 | 0.0000390000 |
| H | 5.4361460000 | 1.6290710000 | 0.0000410000 |
| H | 1.1557610000 | -2.5073700000 | 0.0000190000 |

**(*E*)-1·H⁺ A**

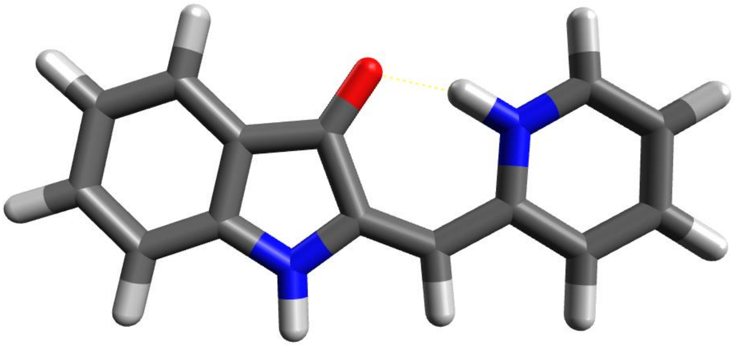

**Figure S113.** The optimized geometry of (*E*)-**1·H**$^+$ A calculated at B3LYP 6-311++G(d,p) level of theory with an IEFPCM $CHCl_3$ solvent model. Color codes; O = red; N = blue.

Zero-point correction= 0.220891 (Hartree/Particle)
Thermal correction to Energy= 0.233602
Thermal correction to Enthalpy= 0.234546
Thermal correction to Gibbs Free Energy= 0.181127
Sum of electronic and zero-point Energies= -724.655508
Sum of electronic and thermal Energies= -724.642797
Sum of electronic and thermal Enthalpies= -724.641853
Sum of electronic and thermal Free Energies= -724.695272

| Atom | X | Y | Z |
|---|---|---|---|
| O | -0.0323900000 | 1.6800980000 | -0.0204510000 |
| N | 2.4544320000 | 0.7826990000 | -0.0174050000 |
| N | -1.3051410000 | -1.5796480000 | -0.0225300000 |
| C | 3.5505810000 | -1.3076050000 | 0.0226820000 |
| C | 4.7706420000 | -0.6528970000 | 0.0317400000 |
| C | 4.8189160000 | 0.7460480000 | 0.0144520000 |
| C | 3.6288660000 | 1.4406260000 | -0.0085500000 |
| C | 2.3520940000 | -0.5743510000 | -0.0048270000 |
| C | 1.0826130000 | -1.2541430000 | -0.0179890000 |
| C | -0.7214320000 | 0.6593010000 | -0.0139150000 |
| C | -0.1929640000 | -0.7723460000 | -0.0202130000 |
| C | -3.1719620000 | 1.5084240000 | 0.0136540000 |
| C | -2.1643400000 | 0.5384880000 | -0.0018160000 |
| C | -2.4802650000 | -0.8330030000 | -0.0070470000 |
| C | -3.7988830000 | -1.2707570000 | 0.0033840000 |
| C | -4.7926930000 | -0.2909830000 | 0.0197430000 |
| C | -4.4929630000 | 1.0819600000 | 0.0247840000 |
| H | 3.5029000000 | -2.3881740000 | 0.0396520000 |
| H | 5.6884980000 | -1.2272660000 | 0.0527990000 |
| H | 5.7569580000 | 1.2825950000 | 0.0203420000 |
| H | 3.5673730000 | 2.5202020000 | -0.0201190000 |
| H | 1.1818590000 | -2.3341890000 | -0.0231640000 |
| H | -2.9182540000 | 2.5615990000 | 0.0176450000 |
| H | -4.0506740000 | -2.3240680000 | -0.0001440000 |
| H | -5.8306930000 | -0.6028310000 | 0.0289150000 |
| H | -5.2987280000 | 1.8047700000 | 0.0376980000 |
| H | -1.2733510000 | -2.5882310000 | -0.0171530000 |
| H | 1.5589220000 | 1.3208860000 | -0.0298180000 |

**(*E*)-1·H⁺ B**

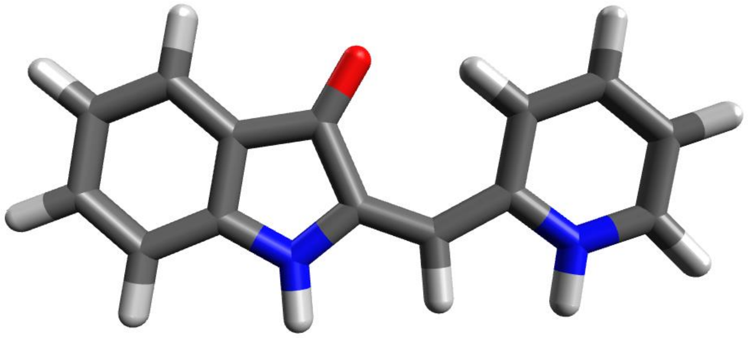

**Figure S114.** The optimized geometry of (*E*)-**1·H⁺** B calculated at B3LYP 6-311++G(d,p) level of theory with an IEFPCM $CHCl_3$ solvent model. Color codes; O = red; N = blue.

Zero-point correction= 0.221677 (Hartree/Particle)

Thermal correction to Energy= 0.233739

Thermal correction to Enthalpy= 0.234684

Thermal correction to Gibbs Free Energy= 0.183028

Sum of electronic and zero-point Energies= -724.641698gyh

Sum of electronic and thermal Energies= -724.629635

Sum of electronic and thermal Enthalpies= -724.628691

Sum of electronic and thermal Free Energies= -724.680346

| Atom | X | Y | Z |
|---|---|---|---|
| O | -0.2107320000 | 1.8307370000 | -0.0000150000 |
| N | 3.4519350000 | -1.2295160000 | 0.0000480000 |
| N | -1.2706130000 | -1.4991910000 | -0.0000630000 |
| C | 4.7317630000 | -0.7992500000 | 0.0000880000 |
| C | 5.0007430000 | 0.5480000000 | 0.0000480000 |
| C | 3.9164900000 | 1.4357580000 | -0.0000330000 |
| C | 2.6125570000 | 0.9706850000 | -0.0000700000 |
| C | 2.3507300000 | -0.4106730000 | -0.0000260000 |
| C | 1.0863740000 | -1.0929560000 | -0.0000530000 |
| C | -0.8180470000 | 0.7764790000 | -0.0000130000 |
| C | -0.2027750000 | -0.6395020000 | -0.0000510000 |
| C | -3.3253120000 | 1.4617420000 | 0.0000460000 |
| C | -2.2625670000 | 0.5580780000 | 0.0000070000 |
| C | -2.4937520000 | -0.8244550000 | -0.0000210000 |
| C | -3.7792140000 | -1.3465380000 | -0.0000060000 |
| C | -4.8358630000 | -0.4320810000 | 0.0000350000 |
| C | -4.6208720000 | 0.9538710000 | 0.0000600000 |
| H | 5.4922720000 | -1.5668180000 | 0.0001510000 |
| H | 6.0240480000 | 0.8948870000 | 0.0000790000 |
| H | 4.0958750000 | 2.5039950000 | -0.0000680000 |
| H | 1.7745230000 | 1.6502610000 | -0.0001270000 |
| H | 1.1755310000 | -2.1767220000 | -0.0000660000 |
| H | -3.1371560000 | 2.5286700000 | 0.0000660000 |
| H | -3.9611570000 | -2.4137120000 | -0.0000250000 |
| H | -5.8519820000 | -0.8092210000 | 0.0000480000 |
| H | -5.4688740000 | 1.6269670000 | 0.0000920000 |
| H | -1.1887220000 | -2.5055410000 | -0.0000730000 |
| H | 3.3007190000 | -2.2326730000 | 0.0000790000 |

**TD-DFT of 1**

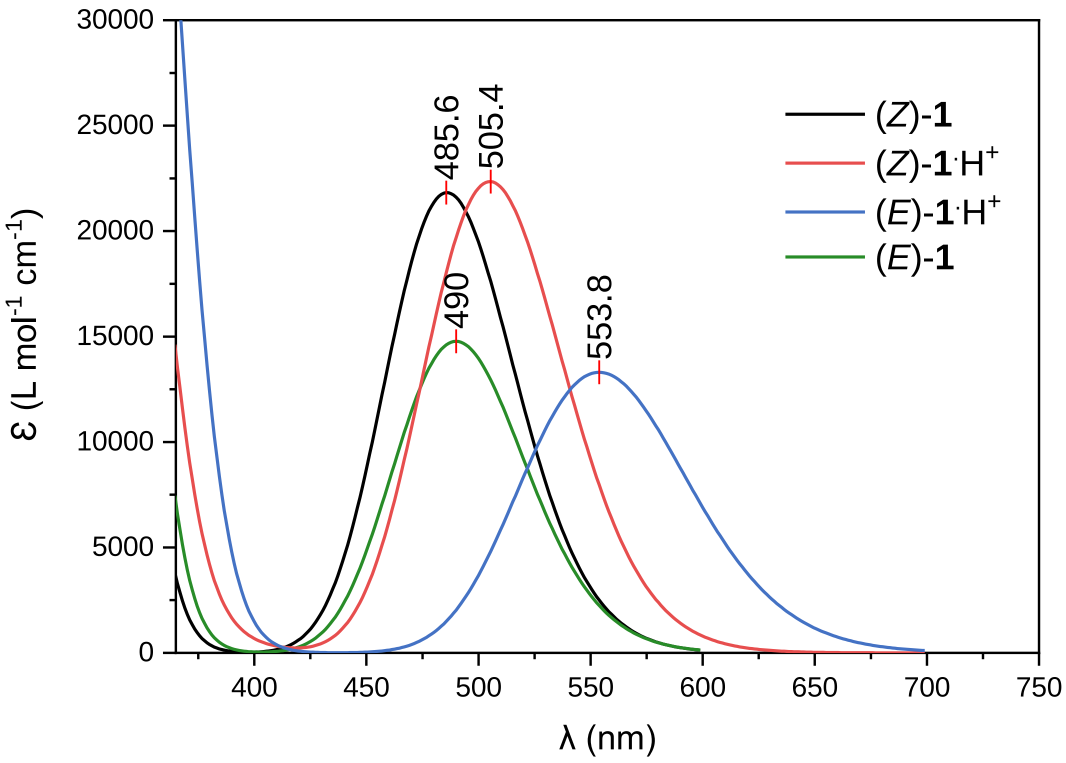


**Figure S115.** Predicted UV-Vis spectra of optimized (*Z*)-**1** A, (*E*)-**1** B, (*Z*)-**1**·**H**$^+$ B, and (*E*)-**1**·**H**$^+$ A (n=30 states, TD-DFT, B3LYP 6-311++G(d,p), IEF-PCM $CHCl_3$) with line-broadening set at 0.177 eV with peaks annotated.

## 12. References


1. Yamashita, M.; Shimizu, K.; Koizumi, Y.; Wakimoto, T.; Hamashima, Y.; Asakawa, T.; Inai, M.; Kan, T. Concise synthesis of anserine: efficient solvent tuning in asymmetric hydrogenation reaction. *Synlett* **2016**, *27*, 2734–2736.
2. Saito, R.; Kuchimaru, T.; Higashi, S.; Lu, S. W.; Kiyama, M.; Iwano, S.; Obata, R.; Hirano, T.; Kizaka-Kondoh, S.; Maki, S. A. Synthesis and luminescence properties of near-infrared N-heterocyclic luciferin analogues for in vivo optical imaging. *Bull. Chem. Soc. Jpn.* **2019**, *92*, 608–618.
3. Burger, U.; Bringhen, A. O. Cyclization studies with *N-Mannich* bases of 2-substituted indoles. *Helv. Chim. Acta* **1989**, *72*, 93–100.
4. Petermayer, C.; Thumser, S.; Kink, F.; Mayer, P.; Dube, H. Hemiindigo: highly bistable photoswitching at the biooptical window. *J. Am. Chem. Soc.* **2017**, *139*, 15060-15067.
5. Ikegami, M.; Arai, T. Photoisomerization and fluorescence properties of hemiindigo compounds having intramolecular hydrogen bonding. *Bull. Chem. Soc. Jpn.* **2003**, *76*, 1783-1792.
6. Shu, C.; Li, L.; Xiao, X.-Y.; Yu, Y.-F. ; Ping, Y.-F. ; Zhou, J.-M.; Ye, L.-W. Flexible and practical synthesis of 3-oxyindoles through gold-catalyzed intermolecular oxidation of *o*-ethynylanilines. *Chem. Commun.* **2014**, *50*, 8689–8692.
7. Sheldrick, G. M. Crystal structure refinement with SHELXL. *Acta Crystallogr., Sect. C: Struct. Chem.* **2015**, *71*, 3–8.
8. Montalti, M.; Credi, A.; Prodi, L.; Gandolfi, M. T. *Handbook of Photochemistry,* 3rd ed.; CRC Press, Boca Raton, Fl, 2006.
9. Rabani, J.; Mamane, H.; Pousty, D.; Bolton, J. R. Practical Chemical Actinometry—A Review. *Photochem. Photobiol.* **2021**, *97*, 873–902.
10. Langford, C. H.; Holubov, C. A. Wavelength and Temperature Dependence in the Photolysis of the Chemical Actinometer, Potassium Trisoxalatoferrate(III), at Longer Wavelengths. *Inorganica Chim. Acta* **1981**, *53*, 59–60.
11. Gaussian 09, Revision D.01, Frisch, M. J.; Trucks, G. W.; Schlegel, H. B.; Scuseria, G. E.; Robb, M. A.; Cheeseman, J. R.; Scalmani, G.; Barone, V.; Mennucci, B.; Petersson, G. A.; Nakatsuji, H.; Caricato, M.; Li, X.; Hratchian, H. P.; Izmaylov, A. F.; Bloino, J.;

Zheng, G.; Sonnenberg, J. L.; Hada, M.; Ehara, M.; Toyota, K.; Fukuda, R.; Hasegawa, J.; Ishida, M.; Nakajima, T.; Honda, Y.; Kitao, O.; Nakai, H.; Vreven, T.; Montgomery, Jr., J. A.; Peralta, J. E.; Ogliaro, F.; Bearpark, M.; Heyd, J. J.; Brothers, E.; Kudin, K. N.; Staroverov, V. N.; Keith, T.; Kobayashi, R.; Normand, J.; Raghavachari, K.; Rendell, A.; Burant, J. C.; Iyengar, S. S.; Tomasi, J.; Cossi, M.; Rega, N.; Millam, J. M.; Klene, M.; Knox, J. E.; Cross, J. B.; Bakken, V.; Adamo, C.; Jaramillo, J.; Gomperts, R.; Stratmann, R. E.; Yazyev, O.; Austin, A. J.; Cammi, R.; Pomelli, C.; Ochterski, J. W.; Martin, R. L.; Morokuma, K.; Zakrzewski, V. G.; Voth, G. A.; Salvador, P.; Dannenberg, J. J.; Dapprich, S.; Daniels, A. D.; Farkas, O.; Foresman, J. B.; Ortiz, J. V.; Cioslowski, J.; Fox, D. J.; Gaussian, Inc., Wallingford CT, 2013.

12. Avogadro: an open-source molecular builder and visualization tool. Version 1.2.0 http://avogadro.cc/

13. GaussView, Version 6, Dennington, Roy; Keith, Todd A.; Millam, John M. Semichem Inc., Shawnee Mission, KS, 2016.

14. Novir, S. B. DFT and TDDFT study of some bifunctional hemithioindigo chromophores. *Chem. Phys. Lett.* **2017**, *690*, 86–100.

15. Laurent, A. D.; André, J.-M.; Perpète, E. A ; Jacquemin, D. Hemi-indigo photochroms: a theoretical investigation. *Chem. Phys. Lett.* **2007**, *436*, 84–88.
